# New Polyketides from the Marine-Derived Fungus *Letendraea* Sp. 5XNZ4-2

**DOI:** 10.3390/md18010018

**Published:** 2019-12-24

**Authors:** Yan Xu, Ruibao Huang, Hongwei Liu, Tingting Yan, Wanjing Ding, Yongjun Jiang, Pinmei Wang, Daoqiong Zheng, Jinzhong Xu

**Affiliations:** 1 Ocean College, Zhoushan Campus, Zhejiang University, Zhoushan 316021, China; xuyan875@126.com (Y.X.); fau_rhuang@fau.edu (R.H.); ytt706@126.com (T.Y.); wading@zju.edu.cn (W.D.); yongjunjiang89@hotmail.com (Y.J.); wangpinmei@zju.edu.cn (P.W.); zhengdaoqiong@zju.edu.cn (D.Z.); 2 State Key Laboratory of Mycology, Institute of Microbiology, Chinese Academy of Sciences, Beijing 100101, China; liuhw@im.ac.cn

**Keywords:** marine-derived fungus, Letendraea sp., polyketide, phomopsiketone, letendronol

## Abstract

Marine-derived fungi have been reported to have great potential to produce structurally unique metabolites. Our investigation on secondary metabolites from marine-derived fungi resulted in the isolation of seven new polyketides (phomopsiketones D–G (**1**–**4**) and letendronols A–C (**5**–**7**)) as well as one known xylarinol (**8**) in the cultural broth of *Letendraea* sp. Their structures and absolute configurations were elucidated using a set of spectroscopic and chemical methods, including HRESIMS, NMR, single-crystal X-ray diffraction, ECD calculation, and a modified version of Mosher’s method. Compound **2** showed weak inhibition against nitric oxide production in lipopolysaccaride-activated macrophages with an IC_50_ value of 86 μM.

## 1. Introduction

Metabolites from marine-derived microorganisms have become important pharmacological resources [1,2]. At least 2000 novel natural products have been identified in the past five years, nearly 50% of which were derived from marine-derived fungi [3,4,5]. In addition, the metabolic pathways of marine-derived fungi, which can be attributed to unique marine habitats, especially those that contain sea salts, may be significantly different from terrestrial ones [6]. Thus, marine-derived fungi have great potential to produce structurally unique metabolites and have attracted a considerable amount of attention.

**Figure 1:**
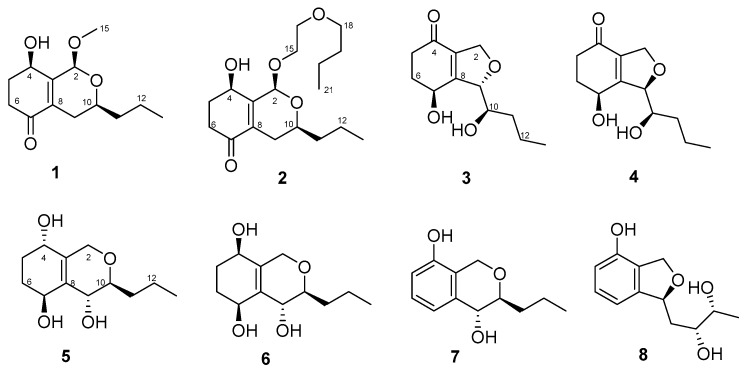
Chemical structures of compounds **1**–**8** isolated from *Letendraea* sp. 5XNZ4-2.

In our ongoing research on bioactive secondary metabolites from marine-derived fungi, we isolated an endophytic fungus *Letendraea* sp. 5XNZ4-2 from one sea crab. We obtained two novel spiroketals (letenketals A and B) from this fungus [7]. In order to obtain more structurally novel compounds, the systematic discovery of secondary metabolites for this strain was carried out. Seven new polyketides (phomopsiketones D–G (**1**–**4**) and letendronols A–C (**5**–**7**), see Figure 1) as well as one known xylarinol (**8**) were isolated from a Potato DFextrose Broth (1/2 PDB) cultural broth. Herein, we report the isolation, structure elucidation, and bioactivities of these compounds.

## 2. Results and Discussion

**Figure 2:**
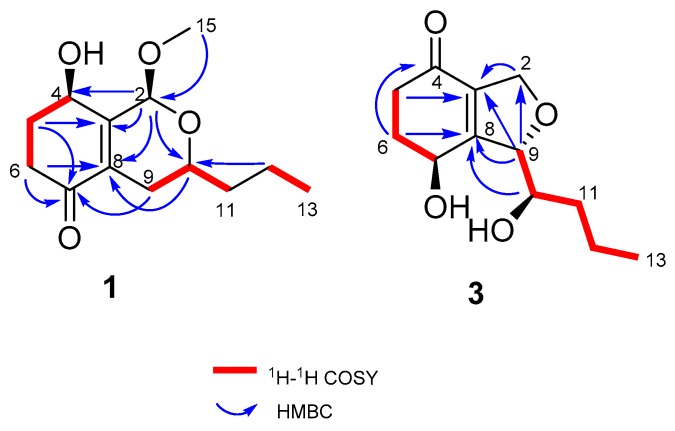
^1^H-^1^H COSY and key HMBC correlations of **1** and **3**.

Compound **1** was obtained as a yellow amorphous powder. Its molecular formula was determined to be C_13_H_20_O_4_ (with 4 degrees of unsaturation) by HRESIMS (*m/z* 263.1256 for [M+Na]^+^) and NMR data. IR absorption at 3426 and 1672 cm^−1^ indicated the presence of a hydroxyl and unsaturated ketones. ^1^H-NMR (Table 1) of **1** displayed one methoxy (*δ*_H_ 3.50, s) and one methyl (*δ*_H_ 0.95, t, *J* = 7.0 Hz). The ^13^C NMR and DEPT displayed 13 carbon signals, including one *α*, *β*- unsaturated ketone (*δ*_C_ 198.2), two olefinic carbons (*δ*_C_ 132.5, 151.4), one ketal carbon (*δ*_C_ 95.2), two oxygenated carbons (*δ*_C_ 65.3, 66.7), and one methoxy (*δ*_C_ 55.8). Calculation of unsaturation revealed that compound **1** contained a bicyclic skeleton. An *α*, *β*-unsaturated cyclohexanone moiety was derived from the ^1^H-^1^H Correlation Spectroscopy (COSY) correlations between H-4 (*δ*_H_ 4.59)/H_2_-5 (*δ*_H_ 2.06, 2.28)/H_2_-6 (*δ*_H_ 2.40, 2.70), coupled with the Heteronuclear Multiple Bond Correlation (HMBC) correlations from H_2_-5 to C-3 (δ*_C_* 151.4) and C-7 (δ*_C_* 198.2) and H_2_-6 to C-7 and C-8 (δ*_C_* 132.5) (Figure 2).

Two fragments of -CH_2_(12)-CH_3_(13) and -CH_2_(11)-CHO(10)-CH_2_(9)-, derived from ^1^H-^1^H COSY correlations of H_3_-13 (*δ*_H_ 0.95)/H_2_-12 (*δ*_H_ 1.43, 1.52) and H_2_-9 (*δ*_H_ 1.88, 2.36)/H-10 (*δ*_H_ 3.74)/H_2_-11 (*δ*_H_ 1.55, 1.61), were connected by the HMBC correlation from H_2_-12 to C-10 (δ*_C_* 66.7) as one aliphatic chain. Then, this aliphatic chain of -CH_2_ (9)-CHO (10)-CH_2_ (11)-CH_2_ (12)-CH_3_ (13) was positioned at C-8 according to the HMBC correlations from H-9 to C-7 and H-10 to C-8. C-2 was connected with C-3 (*δ_C_* 151.4) because of the HMBC correlations from H-2 to C-8, C-3, and C-4 (*δ_C_* 65.3), and the oxygen bridge between C-2 and C-10 was determined by the HMBC correlation from H-2 to C-10. The methoxy was positioned at C-2 by the HMBC correlation from H_3_-15 (*δ*_H_ 3.50) to C-2 (*δ*_C_ 95.2). Compound **1** was determined to be a 5,6-deoxy and 2-methoxyated derivative of EI-1941-1 [8] and named phomopsiketone D.

**Figure 3:**
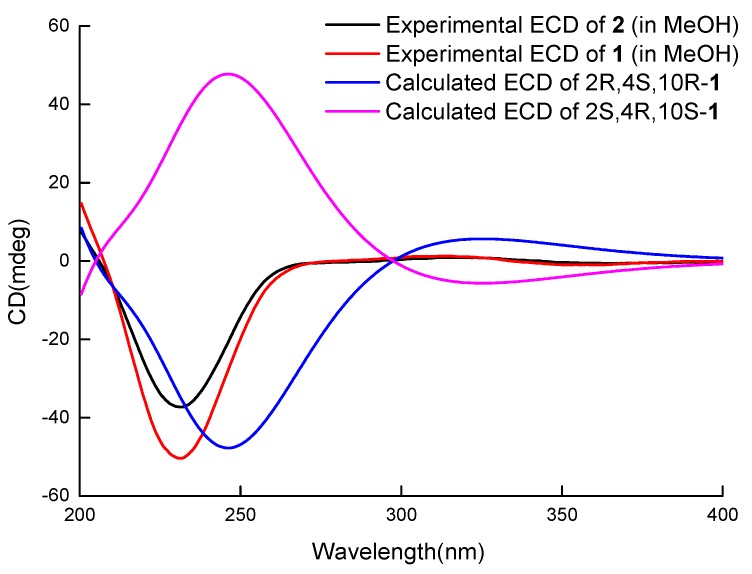
Comparison between calculated ECD spectra of **1** and experimental curves of **1**–**2**.

In order to establish the relative configuration of **1**, the Nuclear Overhauser Effect Spectroscopy (NOESY) was measured (Figure S15). NOESY correlations between H-2/H-10 and H-6a/H-4 suggest that both of the cyclohexanone and dihydropyran (DHP) rings were in the twist chair conformation. The cross peaks between H-4/H-2/H-10 suggested that they adopted the same orientations. The absolute configuration of **1** was established by a comparison between the experimental ECD spectrum and the theoretically calculated values of two possible stereoisomers (2*R*, 4*S*, 10*R*)-**1** and (2*S*, 4*R*, 10*S*)-**1 [9,10]**. The experimental ECD (Figure 3) of **1** showed a positive Cotton effect at 325 nm and a negative Cotton effect at 230 nm, which matched well with the calculated value of (2*R*, 4*S*, 10*R*)-**1**, and contributed to determining the absolute configuration of **1** as (2*R*, 4*S*, 10*R*).

Compound **2** was obtained as a light brown oil. Its molecular formula was determined to be C_18_H_30_O_5_ (with 4 degrees of unsaturation) on the basis of its HRESIMS (*m/z* 349.1989 for [M+Na]^+^) and NMR data. IR absorption at 3426 and 1678 cm^−1^ indicated the presence of a hydroxyl and unsaturated ketones. Its ^13^C NMR and DEPT spectra (Table 2) showed distinctive signals, including one *α*, *β*-unsaturated ketone (*δ_C_* 197.9), two olefinic carbons (*δ_C_* 131.5, 154.4), one ketal carbon (*δ_C_* 95.6), and one oxygenated methine (*δ_C_* 67.4), similar to those of **1**, and indicated that compound **2** contained a similar bicyclic skeleton to that of **1**. Additional signals, including three oxygenated methylenes (*δ_C_* 68.4, 69.9, 71.5), two methylenes (δ*_C_* 31.5, 19.3), and one methyl (*δ_C_* 14.0), were observed, while the methoxy in **1** was absent in **2**. These additional signals were organized as one 2-butoxyethoxy group (CH_3_(21)-CH_2_(20)-CH_2_(19)-CH_2_(18)-O-CH_2_(16)-CH_2_(15)) by ^1^H-^1^H COSY correlations of H_2_-15 (*δ*_H_ 3.86, 3.97)/H_2_-16 (*δ*_H_ 3.60, 3.66) and H_2_-18 (*δ*_H_ 3.53)/H_2_-19 (*δ*_H_ 1.61, 1.63)/H_2_-20 (*δ*_H_ 1.36, 1.39)/H_3_-21 (*δ*_H_ 0.94), combined with the HMBC correlations from H_2_-18 to C-16 and H_2_-16 to C-18 (Figure S1). The 2-butoxyethoxy group was connected to C-2 through an ether linkage according to the HMBC correlation from H_2_-15 to C-2 (*δ*_C_ 95.6). Thus, compound **2** was elucidated as shown in Figure 1 and named phomopsiketone E.

The DHP and cyclohexanone rings were also in twist chair conformations according to the NOESY correlation between H-2 and H-10 as well as the H-6 axial and H-4. The ECD spectra (Figure 3) of **1** and **2** showed similar curves (the cotton effect was positive at 325 nm (**1**) and 325 nm (**2**), and negative at 230 nm (**1**) and 228 nm (**2**)), and determined the absolute configurations of (2*R*, 4*S*, 10*R*) for **2**.

Compound **3** was obtained as a yellow oil. Its molecular formula was determined to be C_12_H_18_O_4_ (with 4 degrees of unsaturation) on the basis of its HRESIMS (*m/z* 261.0895 for [M+Cl]^-^) and NMR data. Carbon NMR (Table 2) showed 12 signals, including one *α*, *β*-unsaturated ketone (*δ*_C_ 197.1), two olefinic carbons (*δ_C_* 136.0, 163.6), nine aliphatic carbons that were grouped into one methyl (*δ_C_* 14.4), five methylenes (*δ_C_* 20.0, 34.5, 35.6, 37.1, and 73.8 (oxygenated)), and three oxygenated methines (*δ_C_* 66.0, 74.4, 91.3), by an Heteronuclear singular Quantum Coherence (HSQC) spectrum. These signals were similar to those of phomopsiketone A [11] except that one methylene in **3** replaced one oxygenated methine in phomopsiketone A. Several NMR signals, including a multiplet for H-7 (*δ*_H_ 4.67) (Table 1), and ^1^H-^1^H COSY correlations of H-7/H_2_-6/H_2_-5 (Figure 2) indicated the presence of 6-CH_2_. Thus, **3** was determined to be 6-deoxy-phomopsiketone A and named phomopsiketone F.

**Figure 4:**
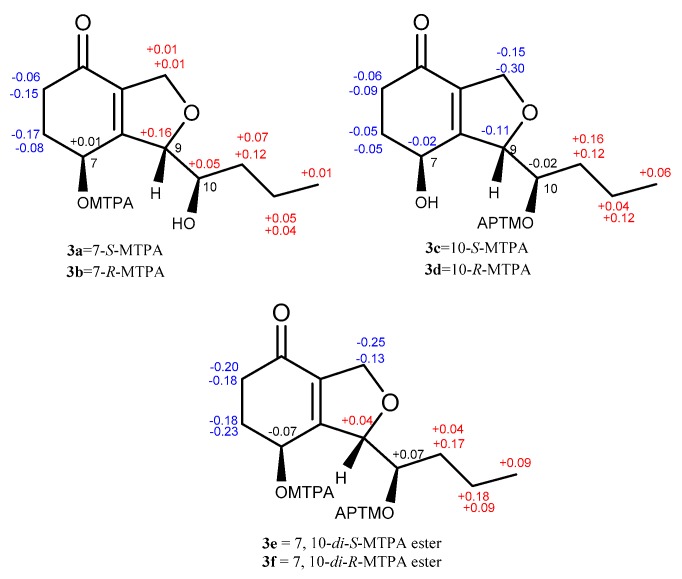
Δδ *_S_*_-*R*_ values for the MTPA esters (**3a**/**3b**, **3c**/**3d**, and **3e**/**3f**).

The absolute configuration of **3** was determined by a modified version of Mosher’s esterification method [12,13]. Compound **3** was acylated with R-(−)-and S-(+)-α-methoxy-α (trifluoromethyl) phenylacetyl chloride (α-MTPA-Cl). The products were separated by preparative HPLC and purified as six compounds, which were determined to be the 7-*S*-MTPA ester (**3a**), 7-*R*-MTPA ester (**3b**), 10-*S*-MTPA ester (**3c**), 10-*R*-MTPA ester (**3d**), 7,10-*di*-*S*-MTPA ester (**3e**), and 7,10-*di*-*R*-MTPA ester (**3f**) by HRESIMS (7,10-*S*-MTPA ester, *m/z* 465.1500; 7,10-*R*-MTPA ester, *m/z* 465.1497; 7,10-*di*-*S*-MTPA ester, *m/z* 681.1896; 7,10-*di*-*R*-MTPA ester, *m/z* 681.1893 for [M+Na]^+^, Figures S86, S87, S89, S90, S93 and S94, respectively), ^1^H NMR data (*δ*_H-7_ 6.14 for **3a**, 6.13 for **3b**; *δ*_H-10_ 5.40 for **3c**, 5.42 for **3d**, Figures S84 and S88, respectively), and the ^1^H-^1^H COSY correlations (Figure S85) of H-7 (*δ*_H_ 6.14)/H-6 (*δ*_H_ 2.09, 2.47) and H-10 (*δ*_H_ 3.67)/H-11 (*δ*_H_ 1.55, 1.44) in **3a**. A comparison of proton chemical shifts between 3**a**/3**b** and 3**c**/3**d** (Δ*δ***_3a_**_-**3b**_ and Δ*δ***_3c_**_-**3d**_**,** Figure 4) determined the configurations of C-7 and C-10 to be 7*S* and 10*R*, respectively. A NOESY correlation between H-7 (*δ*_H_ 5.92) and H-10 (*δ*_H_ 5.31) was observed in 3**f** and allowed us to deduce the configuration of C-9 as *S* (Figures S5 and S92). Thus, the absolute configuration of **3** was determined to be (7*S*, 9*S*, 10*R*).

Compound **4** was also obtained as a yellow oil. Its molecular formula was established as C_12_H_18_O_4_, identical to that of **3**, by HRESIMS (*m/z* 249.1101 for [M+Na]^+^) and NMR data. The proton and carbon spectra were also similar to those of **3** (Table 1 and Table 2) and a two-dimensional (2D) NMR analysis (Figure S2) enabled us to deduce that it has the same planar structure as **3**. Different ECD spectra of **3** and **4** (Figure S8) were used to confirm that they were stereoisomers.

A NOESY correlation between H-7 and H-9 was not observed. Then, the modified version of Mosher’s esterification method was applied to determine the absolute configuration of **4**. Esterification of **4** with (*R*)- and (*S*)-MTPA-Cl yielded the 7,10-*di-S*-MTPA ester (**4a**) and the 7,10-*di-R*-MTPA ester **(4b**), respectively. The similar ^1^H NMR chemical shift differences (Δδ**_4e_**_-**4f**_, Figure S6 and Figure 3) around the C-7 and C-10 stereocenters as **3** indicated the 7*S* and 10*R* configurations for **4**. Since **4** was the stereoisomer of **3,** the configuration of C-9 in **4** was deduced to be *R*, contrary to that in **3**. Thus, the absolute configuration of **4** was determined to be (7*S*, 9*R*, 10*R*) and it was named phomopsiketone G.

Compound **5** was crystallized as colorless needles in acetone. Its HRESIMS showed an ion peak [M+Na]^+^ at *m/z* 251.1258, corresponding to the molecular formula C_12_H_20_O_4_ and possessing two hydrogen atoms more than **3** and **4**. A comparison of ^13^C NMR data between **3** and **5** revealed that the ketone carbonyl (*δ*_C_ 197.1, C-4 in **3**) was replaced by one oxygenated methine (Table 2). ^1^H-^1^H COSY correlations between H-4 (*δ*_H_ 4.06)/H-5 (*δ*_H_ 1.54, 2.02)/H-6 (*δ*_H_ 1.56, 2.07)/ H-7(*δ*_H_ 4.43) (Figure S3) confirmed that the ketone carbonyl was hydrogenated. A similar *C*_5_ side chain (CH_3_-13/CH_2_-12/CH_2_-11/CH-10/CH-9) to that in **3** was derived from ^1^H-^1^H COSY correlations (Figure S3). Key HMBC correlations from H-2 (*δ*_H_ 4.02) to C-10 (*δ*_C_ 80.4) and H-9 (*δ*_H_ 3.99) to C-3 (*δ*_C_ 137.1) (Figure S3) indicated the presence of a dihydropyran fragment that was different from the dihydrofuran in **3**. Thus, **5** was determined to be a new polyketide and named letendronol A.

**Figure 5:**
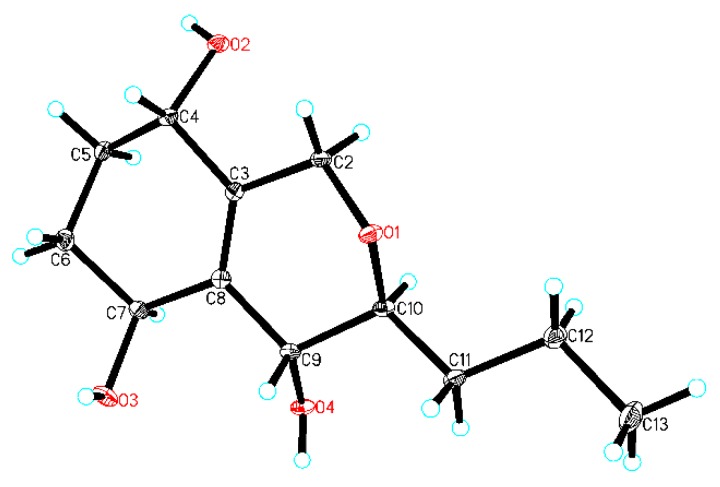
The X-ray crystal structure of **5** (the thermal ellipsoid was 30%).

In order to establish the relative configuration, **5** was crystallized and, by use of X-ray analysis (Figure 5), H-4 and H-9 were assigned to the same orientation, and H-7 and H-10 were assigned to the opposite orientation of H-4 and H-9.

The *tri*-(*S*/*R*) MTPA esters (**5a**/**5b**) were obtained by the modified version of Mosher’s method together with HPLC purification. A ^1^H NMR data comparison of **5a** and **5b** (Δδ**_5a_**_-**5b**_, Figure S7) determined the absolute configurations of (4*S*, 7*S*, 9*R*) in **5**. Finally, the configuration of **5** was unambiguously confirmed to be (4*S*, 7*S*, 9*R*, and 10*S*) by X-ray analysis combined with the modified version of Mosher’s method.

Compound **6** was contained as a colorless amorphous powder. It has the same molecular formula (C_12_H_20_O_4_) as **5** according to its HRESIMS (*m/z* 251.1260 for [M+Na]^+^) and ^13^C NMR data. One dimensional (1D) NMR data (Table 1 and Table 2) combined with a 2D NMR analysis (Figure S3) revealed **6** to have the same planar structure as **5**. Compound **6** was deduced to be a stereoisomer of **5** because of their different retention times when eluted by the same HPLC program and was named letendronol B.

Compounds **6** and **5** contained different a configuration at C-4, which was assigned by the modified version of Mosher’s method. Treatment of **6** with (*R*)- or (*S*)-MTPA-Cl yielded the *tri*-(*S*/*R*) MTPA esters (**6a**/**6b**). The result of the application of the modified version of Mosher’s method (Δδ**_6a_**_-**6b**_, Figure S7) revealed 7*S* and 9*R* configurations, identical to **5**, and a 4*R* configuration different from **5**, in **6**. H-9 and H-10 were also assigned to different orientations according to the broad singlet peaks for H-9 in **6a** (*δ*_H_ 5.11, br s) and **6b** (*δ*_H_ 4.78, br s) (Figure S110), similar to those in **5a** (*δ*_H_ 5.19, br s) and **5b** (*δ*_H_ 4.76, br s) (Figure S98). Then, the absolute configuration of **6** was determined to be (4*R*, 7*S*, 9*R*, and 10*S*).

**Figure 6:**
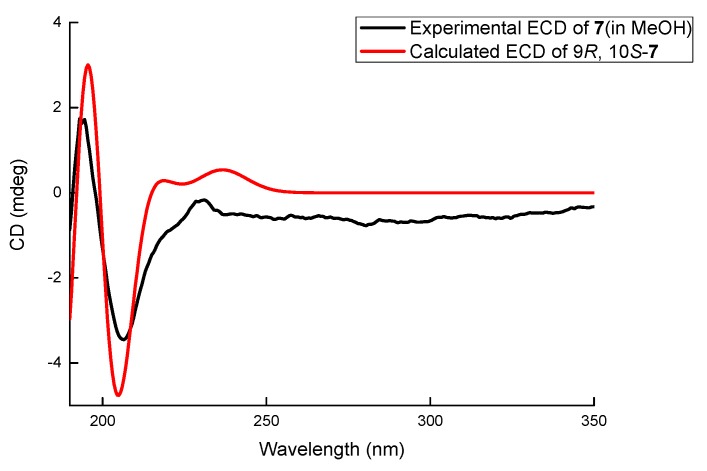
The comparison between calculated ECD spectra and the experimental curve of **7**.

Compound **7** was obtained as a white amorphous powder. The molecular formula of **7** was determined to be C_12_H_16_O_3_ with 5 degrees of unsaturation by its HRESIMS (*m/z* 231.0995 for [M+Na]^+^) and NMR data. The ^1^H NMR (Table 1) of **7** displayed one methyl (*δ*_H_ 0.99, 3H, t, *J* = 7.2 Hz) and three aromatic protons (*δ*_H_ 6.63, 1H, d, *J* = 8.0 Hz; δ*_H_* 7.07, 1H, t, *J* = 8.0 Hz; *δ*_H_ 7.01, 1H, d, *J* = 8.0 Hz) whose coupling constants revealed their *ortho*-relationship. The ^13^C NMR spectrum (Table 2) showed 12 signals, which were assigned by HSQC data to six aromatic carbons (*δ*_C_ 153.5, 113.8, 128.3, 118.9, 140.0, and 123.3), two oxygenated methines (*δ*_C_ 80.4, 70.1), one oxygenated methylene (*δ*_C_ 65.3), two methylenes (*δ*_C_ 35.5 and 19.8), and one methyl carbon (*δ*_C_ 14.4). A similar *C*_5_ side chain to those in **3**–**6** was derived from ^1^H-^1^H COSY correlations (Figure S4) between H-13 (*δ*_H_ 0.99)/H_2_-12 (*δ*_H_ 1.48, 1.65)/H_2_-11 (*δ*_H_ 1.50, 1.89)/H-10 (*δ*_H_ 3.37)/H-9 (*δ*_H_ 4.30) and connected with the aromatic ring at C-8 (*δ*_C_ 140.0) according to the HMBC correlations from H-9 to C-8 (*δ*_C_ 140.0), C-3 (*δ*_C_ 123.3), and C-7 (*δ*_C_ 118.9). Meanwhile, oxygenated CH_2_ (*δ*_C_ 65.3, C-2) was connected with C-3 (*δ*_C_ 123.3) because of the HMBC correlations from H_2_-2 (*δ*_H_ 4.60, 4.82) to C-3, C-8, and C-4 (*δ*_C_ 153.5). The oxygen bridge between C-2 and C-10 (*δ*_C_ 80.4) was determined by the HMBC correlation from H-2 to C-10. Thus, compound **7** was determined to be a new polyketide containing an isochroman skeleton and named letendronol C. The vicinal coupling constant *J*_H-9/H-10_ (8.5 Hz) indicated a *trans* relationship between H-9 and H-10. A comparison of the optical rotation of **7** ([α]^20^_D_ +9) with that of (3*R*,4*S*)-6,8-Dihydroxy-1,1-dimethyl-3,4,5-trimethylisochroman [14] ([α]^25^_D_ -28.5) was used to determine that the absolute configurations at C-9 and C-10 were *R* and *S*, respectively. The 9*R* and 10*S* configurations of **7** were also confirmed by a comparison between the experimental ECD spectrum and the theoretically calculated values of (9*R*, 10*S*)-**7**. The experimental ECD (Figure 6) of **7** showed a positive Cotton effect at 195 and a negative Cotton effect at 205 nm, which matched well with the calculated value of (9*R*, 10*S*)-**7**.

The known compound **8** was determined to be xylarinol by comparison of its NMR data with the reported literature [15].

Compounds **1**, **2**, **3**, and **5** were evaluated for their inhibitory activities against Lipopolysaccharide (LPS)-activated NO production in RAW264.7 [16], and all tested compounds showed no cytotoxicity to macrophage cells at the concentration of 100 μM and 50 μM by the MTT method. Compound **2** showed weak anti-inflammatory activity with an IC_50_ value of 86 μΜ. Hydrocortisone was used as a positive control with the IC_50_ value of 22.4 μM. The other compounds showed no anti-inflammatory activity with IC_50_ values of >100 μM. All compounds were assayed for their cytotoxicity against the prostate cancer PC3 cell line by the MTT method [17]. Compounds **1**–**3**, **5**, **6**, and **8** were also assayed for antibacterial activity against *Staphylococcus aureus*, *Staphylococcus epidermidis*, *Escherichia coli*, *Miconia albicans* and *Pseudomonas aeruginosa* using the Kirby−Bauer Diffusion method. Regrettably, none of these compounds showed any activities in the cytotoxicity and antibacterial tests.

## 3. Materials and Methods

### 3.1. General Experimental Procedures

Optical rotations were measured on Rudolph research analytical AUTOPOL I. The ultraviolet spectra were obtained from a Shimadzu UV-1800 spectrophotometer using MeOH as the solvent. Electronic circular dichroism (ECD) spectra were obtained on a JASCO J-1500 circular dichroism. The infrared (IR) spectra were acquired from a Bruker Vector 22. NMR spectra were recorded on a Bruker AVIII 500 MHz and JEOL 600 Hz, using TMS as the internal standard. HRESIMS spectra were obtained from an Agilent 6224 TOF LC/MS. Analytical HPLC was performed on an Agilent 1260 system using a C18 (Cosmosil, 5 μm, 4.6 × 250 mm) column. The column chromatography was carried out using Silica gel (200–300 mesh, Qing Dao Hai Yang Chemical Group Co., Qing Dao, China).

### 3.2. Fungal Material and Fermentation

The *Letendraea* sp. was isolated from the gut of a crab found on Zhairuoshan Island (N20.2920, E122.5), Zhoushan, Zhejiang Province, China, in August 2015. The fungus was determined to be *Letendraea* sp. by 26s rDNA sequence analysis (GenBank accession no. MK743951), and produced a sporulating, white-colored culture when growing on potato-dextrose agar (PDA). The strain was deposited (NO. 5XNZ4-2) at the Institute of Marine Biology of Zhejiang University. The strain was static cultured at 28 °C for 30 days in 500 mL Erlenmeyer flasks (100 × 200 mL, a total of 20 L) each containing 200 mL of 1/2 PDB liquid media (100 g of potato extract, 17 g of artificial sea salt, and 10 g of dextrose in 1 L pure water).

### 3.3. Extraction and Isolation

The culture broth (20 L) was filtered and extracted with equivoluminal EtOAc three times to obtain 2.79 g of metabolite extract, which was fractionated by column chromatography (CC) over silica gel (200−300 mesh, 60 g) and eluted in a gradient of petroleum ether (P)-EtOAc(E) (20:1–1:1) to yield 10 fractions (Frs. 1–10) based on TLC analysis. Fr. 7 was further separated via preparative HPLC with a C18 (Agilent, 10 μm, 21.2 × 250 mm) column at 210 nm, eluting with MeOH/H_2_O (40/60, v/v) at 8 mL/min to obtain four sub-fractions (Fr. 7.1–7.4). Fr. 7.1 (110 mg) was purified with semi-preparative HPLC (Agilent C18 column, 10 μm, 10 × 250 mm, MeCN/H_2_O 10:90, 4 mL/min, 210 nm) to yield **3** (73 mg). Fr. 10 was purified by CC over silica gel (200−300 mesh, 30 g) using a gradient of CH_2_Cl_2_-MeOH (50:1–1:1) as a mobile phase to provide five fractions (Fr. 11–15). Fr. 13 was also separated via preparative HPLC (Agilent C18 column, 10 μm, 21.2 × 250 mm, 210 nm), eluting with MeOH/H_2_O (15:85, v/v) at 10 mL/min to obtain **5** (148 mg) and other three sub-fractions (Fr. 13.1–13.3). Fr. 13.2 (64 mg) was further purified on semi-preparative HPLC (Agilent C18 column, 10 μm, 10 × 250 mm, MeOH/H_2_O 15:85, 4 mL/min, 210 nm) to yield **6** (14 mg). Fr. 8 was initially separated by CC over silica gel (200−300 mesh, 60 g) with a dichloromethane (D) and MeOH (M) gradient from 80:1 to 5:1 based on TLC analysis to afford 10 sub-fractions (Fr. 8.1–8.10). Sub-fractions Fr. 8.3 (142 mg), Fr. 8.5 (33 mg), Fr. 8.9 (24.2 mg), and Fr. 8.10 (45.7 mg) were all further purified by semi-preparative HPLC (Agilent C18 column, 10 μm, 10 × 250 mm, 230 nm) at 4 mL/min using MeCN/H_2_O (63/37, v/v), MeCN/H_2_O (25/75, v/v), MeOH/H_2_O (18/82, v/v), and MeOH/H_2_O (35/65, v/v) as the eluting solvents, respectively. Compounds **1** (16.4 mg) and **2** (19.2 mg) were acquired from Fr. 8.3. Compounds **7** (7.7 mg), **4** (2.7 mg), and **8** (19.4 mg) were obtained from Fr. 8.5, Fr. 8.9, and Fr. 8.10, respectively.

Phomopsiketone D (**1**): Yellow amorphous powder; molecular formula C_13_H_20_O_4_; [α] ^20^_D_ –60 (c 0.1, MeOH); ECD (0.50 mg/mL, MeOH) λmax (Δ ε) 350 (–0.97), 311 (+1.24), 230 (–50.40), 194 (+27.24) nm; UV (MeOH) λmax (log ε) 232 (3.93) nm; IR (λmax) 3426, 2957, 2933, 2874, 2830, 1672, 1422, 1380, 1332, 1280, 1189, 1172, 1095, 1066, 1040, 979, 950, 906 cm^−1^; ^1^H NMR data (500 MHz, in CDCl_3_) and ^13^C NMR data (125 MHz, in CDCl_3_), see Table 1 and Table 2; HRESIMS *m/z* [M+Na]^+^ 263.1256 (calcd. for C_13_H_20_O_4_Na, 263.1259).

Phomopsiketone E (**2**): Light brown oil; molecular formula C_18_H_30_O_5_; [α] ^20^_D_ –60 (c 0.1, MeOH); ECD (0.50 mg/mL, MeOH) λmax (Δ ε) 365 (–0.64), 311 (+0.87), 230 (–37.30), 194 (+15.31) nm; UV (MeOH) λmax (log ε) 232 (3.99) nm; IR (λmax) 3437, 2958, 2932, 2873, 1678, 1462, 1326, 1279, 1172, 1121, 1074, 1034, 924, 906 cm^−1^; ^1^H NMR data (500 MHz, in CDCl_3_) and ^13^C NMR data (125 MHz, in CDCl_3_), see Table 1 and Table 2; HRESIMS *m/z* [M+Na]^+^ 349.1989 (calcd. for C_18_H_30_O_5_Na, 349.1991).

Phomopsiketone F (**3**): Yellow oil; molecular formula C_12_H_18_O_4_; [α] ^20^_D_ –30 (c 0.1, MeOH); ECD (0.40 mg/mL, MeOH) λmax (Δ ε) 323 (+9.56), 259 (–48.72), 203 (+58.04) nm; UV (MeOH) λmax (log ε) 257 (3.75), 210 (3.80) nm; IR (λmax) 3437, 2961, 2870, 1671, 1542, 1398, 1124, 1071, 1026, 922, 849 cm^−1^; ^1^H NMR data (500 MHz, in CD_3_OD) and ^13^C NMR data (125 MHz, in CD_3_OD), see Table 1 and Table 2; HRESIMS *m/z* [M+Cl]^-^ 261.0895 (calcd. for C_12_H_18_O_4_Cl, 261.0894).

Phomopsiketone G (**4**): Yellow oil; molecular formula C_12_H_18_O_4_; [α] ^20^_D_ –2 (c 0.1, MeOH); ECD (0.45 mg/mL, MeOH) λmax (Δ ε) 322 (–2.76), 255 (–12.86), 204 (+34.78) nm; UV (MeOH) λmax (log ε) 225 (4.18) nm; IR (λmax) 3386, 2958, 2932, 2872, 1665, 1460, 1395, 1125, 1074, 1003, 947, 855 cm^−1^; ^1^H NMR data (600 MHz, in CD_3_OD) and ^13^C NMR data (150 MHz, in CD_3_OD), see Table 1 and Table 2; HRESIMS *m/z* [M+Na]^+^ 249.1101 (calcd. for C_12_H_18_O_4_Na, 249.1103).

Letendronol A (**5**): Colorless needle-like crystal from acetone; mp 159–160 °C; molecular formula C_12_H_20_O_4_; [α] ^20^_D_ –5 (c 0.1, MeOH); ECD (0.50 mg/mL, MeOH) λmax (Δ ε) 213 (–60.56), 194 (+41.96) nm; UV (MeOH) λmax (log ε) 263 (2.37) nm; IR (λmax) 3338, 3295, 2953, 2921, 2866, 1459, 1322, 1292, 1155, 1120, 1070, 1034, 1005, 924, 905 cm^−1^; ^1^H NMR data (500 MHz, in CD_3_OD) and ^13^C NMR data (125 MHz, in CD_3_OD), see Table 1 and Table 2; HRESIMS *m/z* [M+Na]^+^ 251.1258 (calcd. for C_12_H_20_O_4_Na, 251.1259).

Letendronol B (**6**): Colorless amorphous powder; molecular formula C_12_H_20_O_4_; [α] ^20^_D_ –3 (c 0.1, MeOH); ECD (0.40 mg/mL, MeOH) λmax (Δ ε) 214 (–20.71), 195 (+31.03) nm; UV (MeOH) λmax (log ε) 263 (2.37) nm; IR (λmax) 3354, 2956, 2872, 1447, 1332, 1280, 1186, 1152, 1102, 1054, 1005, 970, 948 cm^−1^; ^1^H NMR data (500 MHz, in CD_3_OD) and ^13^C NMR data (125 MHz, in CD_3_OD), see Table 1 and Table 2; HRESIMS *m/z* [M+Na]^+^ 251.1260 (calcd. for C_12_H_20_O_4_Na, 251.1259).

Letendronol C (**7**): White amorphous powder; molecular formula C_12_H_16_O_3_; [α] ^20^_D_ +9 (c 0.1, MeOH); ECD (0.40 mg/mL, MeOH) λmax (Δ ε) 280 (–0.77), 206 (–3.45) nm; UV (MeOH) λmax (log ε) 275 (3.03), 205 (3.96) nm; IR (λmax) 3354, 2958, 2930, 2872, 1593, 1469, 1366, 1280, 1230, 1090, 1049, 1015, 964, 898, 840, 803, 762 cm^−1^; ^1^H NMR data (500 MHz, in CD_3_OD) and ^13^C NMR data (125 MHz, in CD_3_OD), see Table 1 and Table 2; HRESIMS *m/z* [M+Na]^+^ 231.0995 (calcd. for C_12_H_16_O_3_Na, 231.0997).

### 3.4. ECD Calculation of Compound **1**

Monte Carlo conformational searches were carried out by means of the Spartan’s 10 software using Merck Molecular Force Field (MMFF). The conformers with a Boltzmann population of over 5% were chosen for ECD calculations, and then the conformers were initially optimized at the B3LYP/6-31+g (d, p) level in MeOH using the CPCM polarizable conductor calculation model. The theoretical calculation of ECD was conducted in MeOH using time-dependent density functional theory (TD-DFT) at the B3LYP/6-311+g (d, p) level for all conformers of compound **1**. Rotatory strengths for a total of 50 excited states were calculated. ECD spectra were generated using the program SpecDis 1.6 (University of Würzburg, Würzburg, Germany) and GraphPad Prism 5 (University of California San Diego, USA) from dipole-length rotational strengths by applying Gaussian band shapes with sigma = 0.3 eV [18].

### 3.5. Preparation of MTPA Esters of Compound **3**

Compound **3** (4 mg, 8.85 μmol each) in 1 mL of anhydrous pyridine was separated into two equal portions. (*R*)- or (*S*)-MTPA chloride (5 μL, 26.6 μmol each) was added into the two portions, respectively. Each reaction mixture was stirred at ambient temperature for 4 h and the reaction was terminated by adding 1 mL of methanol. The reaction mixtures were dried under vacuum and separated by HPLC (column: Agilent ODS column, 10.0 × 250 mm; mobile phase: MeOH-H_2_O, 70:30 (v/v); flow rate: 4 mL/min) to afford the 7-*S*-MTPA ester (**3a**, 0.56 mg), 7-*R*-MTPA ester (**3b**, 0.68 mg), 10-*S*-MTPA ester (**3c**, 1.02 mg), 10-*R*-MTPA ester (**3d**, 0.96 mg), 7,10-*di*-*S*-MTPA ester (**3e**, 0.73 mg), and 7,10-*di*-*R*-MTPA ester (**3f**, 0.85 mg) of **3**.

### 3.6. Preparation of MTPA Esters of Compounds **4**–**6**

Compounds **4** (1 mg), **5** (4 mg), and **6** (1.5 mg) in 1 mL of anhydrous pyridine were separated into two equal portions. (*R*)- or (*S*)-MTPA chloride (50 μL each) was added into each portion, respectively. Each reaction mixture was stirred at ambient temperature for 4 h and the reaction was terminated by adding 1 mL of methanol. HPLC was also used for the isolation of (*S*)-MTPA ester -**4** (**4a**, 0.7 mg) and (*R*)-MTPA ester -**4** (**4b**, 0.7 mg). The (*S*)-MTPA and (*R*)-MTPA esters of **5** (**5a**, 5.0 mg; **5b**, 5.7 mg) and **6** (**6a**, 2.30 mg; **6b**, 1.92 mg) were also prepared in a similar manner.

7-*S*-MTPA Ester of **3** (3**a**): White amorphous solid. ^1^H NMR (600 MHz, in CD_3_OD) *δ*_H_ 7.30−7.64 (5H, m, MTPA-Ar), 6.14 (1H, brs, H-7), 4.81 (1H, m, H-9), 4.73 (1H, ddd, *J* = 13.0, 6.0, 2.6 Hz, H-2a), 4.68 (1H, ddd, *J* = 13.0, 6.0, 1.6 Hz, H-2b), 3.67 (1H, m, H-10), 3.57 (3H, brs, MTPA-OMe), 2.51 (1H, m, H-5a), 2.47 (1H, m, H-6a), 2.38 (1H, m, H-5b), 2.09 (1H, m, H-6b), 1.58 (1H, m, H-12a), 1.55 (1H, m, H-11a), 1.44 (1H, m, H-11b), 1.39 (1H, m, H-12b), 0.94 (3H, t, *J* = 7.0 Hz, H-13); HRESIMS *m/z* [M+Na]^+^ 465.1500 (calcd. for C_22_H_25_F_3_O_6_Na, 465.1501).

7-*R*-MTPA Ester of **3** (3**b**): White amorphous solid. ^1^H NMR (600 MHz, in CD_3_OD) *δ*_H_ 7.32−7.64 (5H, m, MTPA-Ar), 6.13 (1H, brs, H-7), 4.72 (1H, ddd, *J* = 12.8, 6.0, 2.7 Hz, H-2a), 4.67 (1H, m, H-2b) 4.65 (1H, m, H-9), 3.62 (1H, m, H-10), 3.56 (3H, brs, MTPA-OMe), 2.57 (1H, m, H-5a), 2.55 (1H, m, H-6a), 2.53 (1H, m, H-5b), 2.26 (1H, m, H-6b), 1.54 (1H, m, H-12a), 1.43 (1H, m, H-11a), 1.37 (1H, m, H-11b), 1.34 (1H, m, H-12b), 0.93 (3H, t, *J* = 7.3 Hz, H-13); HRESIMS *m/z* [M+Na]^+^ 465.1497 (calcd. for C_22_H_25_F_3_O_6_Na, 465.1501).

10-*S*-MTPA Ester of **3** (3**c**): White amorphous solid. ^1^H NMR (600 MHz, in CD_3_OD) *δ*_H_ 7.30−7.64 (5H, m, MTPA-Ar), 5.40 (1H, ddd, *J* = 8.3, 5.6, 1.8 Hz, H-10), 5.30 (1H, m, H-9), 4.52 (1H, m, H-2a), 4.50 (1H, m, H-7), 4.35 (1H, ddd, *J* = 13.0, 5.7, 3.0 Hz, H-2b), 3.55 (3H, m, MTPA-OMe), 2.43 (1H, m, H-5a), 2.27 (1H, m, H-5b), 2.24 (1H, m, H-6a), 1.94 (1H, m, H-6b), 1.84 (1H, m, H-12a), 1.78 (1H, m, H-11a), 1.47 (1H, m, H-11b), 1.43 (1H, m, H-12b), 0.98 (3H, t, *J* = 7.4 Hz, H-13); HRESIMS *m/z* [M+Na]^+^ 465.1500 (calcd. for C_22_H_25_F_3_O_6_Na, 465.1501).

10-*R*-MTPA Ester of **3** (3**d**): White amorphous solid. ^1^H NMR (600 MHz, in CD_3_OD) *δ*_H_ 7.31−7.64 (5H, m, MTPA-Ar), 5.42 (1H, m, H-10), 5.41 (1H, m, H-9), 4.67 (1H, m, H-2a), 4.65 (1H, m, H-2b), 4.52 (1H, m, H-7), 3.54 (3H, d, *J*=1.0 Hz, MTPA-OMe), 2.49 (1H, m, H-5a), 2.36 (1H, m, H-5b), 2.29 (1H, m, H-6a), 1.99 (1H, m, H-6b), 1.80 (1H, m, H-12a), 1.62 (1H, m, H-11a), 1.35 (1H, m, H-11b), 1.31 (1H, m, H-12b), 0.92 (3H, t, *J* = 7.4 Hz, H-13); HRESIMS *m/z* [M+Na]^+^ 465.1497 (calcd. for C_22_H_25_F_3_O_6_Na, 465.1501).

7, 10-*di*-*S*-MTPA Ester of **3** (**3e**): White amorphous solid. ^1^H NMR (600 MHz, in CD_3_OD) δ*_H_* 7.39−7.52 (10H, m, MTPA-Ar), 5.85 (1H, m, H-7), 5.38 (1H, ddd, *J* = 9.2, 4.8, 1.8 Hz, H-10), 5.11 (1H, ddd, *J* = 6.0, 3.7, 1.8 Hz, H-9), 4.58 (1H, ddd, *J* = 13.3, 3.8, 1.0 Hz, H-2*α*), 4.45 (1H, ddd, *J* = 13.3, 5.9, 2.1 Hz, H-2*β*), 3.57 (3H, d, *J* = 1.0 Hz, MTPA-OMe), 3.52 (3H, d, *J* = 1.0 Hz, MTPA-OMe), 2.34 (1H, m, H-5*α*), 2.30 (1H, m, H-5*β*), 2.19 (1H, m, H-6*α*), 2.13 (1H, m, H-6*β*), 1.78 (1H, m, H-12*α*), 1.66 (1H, m, H-11*α*), 1.46 (1H, m, H-11*β*), 1.41 (1H, m, H-12*β*), 0.97 (3H, t, *J* = 7.3 Hz, H-13); HRESIMS *m/z* [M+Na]^+^ 681.1896 (calcd. for C_32_H_32_F_6_O_8_Na, 681.1899).

7, 10-*di*-*R*-MTPA Ester of **3** (**3f**): White amorphous solid. ^1^H NMR (600 MHz, in CD_3_OD) δ*_H_* 7.38−7.53 (10H, m, MTPA-Ar), 5.92 (1H, t, *J* = 4.9 Hz, H-7), 5.31 (1H, ddd, *J* = 10.3, 3.8, 2.0 Hz, H-10), 5.08 (1H, td, *J* = 4.8, 2.0 Hz, H-9), 4.71 (1H, m, H-2*α*), 4.69 (1H, m, H-2*β*), 3.58 (3H, d, J = 0.8 Hz, MTPA-OMe), 3.52 (3H, d, *J* = 0.8 Hz, MTPA-OMe), 2.54 (1H, m, H-5*α*), 2.48 (1H, m, H-5*β*), 2.42 (1H, m, H-6*α*), 2.31 (1H, m, H-6*β*), 1.69 (1H, m, H-12*α*), 1.43 (1H, m, H-11*α*), 1.29 (1H, m, H-11*β*), 1.23 (1H, m, H-12*β*), 0.88 (3H, t, *J* = 7.4 Hz, H-13); HRESIMS *m/z* [M+Na]^+^ 681.1893 (calcd. for C_32_H_32_F_6_O_8_Na, 681.1899).

*S*-MTPA Ester of **4** (4**a**): White amorphous solid. ^1^H NMR (600 MHz, in CD_3_OD) *δ*_H_ 7.39−7.48 (10H, m, MTPA-Ar), 5.96 (1H, m, H-7), 5.46 (1H, ddd, *J* = 9.0, 4.6, 1.9 Hz, H-10), 5.16 (1H, m, H-9), 4.72 (1H, *J* = 16.3, 3.6, 1.6 Hz,, H-2a), 4.65 (1H, *J* = 16.3, 5.9, 0.7 Hz,, H-2b), 3.49 (3H, s, MTPA-OMe), 3.38 (3H, s, MTPA-OMe), 2.54 (1H, m, H-5a), 2.49 (1H, m, H-5b), 2.47 (1H, m, H-6a), 2.03 (1H, m, H-6b), 1.70 (1H, m, H-12a), 1.52 (1H, m, H-11a), 1.36 (1H, m, H-11b), 1.30 (1H, m, H-12b), 0.88 (3H, t, *J* = 7.4 Hz, H-13); HRESIMS *m/z* [M+Na]^+^ 681.1898 (calcd. for C_32_H_32_F_6_O_8_Na, 681.1899).

*R*-MTPA Ester of **4** (4**b**): White amorphous solid. ^1^H NMR (600 MHz, in CD_3_OD) *δ*_H_ 7.38−7.53 (10H, m, MTPA-Ar), 6.02 (1H, m, H-7), 5.42 (1H, ddd, *J* = 10.3, 3.9, 1.9 Hz, H-10), 5.25 (1H, m, H-9), 4.67 (1H, *J* = 16.3, 3.7, 1.5 Hz,, H-2a), 4.48 (1H, *J* = 16.5, 5.9, 1.0 Hz,, H-2b), 3.54 (3H, brs, MTPA-OMe), 3.50 (3H, brs, MTPA-OMe), 2.59 (1H, m, H-5a), 2.55 (1H, m, H-5b), 2.51 (1H, m, H-6a), 2.20 (1H, m, H-6b), 1.58 (1H, m, H-12a), 1.28 (1H, m, H-11a), 1.16 (1H, m, H-11b), 1.09 (1H, m, H-12b), 0.76 (3H, t, *J* = 7.5 Hz, H-13); HRESIMS *m/z* [M+Na]^+^ 681.1904 (calcd. for C_32_H_32_F_6_O_8_Na, 681.1899).

*S*-MTPA Ester of **5** (5**a**): White amorphous solid. ^1^H NMR (600 MHz, in CD_3_OD) *δ*_H_ 7.41−7.53 (15H, m, MTPA-Ar), 5.41 (1H, br s, H-4), 5.30 (1H, br s, H-7), 5.19 (1H, br s, H-9), 4.13 (1H, d, *J* = 17.5 Hz, H-2a), 3.99 (1H, d, *J* = 17.5 Hz, H-2b), 3.69 (1H, m, H-10), 3.53 (3H, s, MTPA-OMe), 3.52 (3H, s, MTPA-OMe), 3.50 (3H, s, MTPA-OMe), 1.84 (1H, m, H-6a), 1.77 (1H, m, H-5a), 1.73 (1H, m, H-5b), 1.69 (1H, m, H-6b), 1.48 (1H, m, H-11a), 1.41 (1H, m, H-12a), 1.28 (1H, m, H-11b), 1.24 (1H, m, H-12b), 0.86 (3H, t, *J* = 7.1 Hz, H-13); HRESIMS *m/z* [M+Na]^+^ 899.2452 (calcd. for C_42_H_41_F_9_O_10_Na, 899.2454).

*R-*MTPA Ester of **5** (5**b**): White amorphous solid. ^1^H NMR (600 MHz, in CD_3_OD) *δ*_H_ 7.43−7.54 (15H, m, MTPA-Ar), 5.41 (1H, br s, H-4), 5.20 (1H, s, H-7), 4.76 (1H, br s, H-9), 3.95 (1H, d, *J* = 17.6 Hz, H-2a), 3.75 (1H, ddd, *J* = 8.9, 5.0, 2.0 Hz, H-10), 3.68 (1H, dd, *J* = 17.6, 2.0 Hz, H-2b), 3.60 (3H, s, MTPA-OMe), 3.56 (3H, s, MTPA-OMe), 3.48 (3H, s, MTPA-OMe), 2.04 (1H, m, H-5a), 1.99 (1H, m, H-5b), 1.95 (1H, m, H-6a), 1.81 (1H, m, H-6b), 1.32 (1H, m, H-11a), 1.27 (1H, m, H-12a), 1.12 (1H, m, H-12b), 0.95 (1H, m, H-11b), 0.82 (3H, t, *J* = 7.2 Hz, H-13); HRESIMS *m/z* [M+Na]^+^ 899.2449 (calcd. for C_42_H_41_F_9_O_10_Na, 899.2454).

*S*-MTPA Ester of **6** (6**a**): White amorphous solid. ^1^H NMR (600 MHz, in CD_3_OD) *δ*_H_ 7.42−7.52 (15H, m, MTPA-Ar), 5.55 (1H, dd, *J* = 8.0, 6.0 Hz, H-4), 5.38 (1H, br s, H-7), 5.11 (1H, brs, H-9), 3.78 (1H, d, *J* = 16.9 Hz, H-2a), 3.65 (1H, m, H-10), 3.61 (1H, d, *J* = 17.0 Hz, H-2b), 3.54 (3H, s, MTPA-OMe), 3.52 (3H, s, MTPA-OMe), 3.48 (3H, s, MTPA-OMe), 2.11 (1H, m, H-5a), 1.98 (1H, m, H-6a), 1.95 (1H, m, H-6b), 1.61 (1H, m, H-5b), 1.29 (1H, m, H-11a), 1.25 (1H, m, H-12a), 1.20 (1H, m, H-12b), 1.15 (1H, m, H-11b), 0.81 (3H, t, *J* = 7.2 Hz, H-13); HRESIMS *m/z* [M+Na]^+^ 899.2457 (calcd. for C_42_H_41_F_9_O_10_Na, 899.2454).

*R-*MTPA Ester of **6** (6**b**): white amorphous solid. ^1^H NMR (600 MHz, in CD_3_OD) *δ*_H_ 7.45−7.54 (15H, m, MTPA-Ar), 5.52 (1H, dd, *J* = 8.6, 5.3 Hz, H-4), 5.18 (1H, br s, H-7), 4.78 (1H, s, H-9), 4.05 (1H, d, *J* = 17.6 Hz, H-2a), 3.98 (1H, d, *J* = 17.4 Hz, H-2b), 3.84 (1H, m, H-10), 3.59 (3H, s, MTPA-OMe), 3.53 (3H, s, MTPA-OMe), 3.47 (3H, s, MTPA-OMe), 2.12 (1H, m, H-5a), 1.93 (1H, m, H-6a), 1.90 (1H, m, H-6b), 1.68 (1H, m, H-5b), 1.28 (1H, m, H-11a), 1.24 (1H, m, H-12a), 1.14 (1H, m, H-12b), 0.98 (1H, m, H-11b), 0.80 (3H, t, *J* = 7.3 Hz, H-13); HRESIMS *m/z* [M+Na]^+^ 899.2453 (calcd. for C_42_H_41_F_9_O_10_Na, 899.2454).

### 3.7. X-Ray Crystallographic Analysis

Compound **5** was obtained as colorless crystals from acetone–ethyl acetate (1:4) using the vapor-exchanged method [11,19,20]. X-ray single-crystal diffraction data for **5** were collected at 293 K on a Rigaku Oxford Diffraction instrument using Cu Kα (λ = 1.54184) radiation. The structures were solved by direct methods using Olex2 software [21], and the non-hydrogen atoms were refined isotropically with SHELXL-2014 [22] using a full-matrix least squares procedure based on *F^2^*. The hydrogen atoms were positioned according to the geometric calculation. Crystallographic data for the structures of **5** were deposited at the Cambridge Crystallographic Data Centre database (CCDC Number: 1890797).

Crystal data for ***5**:* C_12_H_20_O_4_ (*M* = 228.28 g/mol), trigonal, space group P3_1_ (no. 144), *a* = 14.9243(2)Å, *b* = 14.9243(2)Å, *c* = 4.49716(13)Å, *V* = 867.47(4)Å^3^, *Z* = 3, *T* = 293 (10)K, μ(CuKα) = 0.798 mm^−1^, *Dcalc* = 1.311 g/cm^3^, F (000) = 372.0. The final *R*_1_ was 0.0397 (*wR*_2_ = 0.1052) for 2082 unique reflections (I > 2σ (I)), and Flack = −0.01(11).

### 3.8. NO Inhibition Assay

Mouse monocyte-macrophages RAW 264.7 (ATCC TIB-71) were purchased from the Chinese Academy of Science. DMEM medium, penicillin, streptomycin, and fetal bovine serum were purchased from Invitrogen (N.Y., USA). Lipopolysaccharide (LPS), DMSO, 3-(4,5-dimethylthiazol-2-yl)-2,5-diphenyl-2H-tetrazolium bromide (MTT), and hydrocortisone were obtained from Sigma Co. RAW 264.7 cells were maintained in DMEM medium supplemented with penicillin, streptomycin (both 100 U/mL), and 10% heat-inactivated fetal bovine serum at 37 °C in a humidified incubator with 5% CO_2_ and 95% air. RAW264.7 cells were passaged until they attained confluence. The cell concentration was adjusted to 5 × 10^5^ cells/mL with 10% FBS DMEM medium, and 100 μL was seeded in each well of a 96-well plate that was maintained at 37 °C overnight. The supernatant was discarded, and the cells except for the blank control groups were treated with 100 μL of LPS DMEM (the concentration of LPS was 2 μg/mL or 5 μg/mL). After 2 h of incubation, blank and LPS model groups were treated with 100 μL of DMEM medium and the others were treated with DMEM medium containing various concentrations of test compounds for 24 h, respectively [16]. 

As a parameter of NO release, the nitrite concentration was measured in the supernatant of RAW 264.7 cells by the Griess reaction [16]. The inhibition rate was calculated and plotted versus test concentrations to afford the IC_50_.

### 3.9. Cytotoxic Assay

The cytotoxicity of all of the compounds was measured by the MTT assay against the prostate cancer PC3 cell line. Tumor cell lines were seeded in 96-well plates (4000 per well in 100 μL). After 24 h of incubation, cells were treated with gradient concentrations (100 μM, 50 μM, 25 μM, 12.5 μM, 6.25 μM, 3.125 μM) for another 72 h. Afterwards, MTT solution (5.0 mg/mL in RPMI-1640 media, Sigma, St. Louis, MO, USA) was added (20 μL/well) and then plates were incubated for another 4 h at 37 °C. The compounds were dissolved in DMSO and a cell growth inhibition assay was performed as reported previously. The growth inhibitory abilities of the compounds were calculated and expressed using the IC_50_ value by the Dose-Effect Analysis software. Doxorubicin (ADR) was used as a positive control.

### 3.10. Antibacterial Assay

The antibacterial activities of all compounds except for **4** and **7** were evaluated with *S. aureus*, *S. epidermidis*, *E. coli*, *M. albicans*, and *P. aeruginosa* using the Kirby−Bauer Diffusion method. Gentamicin, vancomycin, ampicillin, amphotericin B, and gentamicin served as the positive drugs, respectively.

The stock solution of compounds **1**, **2**, **3**, **5**, and **8** was prepared in methanol with a series of concentrations (25, 5.0, 1, and 0.2 mg/mL). The stock solution of compound **6** was prepared in methanol with a series of concentrations (5.0, 1, and 0.2 mg/mL). The positive control drugs gentamicin, vancomycin, and ampicillin were dissolved in purified water at a concentration of 500 ug/mL. Amphotericin B was dissolved in DMSO at a concentration of 100 ug/mL.

*S. aureus*, *S. epidermidis*, *E. coli*, *M. albicans*, and *P. aeruginosa* were activated in nutrient broth at 37 °C for 6~8 h with 200 rpm. The culture broth was poured on the surface of Malt Extract Broth (MEB) Agar (20 g MEB and 20 g agar in 1 L of water) plates inside the cabinet, making sure that there was a uniform distribution of cells on the plates. The location of the negative control (methanol), the positive drugs, and different contents of tested compounds (100 µg, 20 µg, 4 µg, and 0.8 µg for **1**, **2**, **3**, **5**, and **8**; 20 µg, 4 µg, and 0.8 µg for **6**) was labeled on the agar side of the plate and outside the lid. The discs were arranged so as to mimic their positions outside the lid and 4 µL of the corresponding concentrations of the tested compounds and methanol was added to the discs. The contents of the positive drugs gentamicin, ampicillin, and amphotericin B were 3 ug, 1.5 ug, and 0.35 ug, respectively. The discs were transferred from the lid to the agar plate using sterile forceps. Finally, the plates were placed in an incubator agar side up at 37 °C for 12 h and the inhibition zone was observed.

The estimations of the purity of compounds **1**–**8** for biological appraisals were 92.21%, 98.26%, 95.82%, 98.60%, 99.1%, 95.36%, and 99.05%, respectively.

## 4. Conclusions

With the aim of discovering structurally novel secondary metabolites, seven new polyketides (phomopsiketones D–G (**1**–**4**) and letendronols A–C (**5**–**7**)) were isolated from the marine endophytic fungus *Letendraea* sp. in 1/2 PDB medium. The structures and absolute configurations of the new compounds were sufficiently elucidated through HRESIMS, NMR, single-crystal X-ray diffraction, ECD calculation, and a modified version of Mosher’s method. Compound **2** showed weak anti-inflammatory activity.

## Figures and Tables

**Table 1:** NMR data for compounds **1**–**7.**

No.	1 ^a^	2 ^a^	3 ^b^	4 ^c^	5 ^b^	6 ^b^	7 ^b^
	*δ*_H_ (*J* in Hz)	*δ*_H_ (*J* in Hz)	*δ*_H_ (*J* in Hz)	*δ*_H_ (*J* in Hz)	*δ*_H_ (*J* in Hz)	*δ*_H_ (*J* in Hz)	*δ*_H_ (*J* in Hz)
2	5.29, s	5.48, s	4.69, ddd (12.5, 3.1, 2.2) 4.74, ddd (12.0, 5.5, 3.0)	4.76, m 4.97, m	4.02, overlapped 4.30, dt (16.5, 2.3)	4.00, overlapped 4.37, overlapped	4.60, d (15.6) 4.82, d (15.6)
4	4.59, br s	4.63, d (9.2)			4.06, m	3.95, dd (4.5, 5.6)	
5	2.06, m 2.28, m	2.01, m 2.30, m	2.48, m 2.52, m	2.47, m 2.53, dt (16.0, 4.7)	1.54, m 2.02, m	1.74, m 1.79, m	6.63, d (8.0)
6	2.40, m 2.70, ddd (17.0, 7.0, 5.0)	2.40, m 2.65, dt (17.0, 4.0)	2.01, m 2.34, ddd (12.6, 9.4, 4.7)	2.01, m 2.31, ddd (12.8, 9.4, 4.8)	1.56, m 2.07, m	1.72, m 1.82, m	7.07, t (8.0)
7			4.67, m	4.64, m	4.43, m	4.35, overlapped	7.01, d (8.0)
9	1.88, m 2.36, m	1.84, m 2.36, m	5.10, m	4.94, m	3.99, overlapped	3.98, overlapped	4.30, d (8.5)
10	3.74, m	3.70, m	3.80, dt (8.0, 4.0)	3.83, dt (9.5, 3.2)	3.33, overlapped	3.30, overlapped	3.37, m
11	1.55, m 1.61, m	1.54, m 1.59, m	1.52, m 1.54, m	1.39, m 1.44, m	1.46, m 1.75, m	1.48, m 1.72, m	1.50, m 1.89, m
12	1.43, m 1.52, m	1.43, m 1.50, m	1.58, m 1.42, m	1.53, m 1.33, m	1.41, m 1.59, m	1.40, m 1.56, m	1.48, m 1.65, m
13	0.95, t (7.0)	0.94, t (7.0)	0.96, t (7.0)	0.96, t (7.0)	0.96, t (7.2)	0.96, t (7.3)	0.99, t (7.2)
15	3.50, s	3.86, ddd (12.1,8.8,2.8) 3.97, dt (12.0, 3.0)					
16		3.60, dt (11.0, 3.0) 3.66, m					
18		3.53, m 3.53, m					
19		1.61, m 1.63, m					
20		1.36, m 1.39, m					
21		0.94, t (7.0)					

^a^ Measured in CDCl_3_ and at 500 MHz NMR. ^b^ Measured in CD_3_OD and at 500 MHz NMR. ^c^ Measured in CD_3_OD and at 600 MHz NMR.

**Table 2:** ^13^C NMR data for compounds **1**–**7.**

No.	1 ^a^	2 ^a^	3 ^b^	4 ^c^	5 ^b^	6 ^b^	7 ^b^
	*δ*_C,_ Type	*δ*_C,_ Type	*δ*_C,_ Type	*δ*_C,_ Type	*δ*_C,_ Type	*δ*_C,_ Type	*δ*_C,_ Type
2	95.2, CH	95.6, CH	73.8, CH_2_	76.0, CH_2_	67.1, CH_2_	65.6, CH_2_	65.3, CH_2_
3	151.4, C	154.4, C	136.0, C	134.6, C	137.1, C	137.0, C	123.3, C
4	65.3, CH	66.5, CH	197.1, C	197.1, C	67.2, CH	66.1, CH	153.5, C
5	31.3, CH_2_	30.9, CH_2_	37.1, CH_2_	37.6, CH_2_	29.6, CH_2_	29.1, CH_2_	113.8, CH
6	34.4, CH_2_	36.1, CH_2_	34.5, CH_2_	34.1, CH_2_	29.8, CH_2_	29.2, CH_2_	128.3, CH
7	198.2, C	197.9, C	66.0, CH	65.7, CH	64.2, CH	64.4, CH	118.9, CH
8	132.5, C	131.5, C	163.6, C	167.0, C	135.5, C	135.3, C	140.0, C
9	27.4, CH_2_	27.2, CH_2_	91.3, CH	90.0, CH	67.0, CH	67.0, CH	70.1, CH
10	66.7, CH	67.4, CH	74.4, CH	74.0, CH	80.4, CH	80.2, CH	80.4, CH
11	37.4, CH_2_	37.2, CH_2_	35.6, CH_2_	34.1, CH_2_	35.2, CH_2_	35.0, CH_2_	35.5, CH_2_
12	18.9, CH_2_	18.8, CH_2_	20.0, CH_2_	20.1, CH_2_	20.0, CH_2_	19.9, CH_2_	19.8, CH_2_
13	14.1, CH_3_	14.0, CH_3_	14.4, CH_3_	14.4, CH_3_	14.4, CH_3_	14.4, CH_3_	14.4, CH_3_
15	55.8, CH_3_	68.4, CH_2_					
16		69.9, CH_2_					
18		71.5, CH_2_					
19		31.5, CH_2_					
20		19.3, CH_2_					
21		14.0, CH_3_					

^a^ Measured in CDCl_3_ and at 125 MHz NMR. ^b^ Measured in CD_3_OD and at 125 MHz NMR. ^c^ Measured in CD_3_OD and at 150 MHz NMR.

## Supplementary Materials

The following are available online at https://www.mdpi.com/1660-3397/18/1/18/s1, Tables S1–S7: 1D and 2D NMR data for **1**–**7**, Figures S1–S4: ^1^H-^1^H COSY and key HMBC correlations of **2**, **4**, **5**, **6**, and **7**, Figure S5: NOESY correlation of 7, 10-*di*-*R*-MTPA ester (**3f**), Figures S6 and S7: Δδ *S*-*R* values for the MTPA esters (**4a** and **4b**, **5a** and **5b**, **6a** and **6b**), Figure S8: comparison of CD spectra of **3** and **4**, Figures S9–S81: 1D and 2D NMR, HRESIMS, IR, and CD spectra for the compounds (**1**–**7**), Figures S82–S83: ^1^H NMR and 13C NMR spectra of **8**, Figures S84, S86, and S87: ^1^H NMR and HRESIMS of **3a** and **3b**, Figure S85: ^1^H–^1^H COSY spectrum of **3a**, Figures S88–S90: ^1^H NMR and HRESIMS of **3c** and **3d**, Figures S91, S93, and S94: ^1^H NMR and HRESIMS of **3e** and **3f**, Figure S92: NOESY spectrum of **3f**, Figures S95–S97: ^1^H NMR and HRESIMS data for **4a** and **4b**, Figures S98–S109: 1D and 2D NMR and HRESIMS data for **5a** and **5b**, Figure S110–S112: ^1^H NMR and HRESIMS data for **6a** and **6b**. The 26s rDNA sequence information of *Letendraea* sp. Crystallographic data of **5** (CIF).
