# Supplementary material for: New Polyketides from the Marine-Derived Fungus Letendraea Sp. 5XNZ4-2
Source: Mar Drugs. 2019 Dec 24;18(1):18. doi: 10.3390/md18010018 (PMC7024145; doi:10.3390/md18010018)
Supplement: Supplementary file 1 [file marinedrugs-18-00018-s001.pdf]

Supplementary material

New Polyketides from the Marine-derived Fungus *Letendraea* sp. 5XNZ4-2

Yan Xu [1], Ruibao Huang [1], Hongwei Liu [2], Tingting Yan [1], Wanjing Ding [1], Yongjun Jiang [1], Pinmei Wang [1], Daoqiong Zheng [1], and Jinzhong Xu [1,*]

[1] Ocean College, Zhoushan Campus, Zhejiang University, Zhoushan 316021, China; xuyan875@126.com (Y.X.); fau_rhuang@fau.edu (R.H.), ytt706@126.com(T.Y.), wading@zju.edu.cn(W.D.); yongjunjiang89@hotmail.com (Y.J.); wangpinmei@zju.edu.cn (P.W.); zhengdaoqiong@zju.edu.cn (D.Z.)

[2] State Key Laboratory of Mycology, Institute of Microbiology, Chinese Academy of Sciences, Beijing 100101, China; liuhw@im.ac.cn(H.L.)

* Correspondence: xujinzhong@zju.edu.cn; Tel.: 86-158-5816-8018

List of Supplementary material

Table S1. 1D and 2D NMR data of 1 (in $CDCl_3$)........5
Table S2. 1D and 2D NMR data of 2 (in $CDCl_3$)........6
Table S3. 1D and 2D NMR data of 3 (in CD_3OD)........7
Table S4. 1D and 2D NMR data of 4 (in $CDCl_3$)........8
Table S5. 1D and 2D NMR data of 5 (in CD_3OD)........9
Table S6. 1D and 2D NMR data of 6 (in CD_3OD)........10
Table S7. 1D and 2D NMR data of 7 (in CD_3OD)........11
Figure S1. ^1H-^1H COSY and key HMBC correlations of 2........12
Figure S2. ^1H-^1H COSY and key HMBC correlations of 4........12
Figure S3. ^1H-^1H COSY and key HMBC correlations of 5 and 6........13
Figure S4. ^1H-^1H COSY and key HMBC correlations of 7........13
Figure S5. NOESY correlation of 7, 10-*di-R*-MTPA ester (3f)........13
Figure S6. $\Delta\delta_{S-R}$ values for the MTPA esters (4a and 4b)........14
Figure S7. $\Delta\delta_{S-R}$ values for the MTPA esters (5a and 5b, 6a and 6b)........14
Figure S8. Comparison of CD spectra of 3 and 4........14
Figure S9. ^1H NMR (500 MHz, $CDCl_3$) spectrum of phomopsiketone D (1)........15
Figure S10. ^{13}C NMR (125 MHz, $CDCl_3$) spectrum of phomopsiketone D (1)........16
Figure S11. DEPT ($CDCl_3$) spectrum of phomopsiketone D (1)........17
Figure S12. HSQC ($CDCl_3$) spectrum of phomopsiketone D (1)........18
Figure S13. ^1H–^1H COSY ($CDCl_3$) spectrum of phomopsiketone D (1)........19
Figure S14. HMBC ($CDCl_3$) spectrum of phomopsiketone D (1)........20
Figure S15. NOESY ($CDCl_3$) spectrum of phomopsiketone D (1)........21
Figure S16. HRESIMS spectrum of phomopsiketone D (1)........22
Figure S17. IR spectrum of phomopsiketone D (1)........22
Figure S18. CD spectrum of phomopsiketone D (1)........23
Figure S19. ^1H NMR (500 MHz, $CDCl_3$) spectrum of phomopsiketone E (2)........23

Figure S20. ^{13}C NMR (125 MHz, $CDCl_3$) spectrum of phomopsiketone E (2) 24
Figure S21. DEPT ($CDCl_3$) spectrum of phomopsiketone E (2) 25
Figure S22. HSQC ($CDCl_3$) spectrum of phomopsiketone E (2) 26
Figure S23. ^1H–^1H COSY ($CDCl_3$) spectrum of phomopsiketone E (2) 27
Figure S24. HMBC ($CDCl_3$) spectrum of phomopsiketone E (2) 28
Figure S25. NOESY ($CDCl_3$) spectrum of phomopsiketone E (2) 29
Figure S26. HRESIMS spectrum of phomopsiketone E (2) 29
Figure S27. IR spectrum of phomopsiketone E (2) 30
Figure S28. CD spectrum of phomopsiketone E (2) 30
Figure S29. ^1H NMR (500 MHz, CD_3OD) spectrum of phomopsiketone F (3) 31
Figure S30. ^{13}C NMR (125 MHz, CD_3OD) spectrum of phomopsiketone F (3) 32
Figure S31. HSQC (CD_3OD) spectrum of phomopsiketone F (3) 33
Figure S32. ^1H–^1H COSY (CD_3OD) spectrum of phomopsiketone F (3) 34
Figure S33. HMBC (CD_3OD) spectrum of phomopsiketone F (3) 35
Figure S34. NOESY (CD_3OD) spectrum of phomopsiketone F (3) 36
Figure S35. HRESIMS spectrum of phomopsiketone F (3) 36
Figure S36. IR spectrum of phomopsiketone F (3) 37
Figure S37. CD spectrum of phomopsiketone F (3) 37
Figure S38. ^1H NMR (600 MHz, CD_3OD) spectrum of phomopsiketone G (4) 38
Figure S39. ^{13}C NMR (150 MHz, CD_3OD) spectrum of phomopsiketone G (4) 39
Figure S40. HSQC (CD_3OD) spectrum of phomopsiketone G (4) 40
Figure S41. ^1H–^1H COSY (CD_3OD) spectrum of phomopsiketone G (4) 41
Figure S42. HMBC (CD_3OD) spectrum of phomopsiketone G (4) 42
Figure S43. NOESY (CD_3OD) spectrum of phomopsiketone G (4) 43
Figure S44. ^1H NMR (500 MHz, $CDCl_3$) spectrum of phomopsiketone G (4) 44
Figure S45. ^{13}C NMR (125 MHz, $CDCl_3$) spectrum of phomopsiketone G (4) 45
Figure S46. DEPT (CDCl3) spectrum of phomopsiketone G (4) 46
Figure S47. HSQC (CD_3OD) spectrum of phomopsiketone G (4) 47
Figure S48. ^1H–^1H COSY ($CDCl_3$) spectrum of phomopsiketone G (4) 48
Figure S49. HMBC ($CDCl_3$) spectrum of phomopsiketone G (4) 49
Figure S50. HRESIMS spectrum of phomopsiketone G (4) 49
Figure S51. IR spectrum of phomopsiketone G (4) 50
Figure S52. CD spectrum of phomopsiketone G (4) 50
Figure S53. ^1H NMR (500 MHz, CD_3OD) spectrum of letendronol A (5) 51
Figure S54. ^{13}C NMR (125 MHz, CD_3OD) spectrum of letendronol A (5) 52
Figure S55. DEPT (CD_3OD) spectrum of letendronol A (5) 53
Figure S56. HSQC (CD_3OD) spectrum of letendronol A (5) 54
Figure S57. ^1H–^1H COSY (CD_3OD) spectrum of letendronol A (5) 55
Figure S58. HMBC (CD_3OD) spectrum of letendronol A (5) 56
Figure S59. NOESY (CD_3OD) spectrum of letendronol A (5) 57
Figure S60. HRESIMS spectrum of letendronol A (5) 57
Figure S61. IR spectrum of letendronol A (5) 58
Figure S62. CD spectrum of letendronol A (5) 58
Figure S63. ^1H NMR (500 MHz, CD_3OD) spectrum of letendronol B (6) 59

Figure S64. ^{13}C NMR (125 MHz, CD_3OD) spectrum of letendronol B (6)....60
Figure S65. DEPT (CD_3OD) spectrum of letendronol B (6)....61
Figure S66. HSQC (CD_3OD) spectrum of letendronol B (6)....62
Figure S67. ^{1}H–^{1}H COSY (CD_3OD) spectrum of letendronol B (6)....63
Figure S68. HMBC (CD_3OD) spectrum of letendronol B (6)....64
Figure S69. NOESY (CD_3OD) spectrum of letendronol B (6)....65
Figure S70. HRESIMS spectrum of letendronol B (6)....65
Figure S71. IR spectrum of letendronol B (6)....66
Figure S72. CD spectrum of letendronol B (6)....66
Figure S73. ^{1}H NMR (500 MHz, CD_3OD) spectrum of letendronol C (7)....67
Figure S74. ^{13}C NMR (125 MHz, CD_3OD) spectrum of letendronol C (7)....68
Figure S75. HSQC (CD_3OD) spectrum of letendronol C (7)....69
Figure S76. ^{1}H–^{1}H COSY (CD_3OD) spectrum of letendronol C (7)....70
Figure S77. HMBC (CD_3OD) spectrum of letendronol C (7)....71
Figure S78. NOESY (CD_3OD) spectrum of letendronol C (7)....72
Figure S79. HRESIMS spectrum of letendronol C (7)....72
Figure S80. IR spectrum of letendronol C (7)....73
Figure S81. CD spectrum of letendronol C (7)....73
Figure S82. ^{1}H NMR (500 MHz, CD_3OD) spectrum of xylarinol B (8)....74
Figure S83. ^{13}C NMR (125 MHz, CD_3OD) spectrum of xylarinol B (8)....75
Figure S84. ^{1}H NMR (600 MHz, CD_3OD) spectrum of 3a and 3b....76
Figure S85. ^{1}H–^{1}H COSY (CD_3OD) spectrum of 3a....77
Figure S86. HRESIMS spectrum of 3a....78
Figure S87. HRESIMS spectrum of 3b....78
Figure S88. ^{1}H NMR (600 MHz, CD_3OD) spectrum of 3c and 3d....79
Figure S89. HRESIMS spectrum of 3c....80
Figure S90. HRESIMS spectrum of 3d....80
Figure S91. ^{1}H NMR (600 MHz, CD_3OD) spectrum of 3e and 3f....81
Figure S92. NOESY (CD_3OD) spectrum of 3f....82
Figure S93. HRESIMS spectrum of 3e....83
Figure S94. HRESIMS spectrum of 3f....83
Figure S95. ^{1}H NMR (600 MHz, CD_3OD) spectrum of 4a and 4b....84
Figure S96. HRESIMS spectrum of 4a....85
Figure S97. HRESIMS spectrum of 4b....85
Figure S98. ^{1}H NMR (600 MHz, CD_3OD) spectrum of 5a and 5b....86
Figure S99. ^{13}C NMR (150 MHz, CD_3OD) spectrum of 5a....87
Figure S100. HSQC (CD_3OD) spectrum of 5a....88
Figure S101. COSY (CD_3OD) spectrum of 5a....89
Figure S102. HMBC (CD_3OD) spectrum of 5a....90
Figure S103. NOESY (CD_3OD) spectrum of 5a....91
Figure S104. ^{13}C NMR (150 MHz, CD_3OD) spectrum of 5b....92
Figure S105. HSQC (CD_3OD) spectrum of 5b....93
Figure S106. COSY (CD_3OD) spectrum of 5b....94
Figure S107. HMBC (CD_3OD) spectrum of 5b....95

Figure S108. HRESIMS spectrum of 5a 96
Figure S109. HRESIMS spectrum of 5b 96
Figure S110. ^{1}H NMR (600 MHz, CD_3OD) spectrum of 6a and 6b 97
Figure S111. HRESIMS spectrum of 6a 98
Figure S112. HRESIMS spectrum of 6b 98
The 26s rDNA sequence information of *Letendraea* sp. 99

Table S1. 1D and 2D NMR data of **1** (in $CDCl_3$)

No.	δ_H (*J* in Hz)	$\delta_{C,}$ type	^1H-^1H COSY	HMBC H# — C#	NOESY H# — H#
2	5.29, s	95.2, CH		C-3, 4, 8, 10	4, 10
3		151.4, C			
4	4.59, br s	65.3, CH	5a, 5b		2, 6a
5a	2.06, m	31.3, CH_2	4, 6a, 6b	C-3, 4, 6, 7	
5b	2.28, m			C-3, 4, 6, 7	
6a	2.40, m	34.4, CH_2	5a	C-3, 4, 5, 7, 8	4
6b	2.70, ddd (17.0, 7.0, 5.0)		5a, 5b	C-4, 5, 7, 8	
7		198.2, C			
8		132.5, C			
9a	1.88, m	27.4, CH_2	10	C-3, 7, 8, 10, C-11	
9b	2.36, m		10	C-3, 7, 8	
10	3.74, m	66.7, CH	9a, 9b, 11a, 11b	C-2, 8, 11, 12	
11a	1.55, m	37.4, CH_2	10	C-9, 10, 12, 13	
11b	1.61, m		10	C-9, 10, 12, 13	
12a	1.43, m	18.9, CH_2	13	C-10, 11, 13	
12b	1.52, m		13	C-10, 11, 13	
13	0.95, t (7.0)	14.1, CH_3	12a, 12b	C-10, 11, 12	
15	3.50, s	55.8, CH_3		C-2	

Table S2. 1D and 2D NMR data of **2** (in $CDCl_3$)

No.	δ_H (*J* in Hz)	δ_C, type	^1H-^1H COSY	HMBC<br>H# − C#	NOESY<br>H# − H#
2	5.48, s	95.6, CH		C-3, 8, 10	4, 10
3		154.4, C			
4	4.63, d (9.2)	66.5, CH	5a, 5b		2, 6a
5a	2.01, m	30.9, CH_2	4, 6a, 6b	C-3, 4, 6, 7	
5b	2.30, m		4, 6b	C-2, 3, 4, 6, 7	
6a	2.40, m	36.1, CH_2	5a	C-4, 5, 7, 8	
6b	2.65, dt (17.0, 4.0)		5a, 5b	C-4, 5, 7, 8	
7		197.9, C			
8		131.5, C			
9a	1.84, m	27.2, CH_2	10	C-3, 8, 10, 11	
9b	2.36, m		10	C-3, 8	
10	3.70, m	67.4, CH	9a, 9b, 11a, 11b	C-2, 8, 11, 12	2
11a	1.54, m	37.2, CH_2	10	C-9, 10, 12, 13	
11b	1.59, m		10	C-9, 10, 12, 13	
12a	1.43, m	18.8, CH_2	13	C-10, 11, 13	
12b	1.50, m		13	C-10, 11, 13	
13	0.94, t (7.0)	14.0, CH_3	12a, 12b	C-11, 12	
15a	3.86, ddd (12.1, 8.8,2.8)	68.4, CH_2	16a, 16b	C-2, 16	
15b	3.97,dt (12.0, 3.0)		16a, 16b	C-2, 16	
16a	3.60, dt (11.0, 3.0)	69.9, CH_2	15a, 15b	C-15, 18	
16b	3.66, m		15a, 15b	C-15, 18	
18	3.53, 2H, m	71.5, CH_2	19a, 19b	C-16, 19, 20	
19a	1.61, m	31.5, CH_2	18, 20a, 20b	C-18, 20, 21	
19b	1.63, m		18, 20a, 20b	C-18, 20, 21	
20a	1.36, m	19.3, CH_2	19a, 19b, 21	C-18, 19, 21	
20b	1.39, m		19a, 19b, 21	C-18, 19, 21	
21	0.94, t (7.0)	14.0, CH_3	20a, 20b	C-19, 20	

Table S3. 1D and 2D NMR data of **3** (in CD_3OD)

No.	δ_H (J in Hz)	$\delta_{C,}$ type	^1H-^1H COSY	HMBC<br>H# — C#	NOESY<br>H# — H#
2a	4.69, ddd (12.5, 3.1, 2.2)	73.8, CH_2		C-3, 7, 9, 10	
2b	4.74, ddd (12.0, 5.5, 3.0)			C-3, 7, 9, 10	
3		136.0, C			
4		197.1, C			
5a	2.48, m	37.1, CH_2	6a, 6b	C-3, 4, 6, 7	
5b	2.52, m		6a, 6b	C-3, 4, 6, 7	
6a	2.01, m	34.5, CH_2	5a, 5b, 7	C-4, 5, 7, 8	
6b	2.34, ddd (12.6, 9.4, 4.7)		5a, 5b, 7	C-4, 5, 7, 8, 9	
7	4.67, m	66.0, CH	6a, 6b	C-3, 8, 9	
8		163.6, C			
9	5.10, m	91.3, CH	10	C-2, 3, 8, 10, 11	
10	3.80, dt (8.0, 4.0)	74.4, CH	9, 11a, 11b	C-8, 9, 11, 12	
11a	1.52, m	35.6, CH_2	10, 12a, 12b	C-9, 10, 12, 13	
11b	1.54, m		10, 12a, 12b	C-9, 10, 12, 13	
12a	1.58, m	20.0, CH_2	11a, 11b, 13	C-10, 11, 13	
12b	1.42, m		11a, 11b, 13	C-10, 11, 13	
13	0.96, t (7.0)	14.4, CH_3	12a, 12b	C-11, 12	

Table S4. 1D and 2D NMR data of **4** (in $CDCl_3$)

No.	δ_H (*J* in Hz)	δ_C, type	^{1}H-^{1}H COSY	HMBC H# — C#	NOESY H# — H#
2a	4.77, m	74.6, CH_2		C-3, 8, 10	
2b	5.02, dd (16.0, 5.1)			C-3, 8, 9, 10	
3		135.5, C			
4		196.1, C			
5a	2.50, m	36.8, CH_2	6a	C-4, 6, 7	
5b	2.67, dt (17.0, 5.0)		6a	C-3, 4, 6, 7	
6a	2.11, m	33.5, CH_2	5a, 5b, 7	C-4, 5, 7, 8	
6b	2.40, m		7	C-4, 5, 7, 8	
7	4.70, br s	65.4, CH	6a, 6b		
8		164.3, C			
9	4.81, m	88.4, CH	10	C-3, 8, 10	
10	3.69, m	73.5, CH	9, 11a, 11b		
11a	1.45, m	34.8, CH_2	10	C-10, 12, 13	
11b	1.60, m			C-12, 13	
12a	1.42, m	18.6, CH_2	13	C-10, 11, 13	
12b	1.58, m		13	C-11, 13	
13	0.93, t (7.0)	14.3, CH_3	12a, 12b	C-11, 12	

Table S5. 1D and 2D NMR data of **5** (in CD_3OD)

No.	δ_H (*J* in Hz)	δ_C, type	^{1}H-^{1}H COSY	HMBC H# − C#	NOESY H# − H#
2a	4.02, overlapped	67.1, CH_2		C-3, 4, 8, 10	
2b	4.30, dt (16.5, 2.3)			C-3, 4, 7, 8, 10	10
3		137.1, C			
4	4.06, m	67.2, CH	5a, 5b	C-3, 5, 6, 8	
5a	1.54, m	29.6, CH_2	4, 6a, 6b	C-3, 4, 6, 7	
5b	2.02, m		4, 6a, 6b	C-3, 4, 6, 7	
6a	1.56, m	29.8, CH_2	7, 5a, 5b	C-4, 5, 7, 8	
6b	2.07, m		7, 5a, 5b	C-4, 5, 7, 8	
7	4.43, m	64.2, CH	6a, 6b	C-3, 5, 6, 8, 9	
8		135.5, C			
9	3.99, overlapped	67.0, CH	10	C-3, 4, 8, 10, 11	
10	3.33, overlapped	80.4, CH	9, 11a, 11b	C-2, 8, 9, 11, 12	2b
11a	1.46, m	35.2, CH_2	10, 12a, 12b	C-9, 10, 12, 13	
11b	1.75, m		10, 12a, 12b	C-9, 10, 12, 13	
12a	1.41, m	20.0, CH_2	11a, 11b, 13	C-10, 11, 13	
12b	1.59, m		11a, 11b, 13	C-10, 11, 13	
13	0.96, t (7.2)	14.4, CH_3	12a, 12b	C-11, 12	

Table S6. 1D and 2D NMR data of **6** (in CD_3OD)

No.	δ_H (*J* in Hz)	δ_C, type	^1H-^1H COSY	HMBC H# — C#	NOESY H# — H#
2a	4.00 , overlapped	65.6, CH_2		C-3, 4, 8, 10	
2b	4.37 , overlapped			C-3, 4, 8, 10	
3		137.0, C			
4	3.95, dd (4.5, 5.6)	66.1, CH	5a, 5b	C-3, 5, 6, 8	
5a	1.74, m	29.1, CH_2	4	C-3, 4, 6, 7	
5b	1.79, m		4	C-3, 4, 6, 7	
6a	1.72, m	29.2, CH_2		C-4, 5, 7, 8	
6b	1.82, m		7	C-4, 5, 7, 8	
7	4.35, overlapped	64.4, CH	6b	C-3, 5, 6, 8	
8		135.3, C			
9	3.98, overlapped	67.0, CH	10	C-3, 7, 8, 10, 11	
10	3.30, overlapped	80.2, CH	9, 11a, 11b	C-8, 9, 11, 12	
11a	1.48, m	35.0, CH_2	10	C-9, 10, 12, 13	
11b	1.72, m		10	C-9, 10, 12, 13	
12a	1.40, m	19.9, CH_2	13	C-10, 12, 13	
12b	1.56, m		13	C-10, 12, 13	
13	0.96, t (7.3)	14.4, CH_3	12a, 12b	C-11, 12	

Table S7. 1D and 2D NMR data of **7** (in CD_3OD)

No.	δ_H (*J* in Hz)	δ_C, type	^1H-^1H COSY	HMBC H# — C#	NOESY H# — H#
2a	4.60, d (15.6)	65.3, CH_2		C-3, 4, 8, 10	10
2b	4.82, d (15.6)			C-3, 4, 8, 10	
3		123.3, C			
4		153.5, C			
5	6.63, d (8.0)	113.8, CH	6	C-3, 4, 7	
6	7.07, t (8.0)	128.3, CH	5	C-4, 5, 8	
7	7.01, d (8.0)	118.9, CH		C-3, 5, 6, 9	
8		140.0, C			
9	4.30, d (8.5)	70.1, CH	10	C-3, 7, 8, 10, 11	
10	3.37, m	80.4, CH	9, 11a, 11b	C-9, 11, 12	2a
11a	1.50, m	35.5, CH_2	10, 12a, 12b	C-10, 12, 13	
11b	1.89, m		10, 12a, 12b	C-10, 12, 13	
12a	1.48, m	19.8, CH_2	11a, 11b, 13	C-11, 13	
12b	1.65, m		11a, 11b, 13	C-11, 13	
13	0.99, t (7.2)	14.4, CH_3	12a, 12b	C-11, 12	

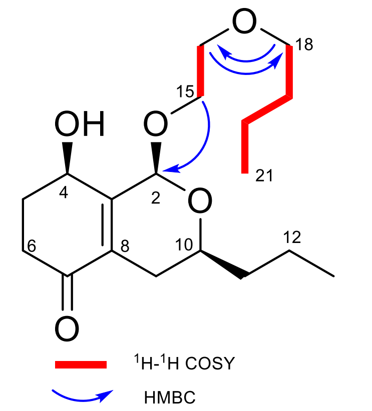

Figure S1. ^{1}H-^{1}H COSY and key HMBC correlations of **2**

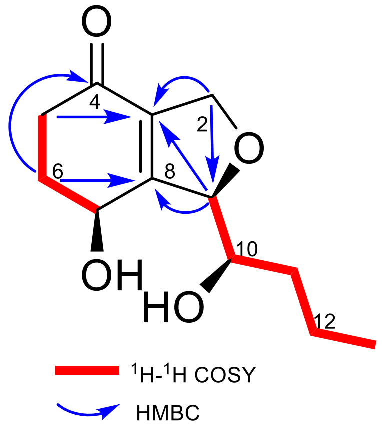

Figure S2. ^{1}H-^{1}H COSY and key HMBC correlations of **4**

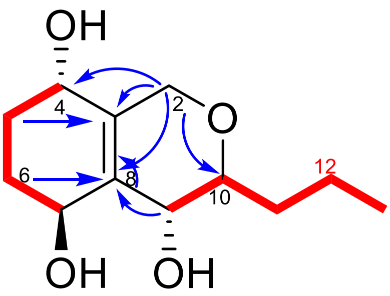

5

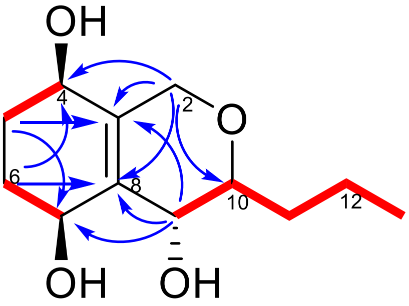

6

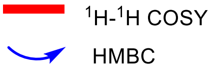

Figure S3. ^{1}H-^{1}H COSY and key HMBC correlations of **5** and **6**

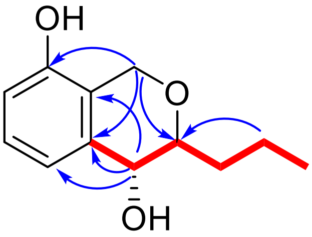

7

Figure S4. ^{1}H-^{1}H COSY and key HMBC correlations of **7**

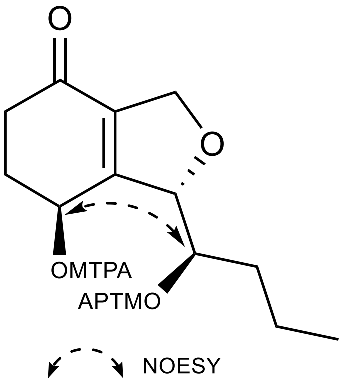

Figure S5. NOESY correlation of 7, 10-*di*-*R*-MTPA ester (**3f**)

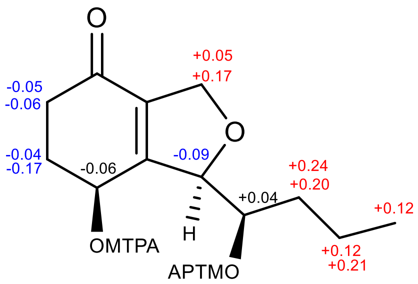

4a = *S*-MTPA
4b = *R*-MTPA

Figure S6. Δδ *S-R* values for the MTPA esters (**4a** and **4b**).

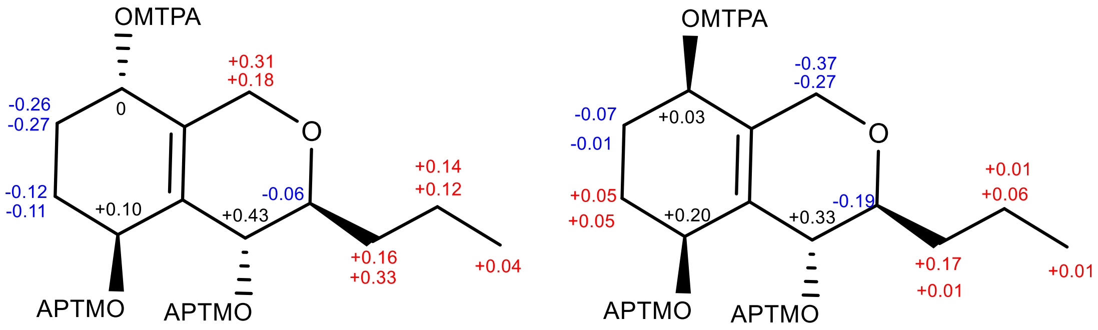

5a = *S*-MTPA
5b = *R*-MTPA

6a = *S*-MTPA
6b = *R*-MTPA

Figure S7. Δδ *S-R* values for the MTPA esters (**5a** and **5b**, **6a** and **6b**)

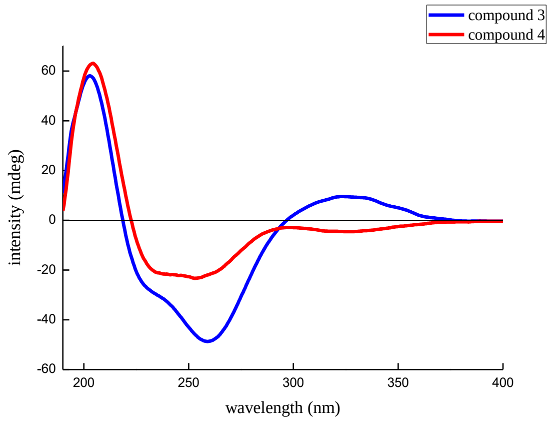

Figure S8. Comparison of CD spectra of **3** and **4**

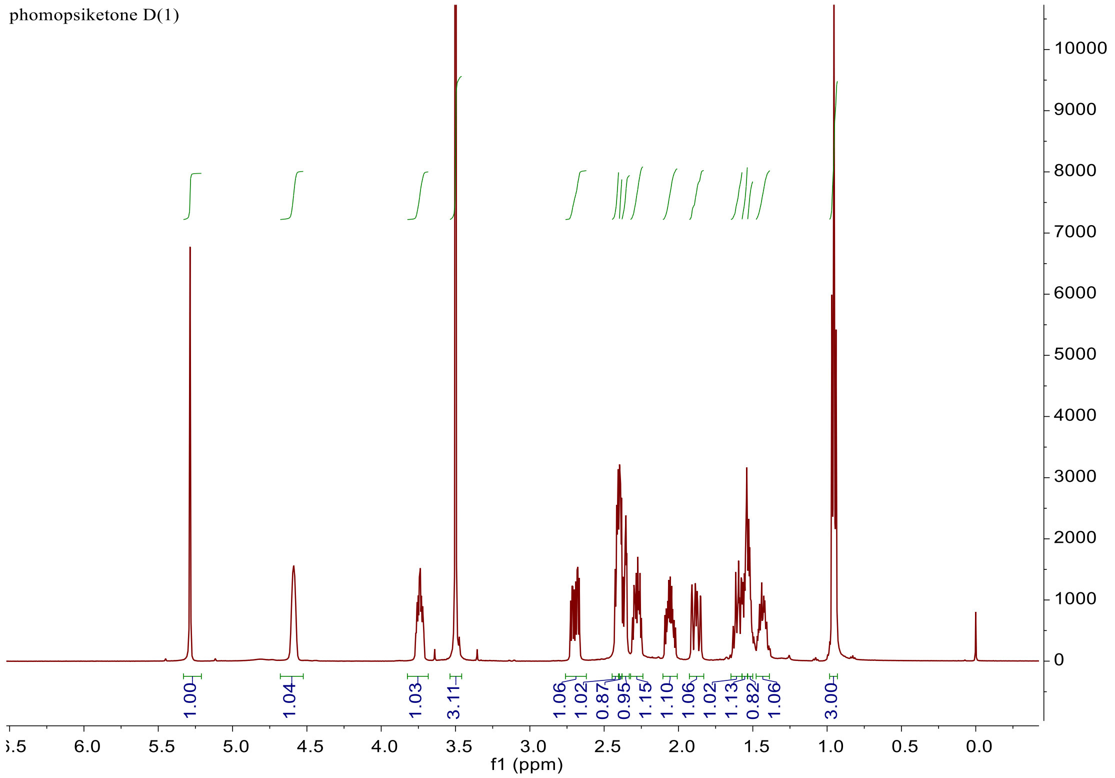

Figure S9. ^{1}H NMR (500 MHz, $CDCl_3$) spectrum of phomopsiketone D (**1**)

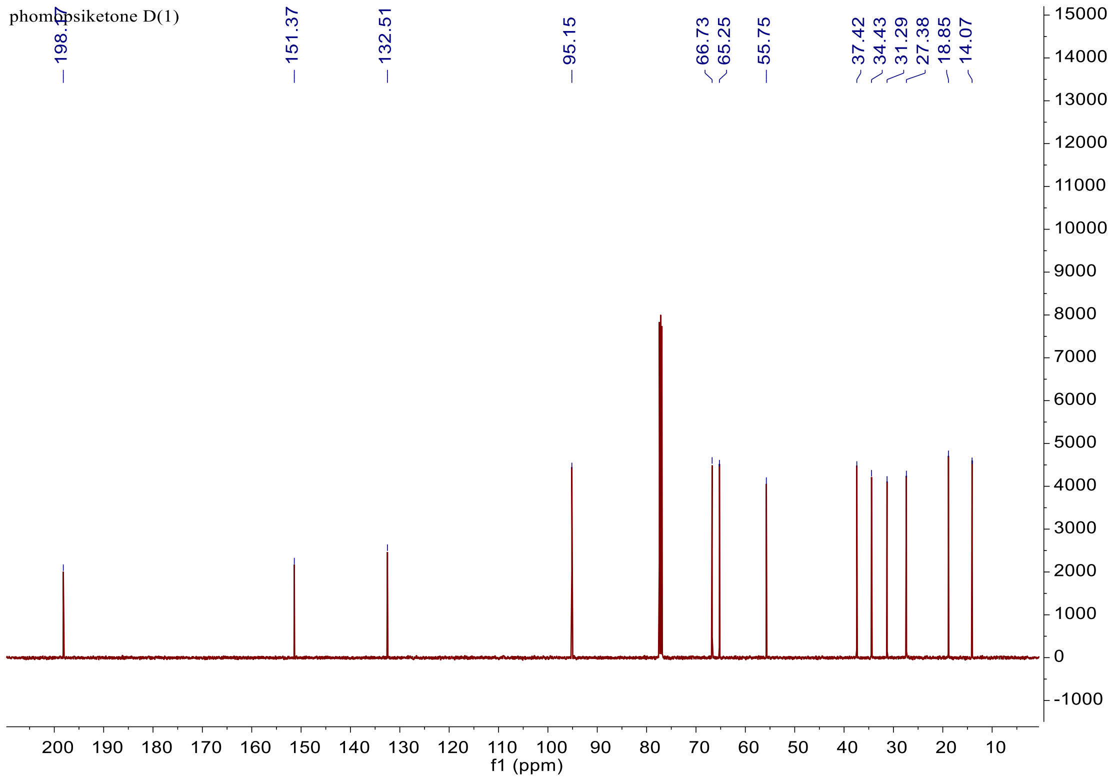

Figure S10. ^{13}C NMR (125 MHz, $CDCl_3$) spectrum of phomopsiketone D (**1**)

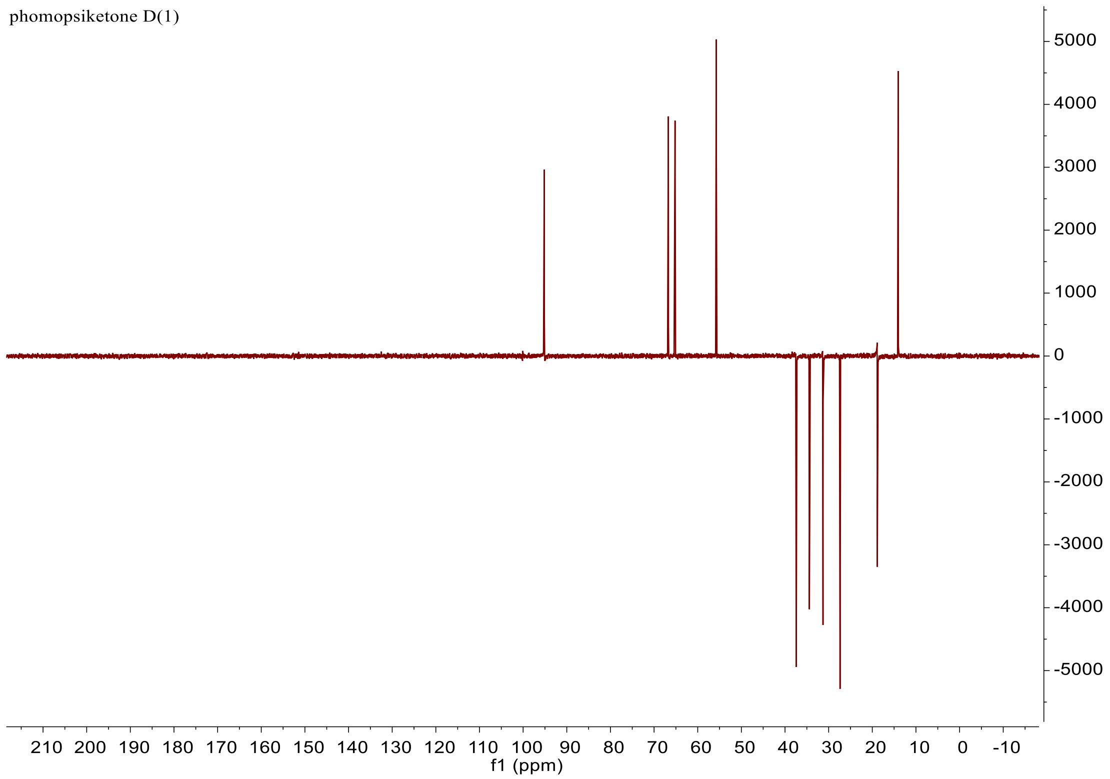

Figure S11. DEPT ($CDCl_3$) spectrum of phomopsiketone D (**1**)

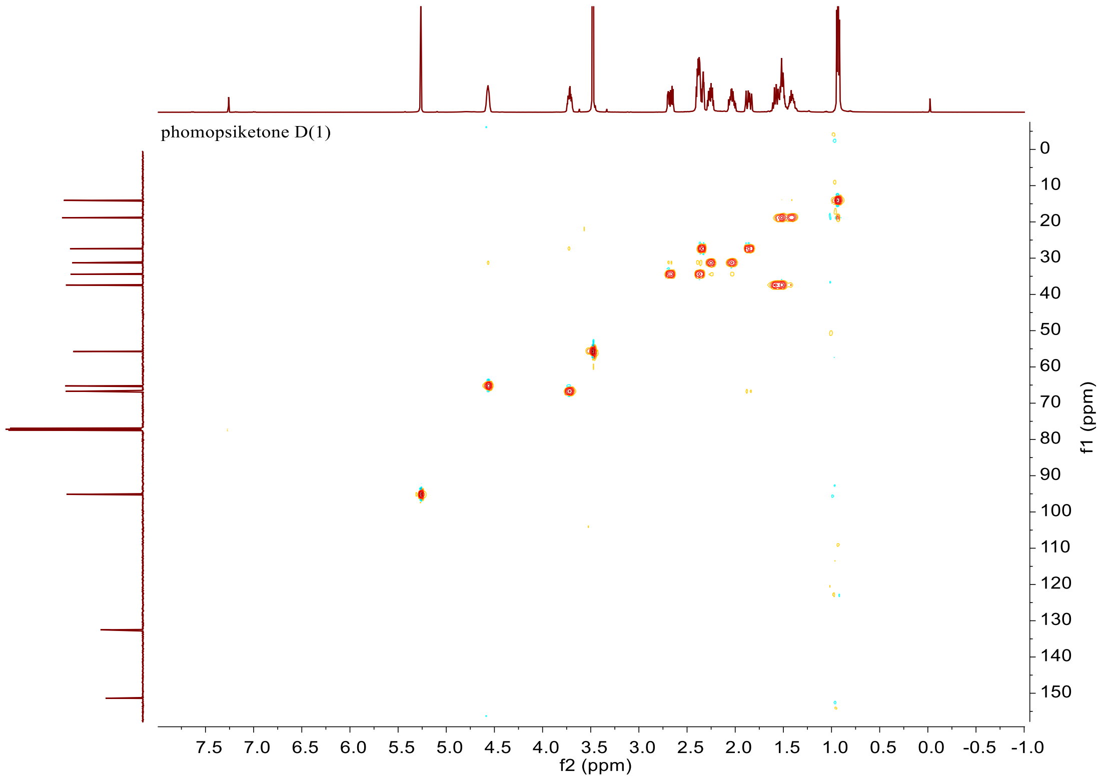

Figure S12. HSQC ($CDCl_3$) spectrum of phomopsiketone D (**1**)

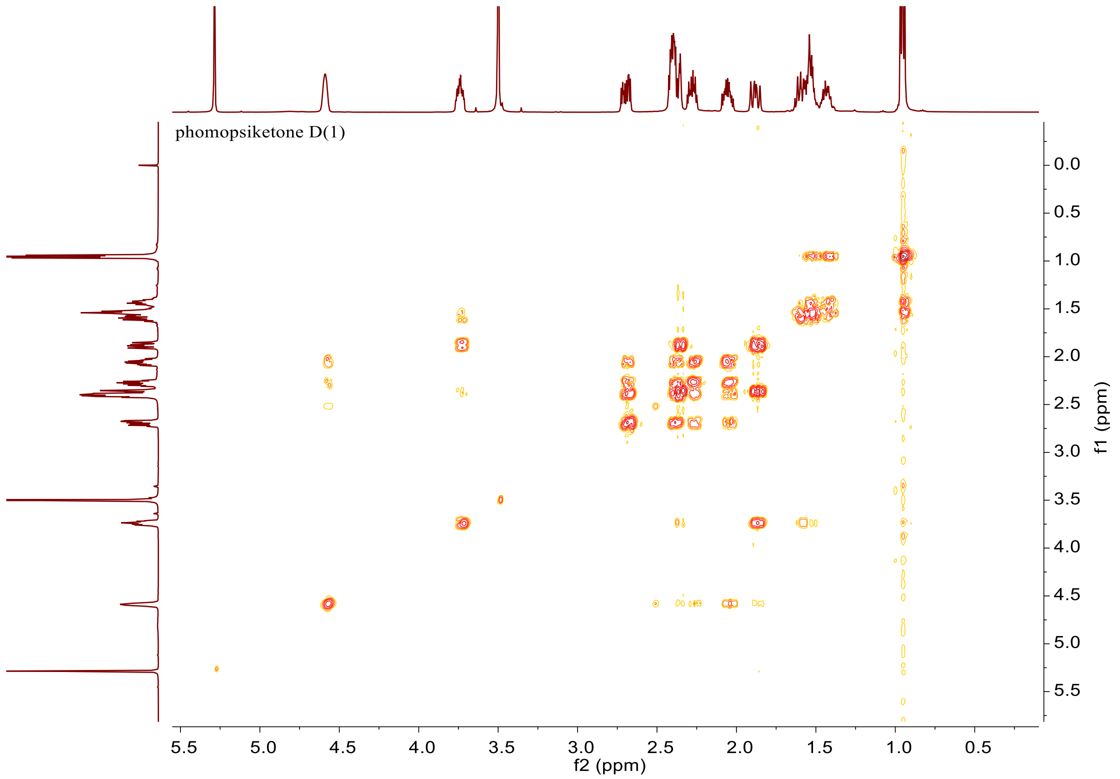

Figure S13. ^1H–^1H COSY ($CDCl_3$) spectrum of phomopsiketone D (**1**)

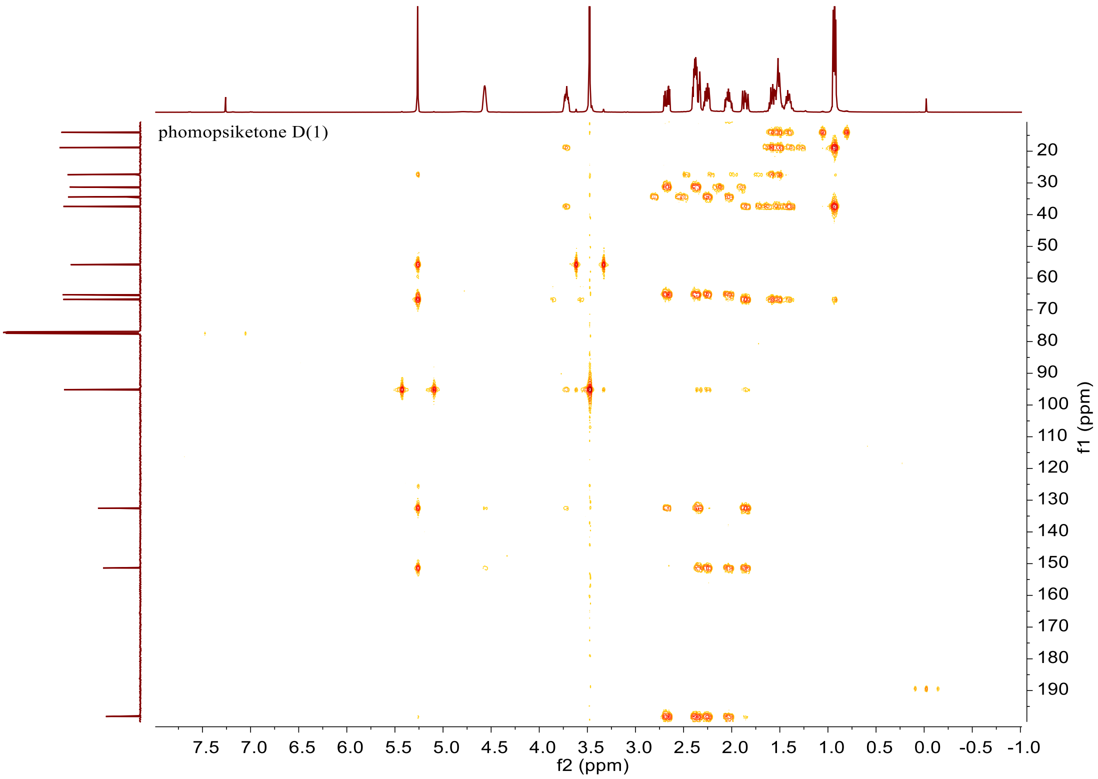

Figure S14. HMBC ($CDCl_3$) spectrum of phomopsiketone D (**1**)

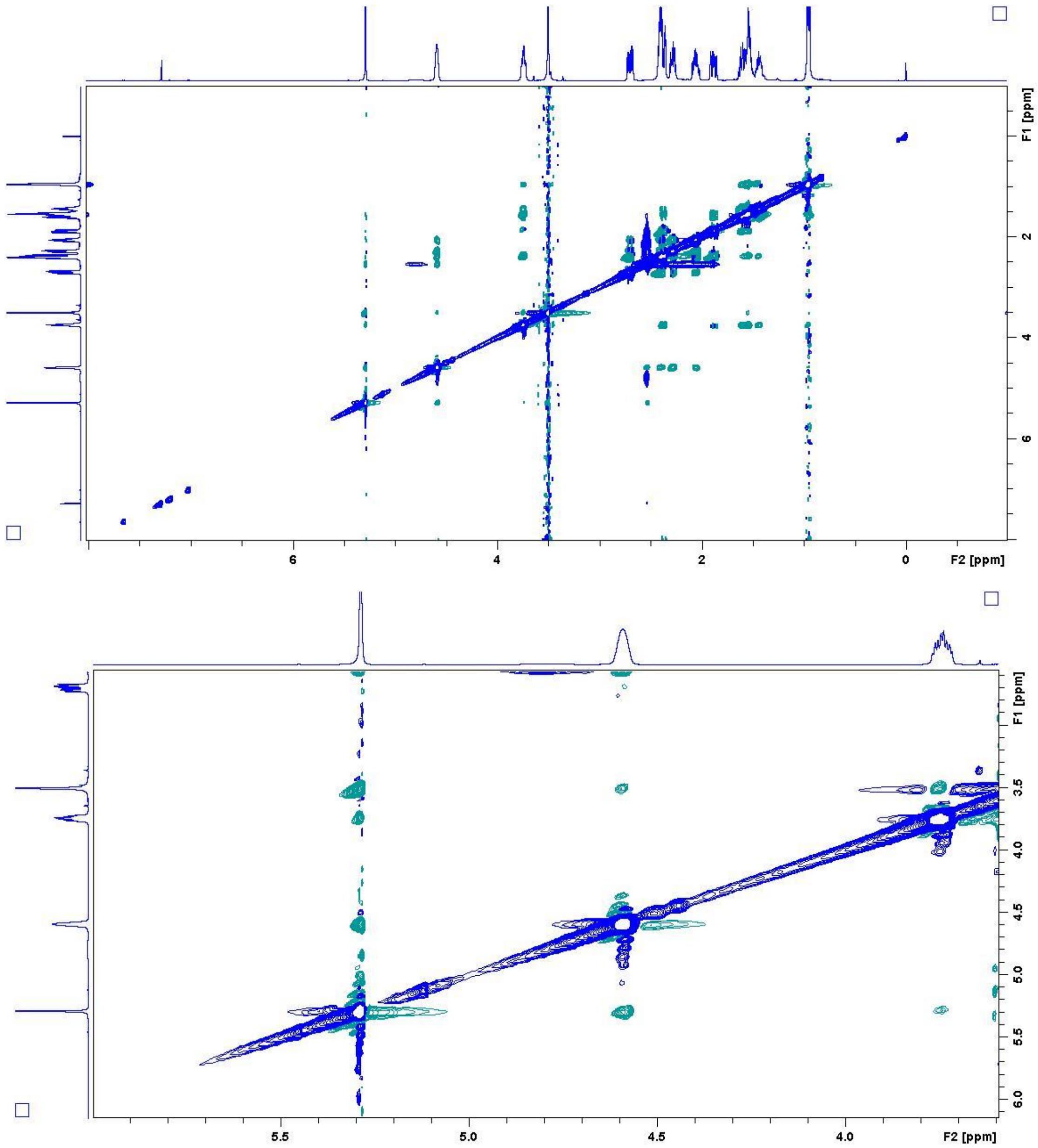

Figure S15. NOESY ($CDCl_3$) spectrum of phomopsiketone D (**1**)

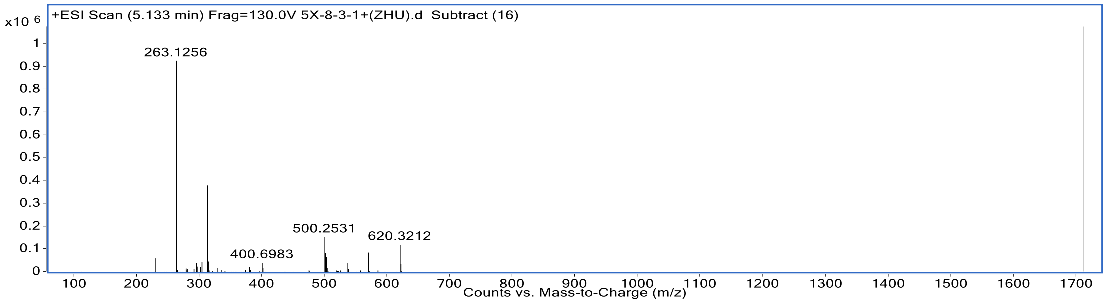

Figure S16. HRESIMS spectrum of phomopsiketone D (**1**)

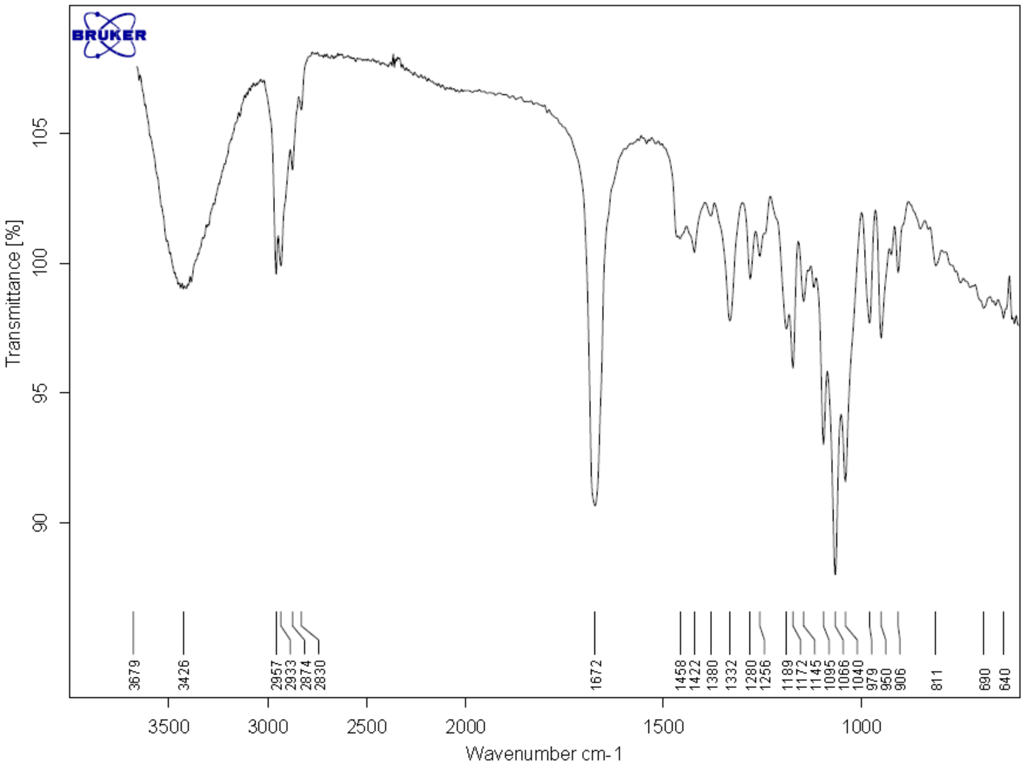

Figure S17. IR spectrum of phomopsiketone D (**1**)

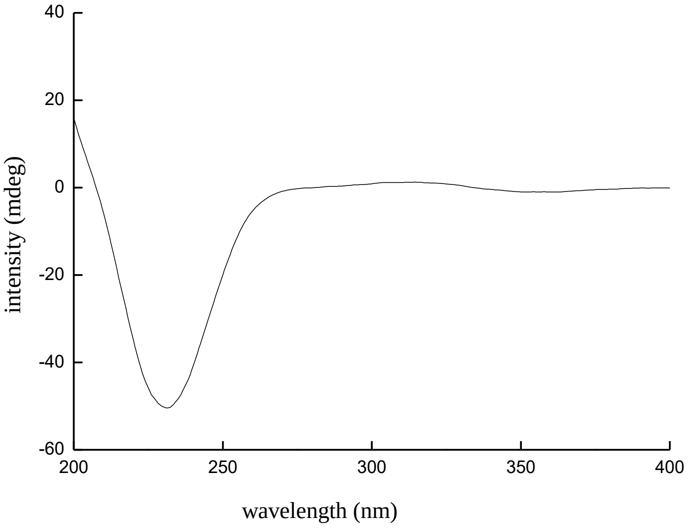

Figure S18. CD spectrum of phomopsiketone D (**1**)

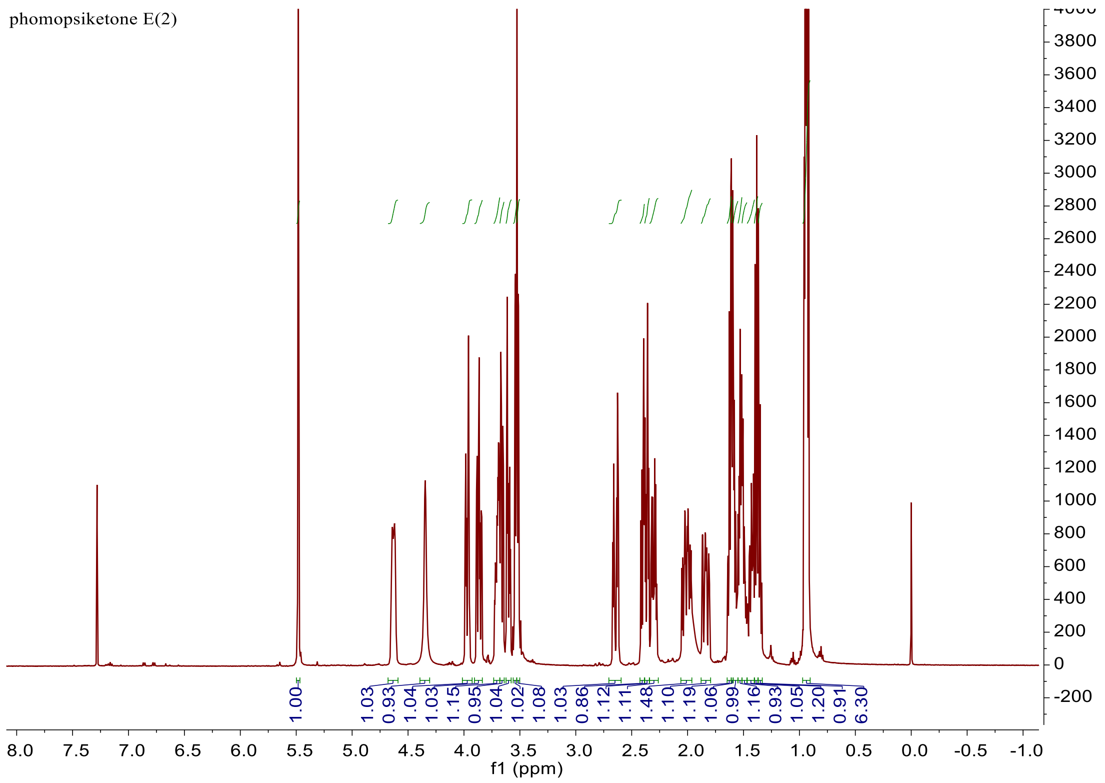

Figure S19. ^{1}H NMR (500 MHz, $CDCl_3$) spectrum of phomopsiketone E (**2**)

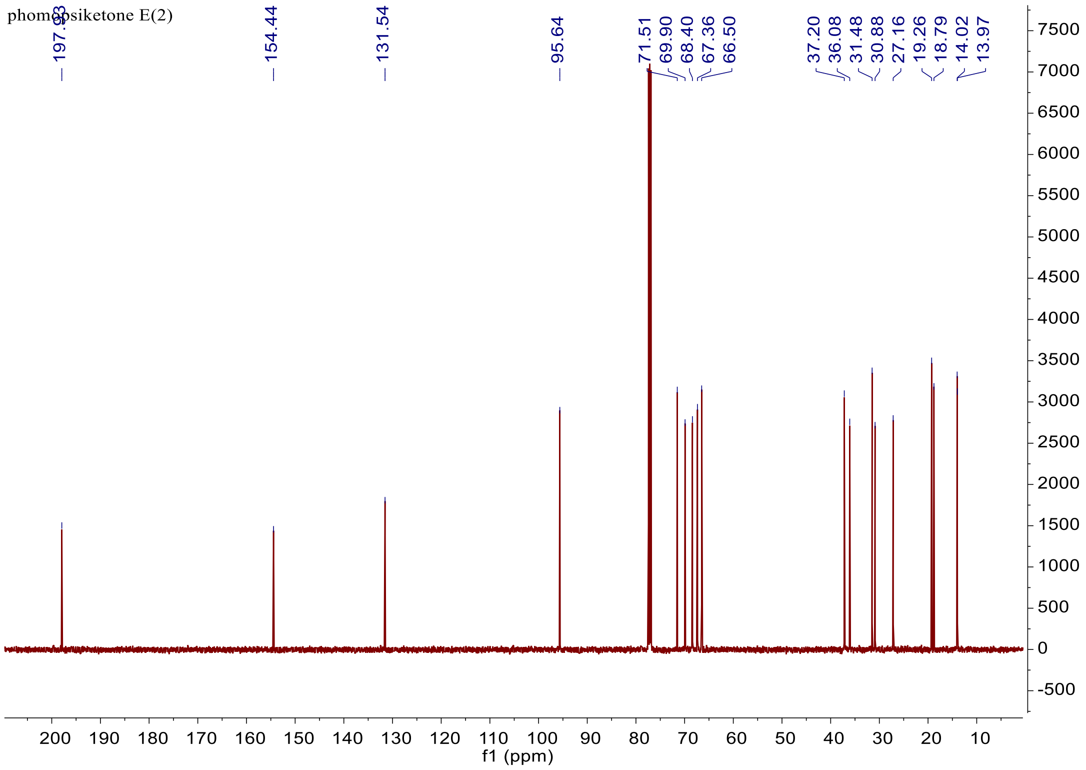

Figure S20. ^{13}C NMR (125 MHz, $CDCl_3$) spectrum of phomopsiketone E (**2**)

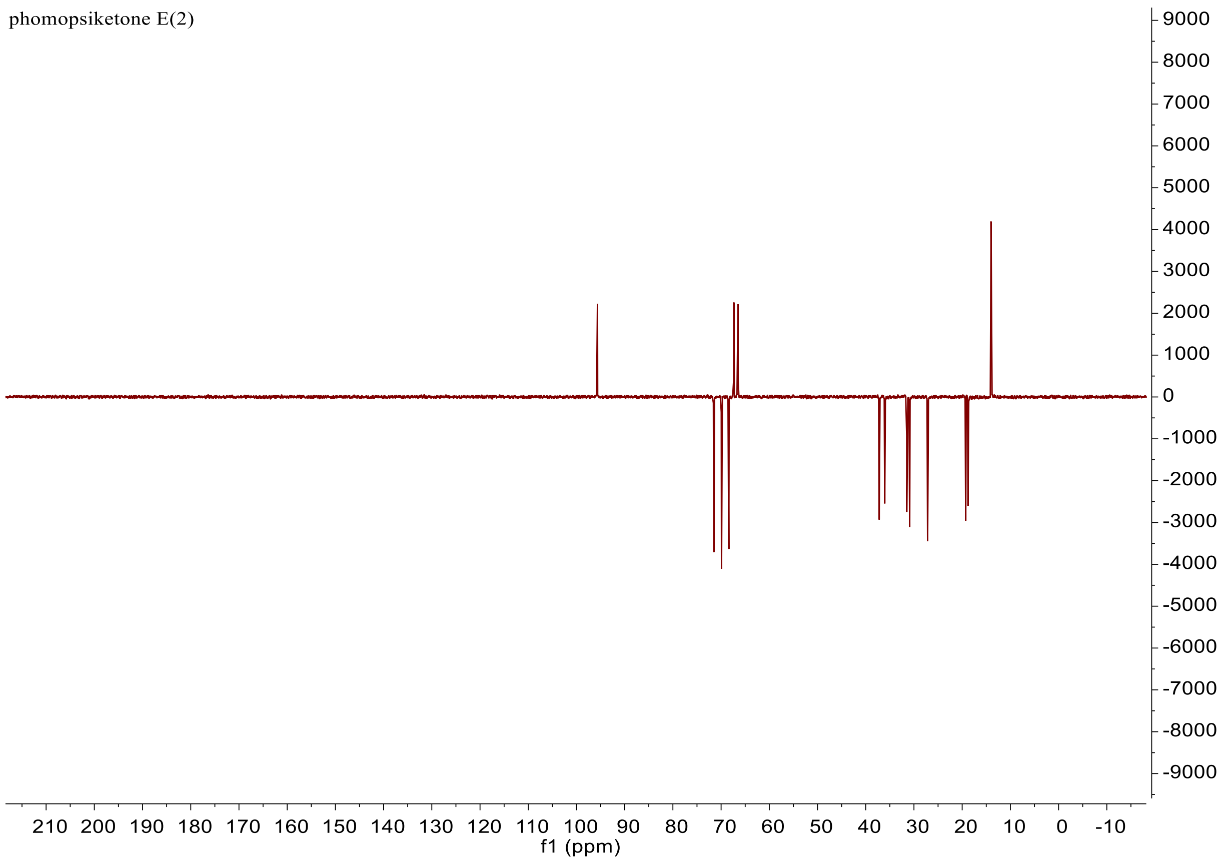

Figure S21. DEPT ($CDCl_3$) spectrum of phomopsiketone E (**2**)

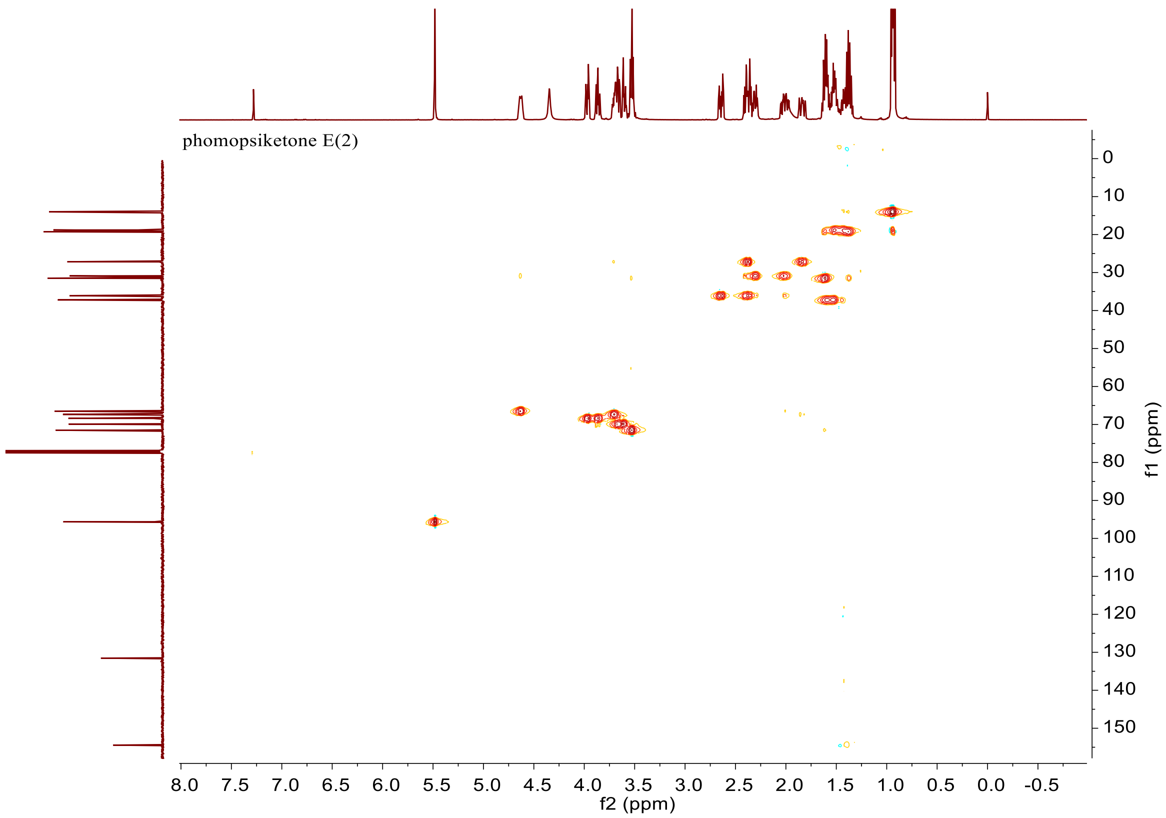

Figure S22. HSQC ($CDCl_3$) spectrum of phomopsiketone E (**2**)

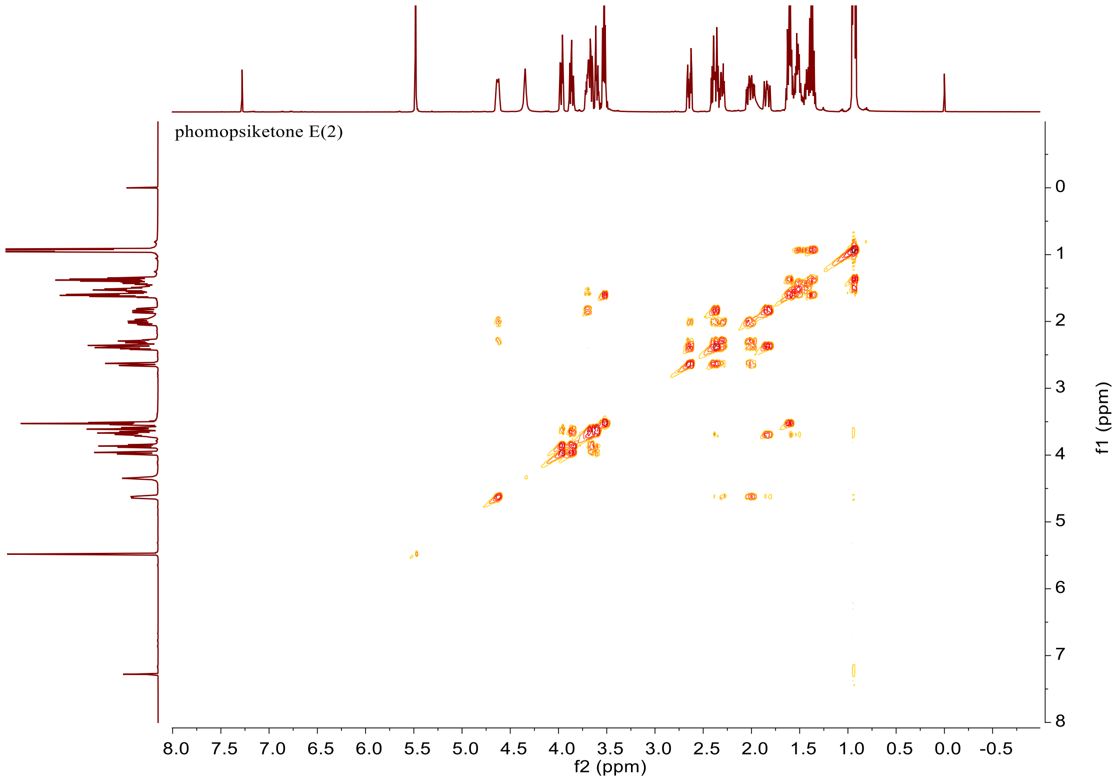

Figure S23. ^1H–^1H COSY ($CDCl_3$) spectrum of phomopsiketone E (**2**)

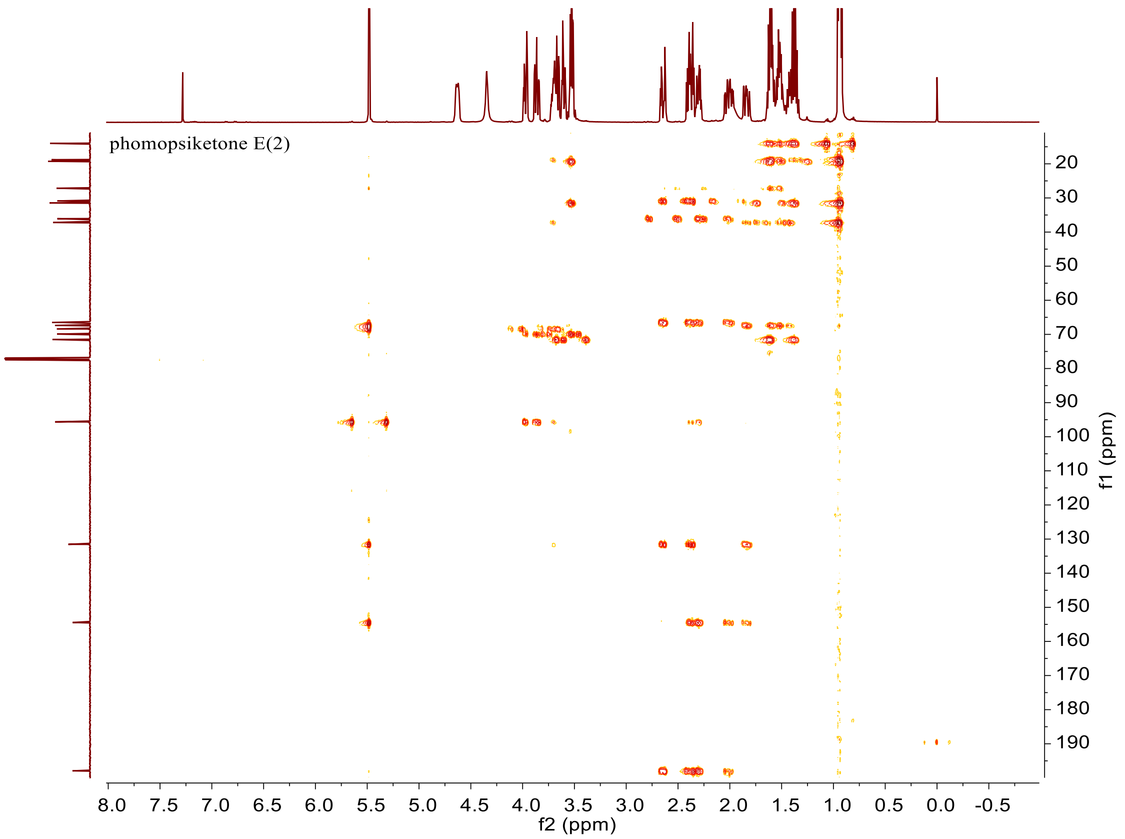

Figure S24. HMBC ($CDCl_3$) spectrum of phomopsiketone E (**2**)

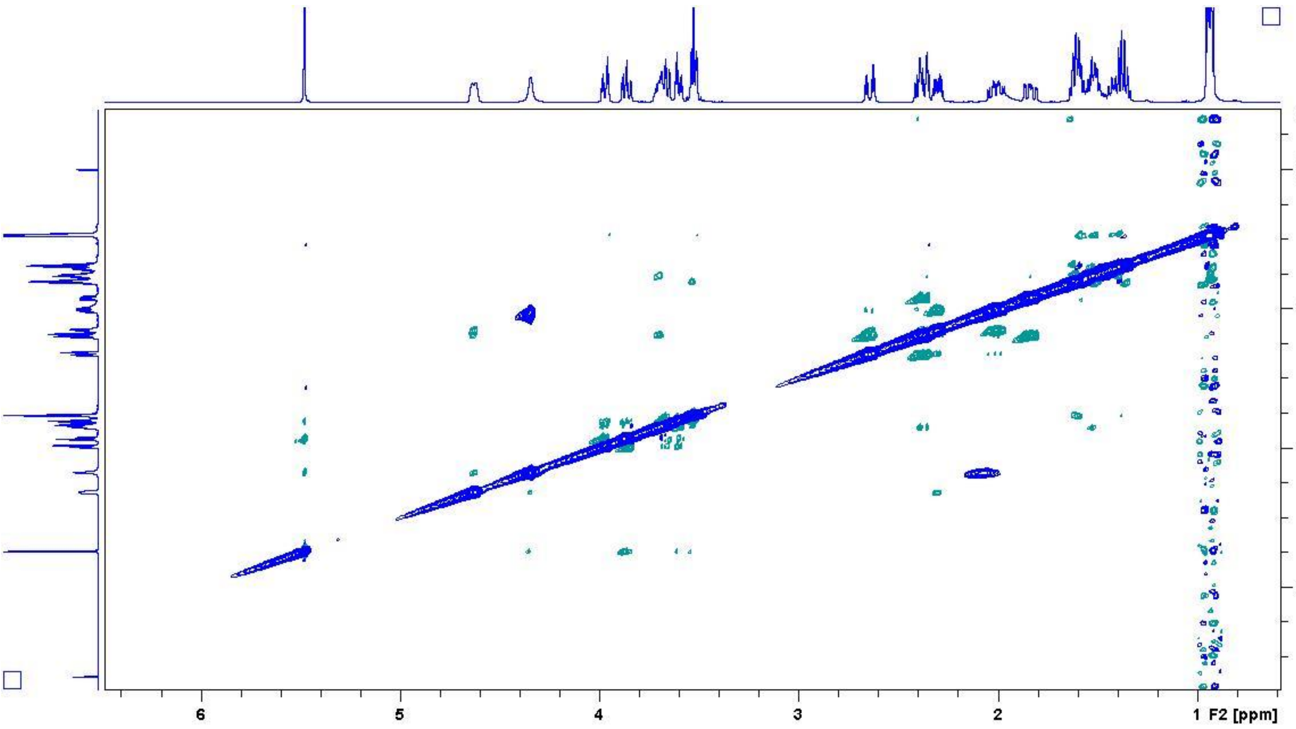

Figure S25. NOESY (CDCl₃) spectrum of phomopsiketone E (**2**)

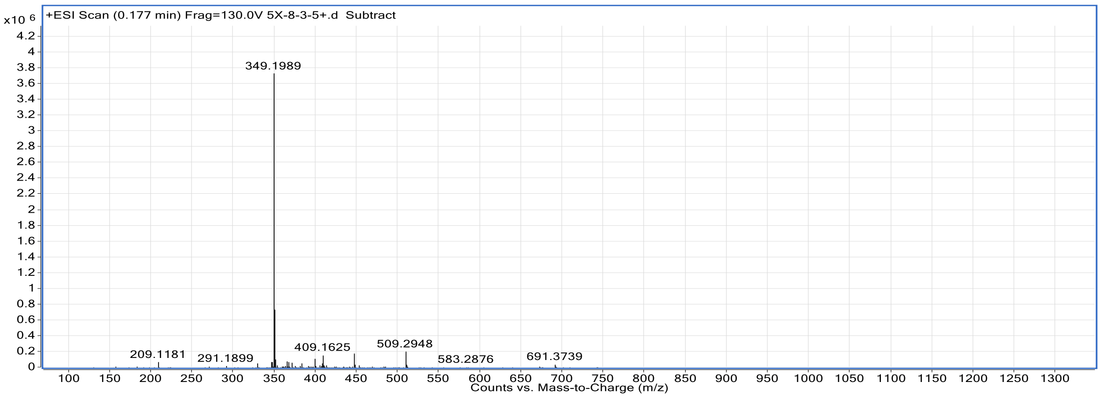

Figure S26. HRESIMS spectrum of phomopsiketone E (**2**)

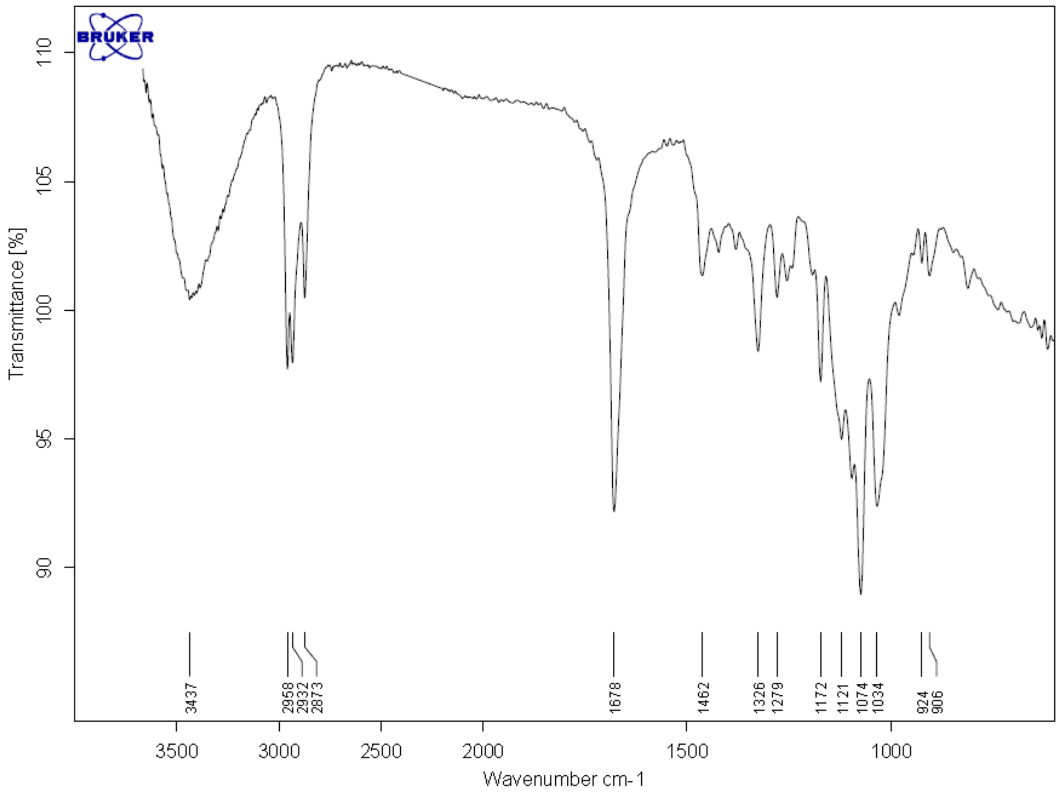

Figure S27. IR spectrum of phomopsiketone E (**2**)

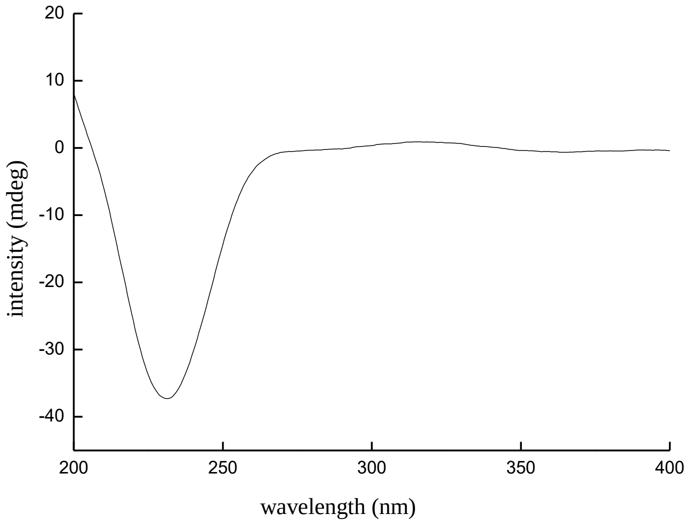

Figure S28. CD spectrum of phomopsiketone E (**2**)

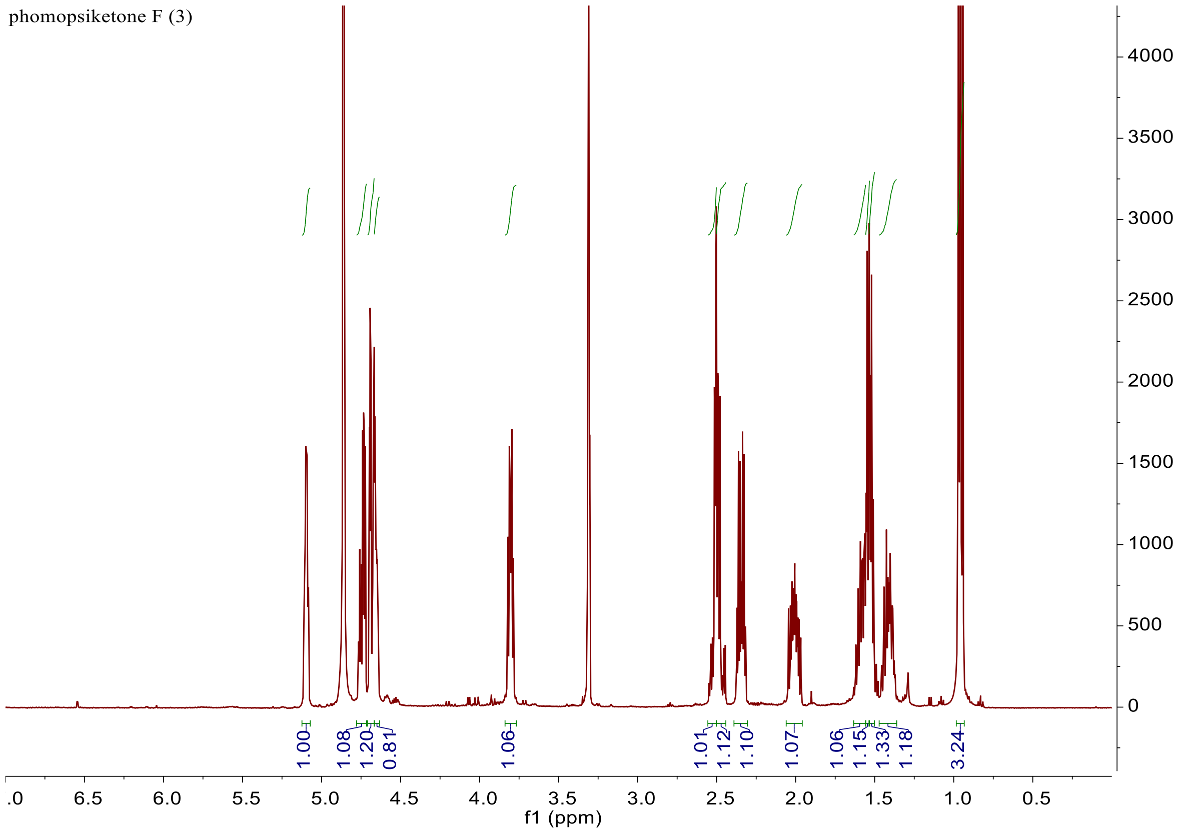

Figure S29. ^{1}H NMR (500 MHz, CD_3OD) spectrum of phomopsiketone F (**3**)

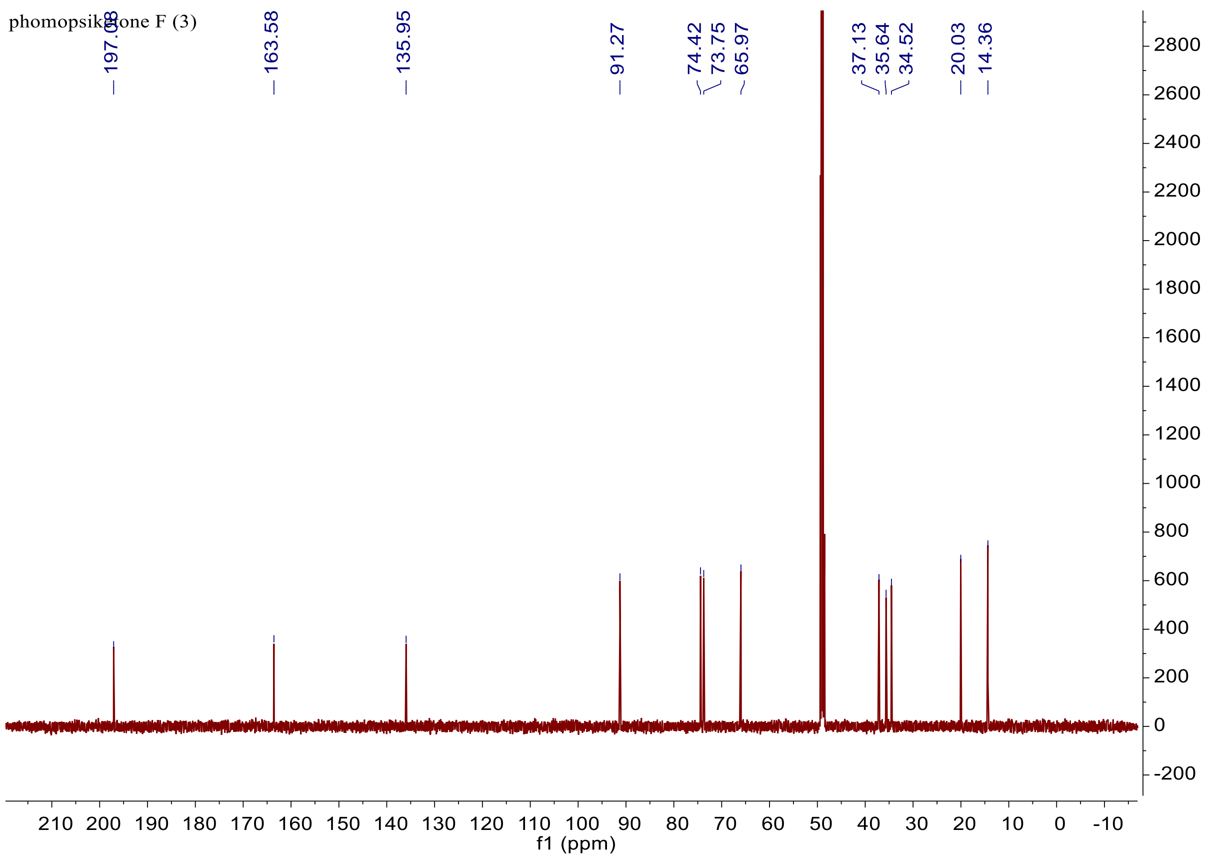

Figure S30. ^{13}C NMR (125 MHz, CD_3OD) spectrum of phomopsiketone F (**3**)

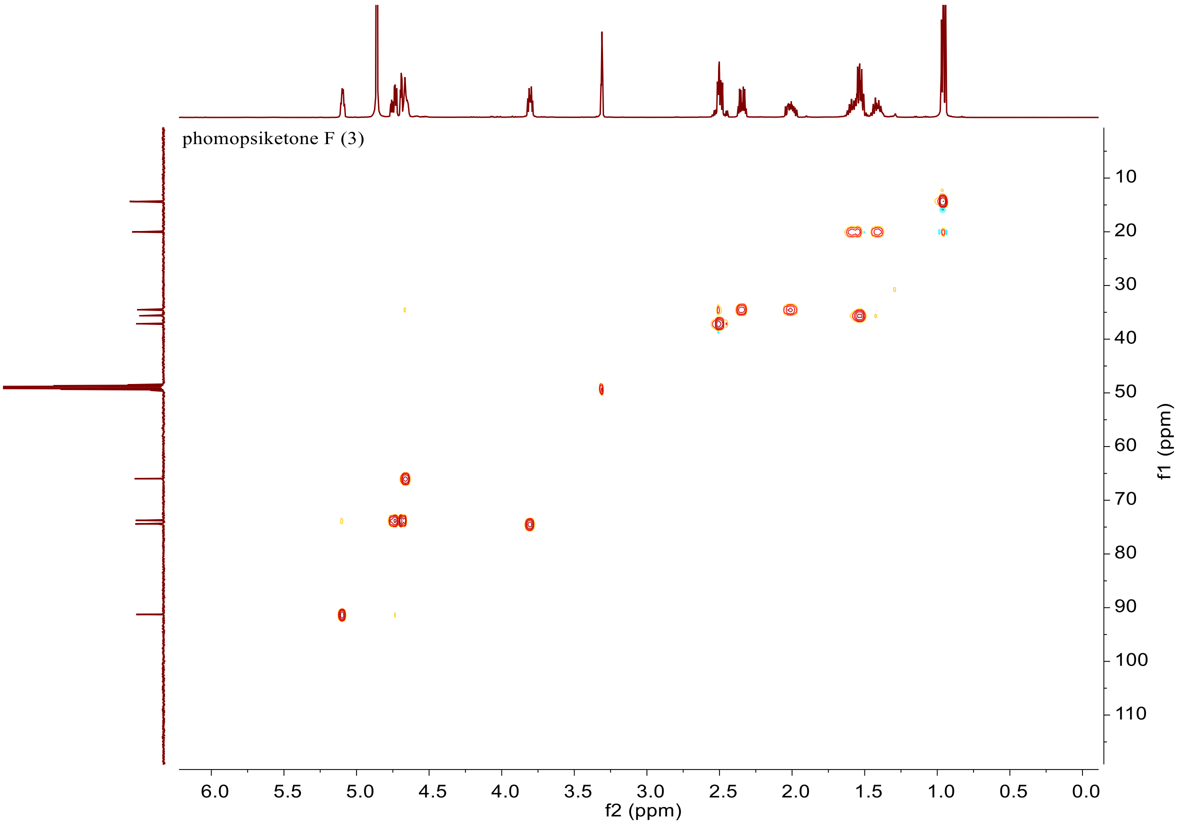

Figure S31. HSQC (CD_3OD) spectrum of phomopsiketone F (**3**)

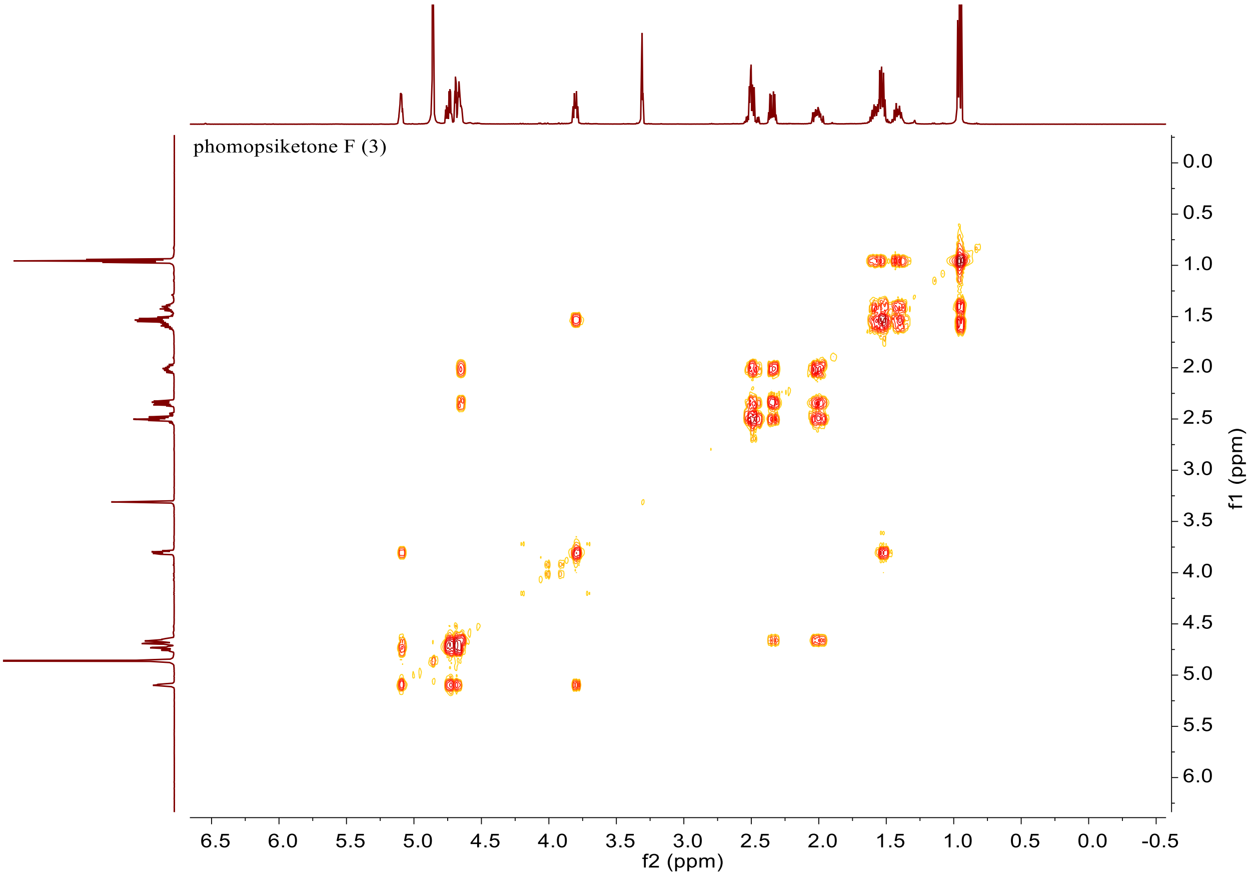

Figure S32. ^{1}H–^{1}H COSY (CD_3OD) spectrum of phomopsiketone F (**3**)

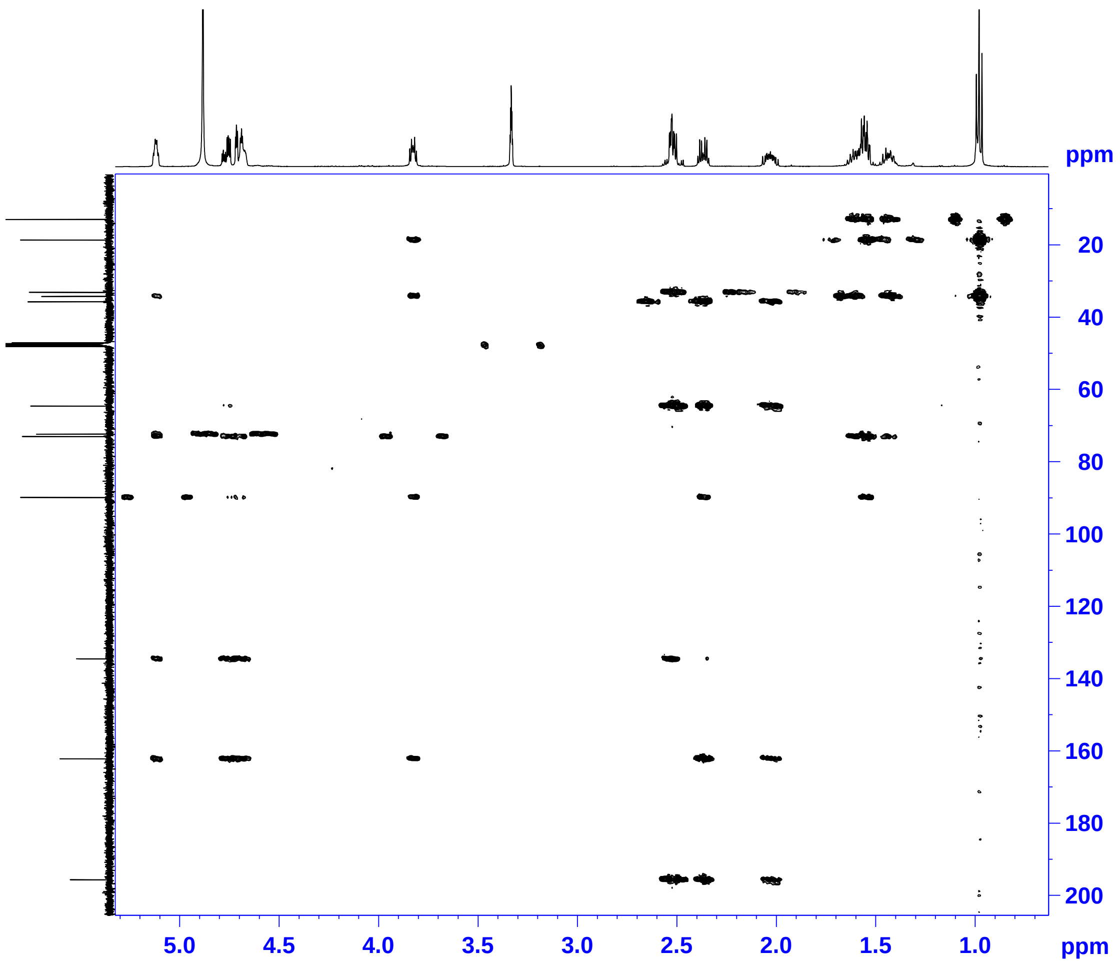

Figure S33. HMBC (CD_3OD) spectrum of phomopsiketone F (**3**)

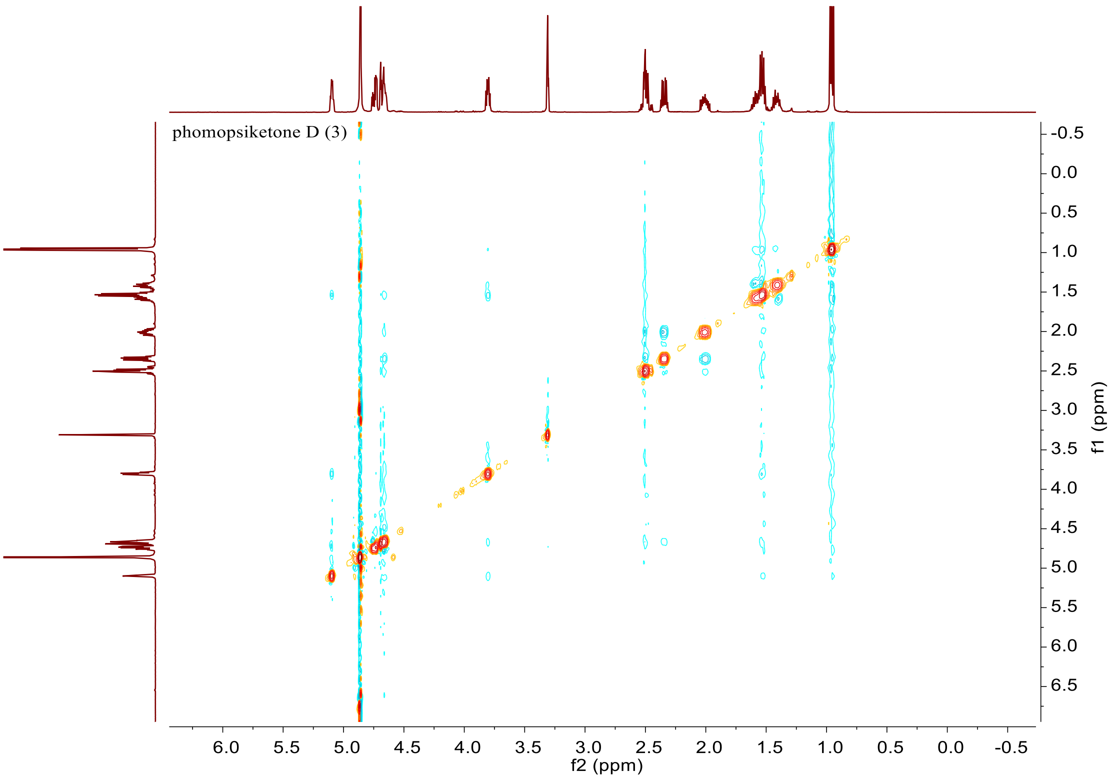

Figure S34. NOESY (CD_3OD) spectrum of phomopsiketone F (**3**)

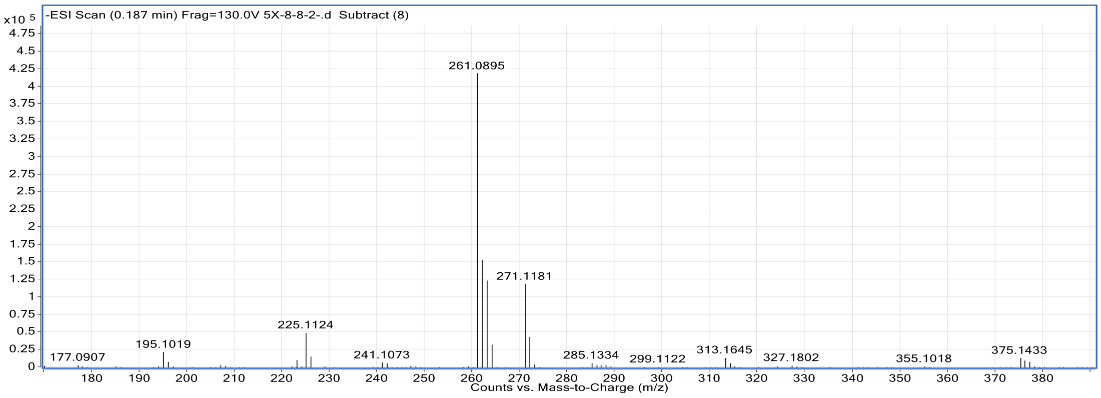

Figure S35. HRESIMS spectrum of phomopsiketone F (**3**)

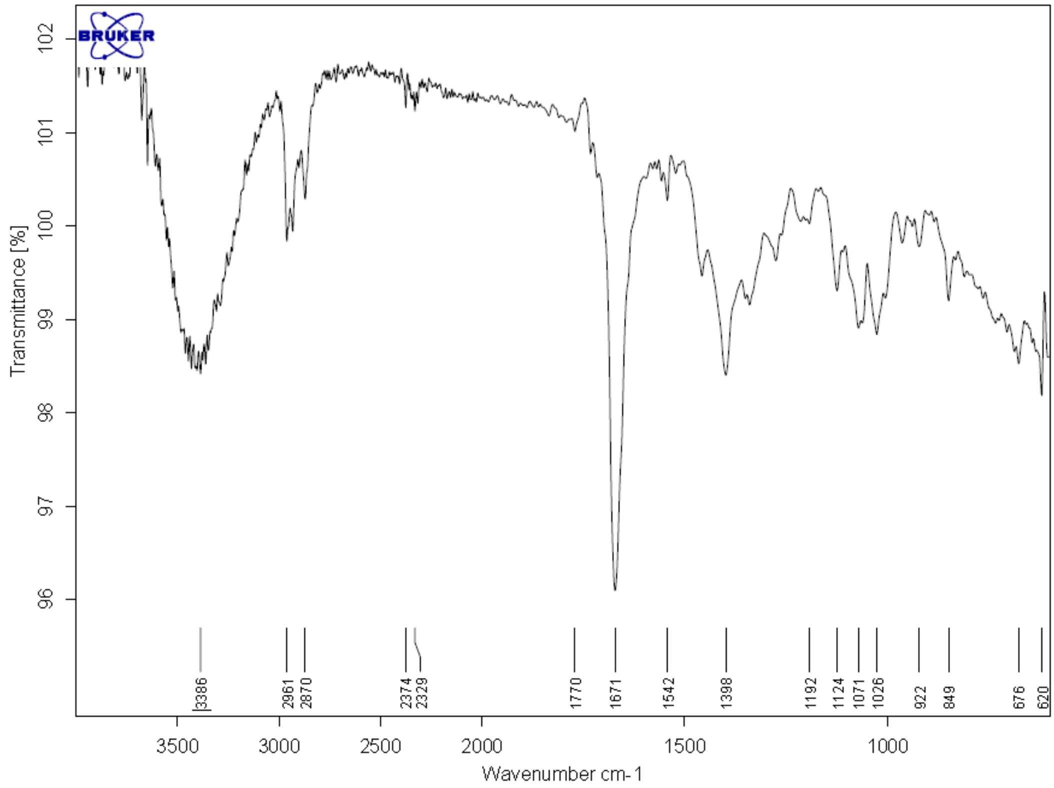

Figure S36. IR spectrum of phomopsiketone F (**3**)

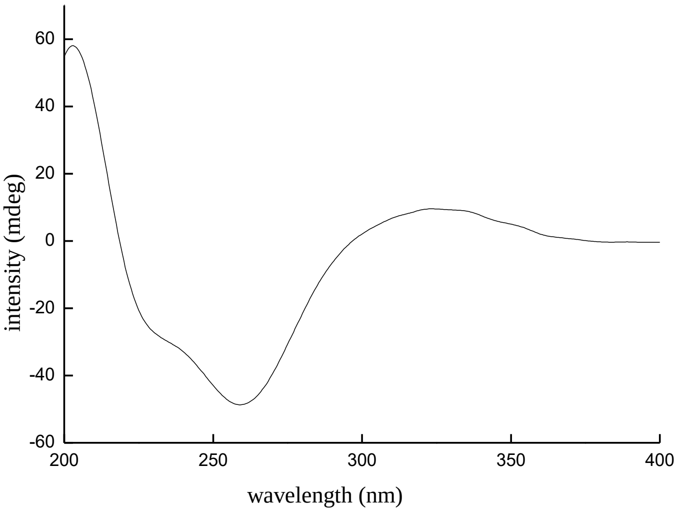

Figure S37. CD spectrum of phomopsiketone F (**3**)

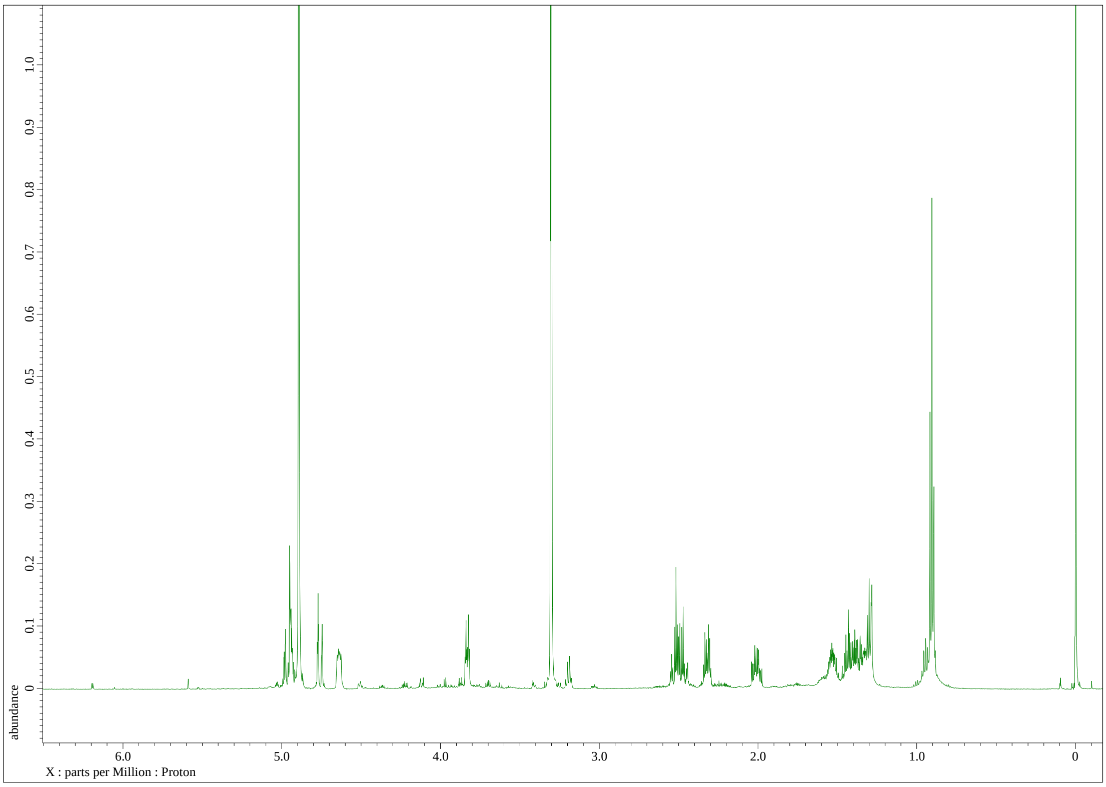

Figure S38. ^{1}H NMR (600 MHz, CD_3OD) spectrum of phomopsiketone G (**4**)

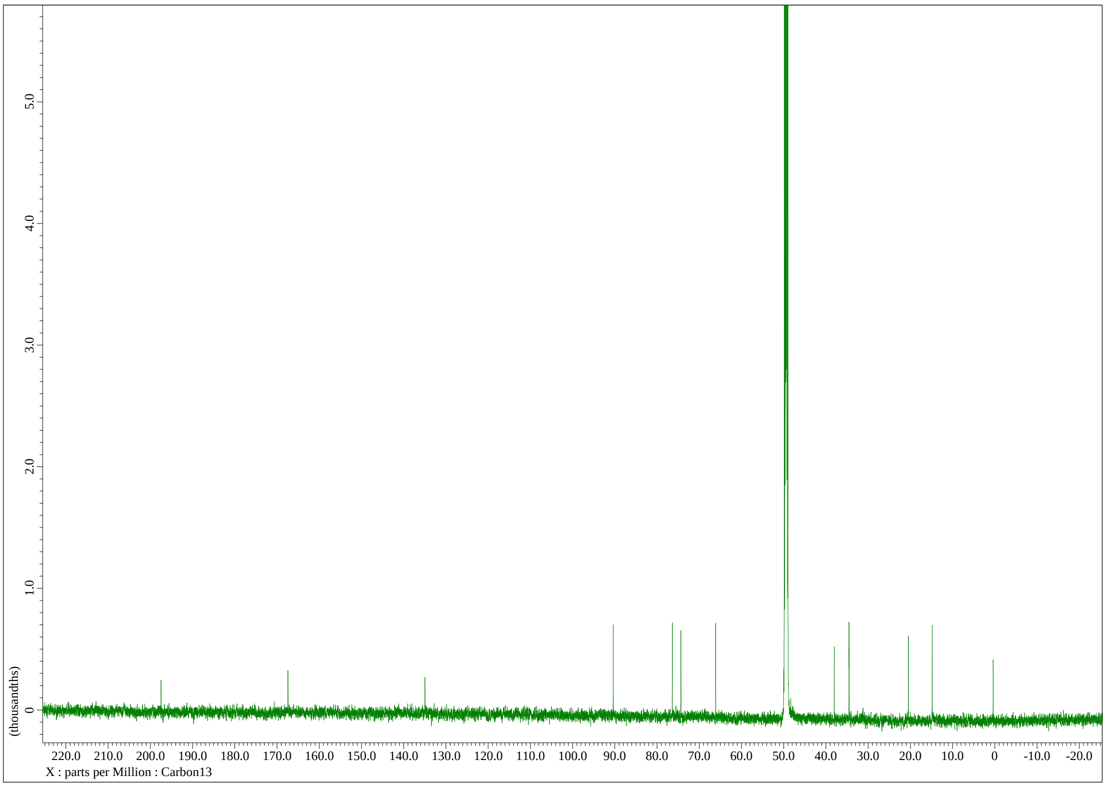

Figure S39. ^{13}C NMR (150 MHz, CD_3OD) spectrum of phomopsiketone G (**4**)

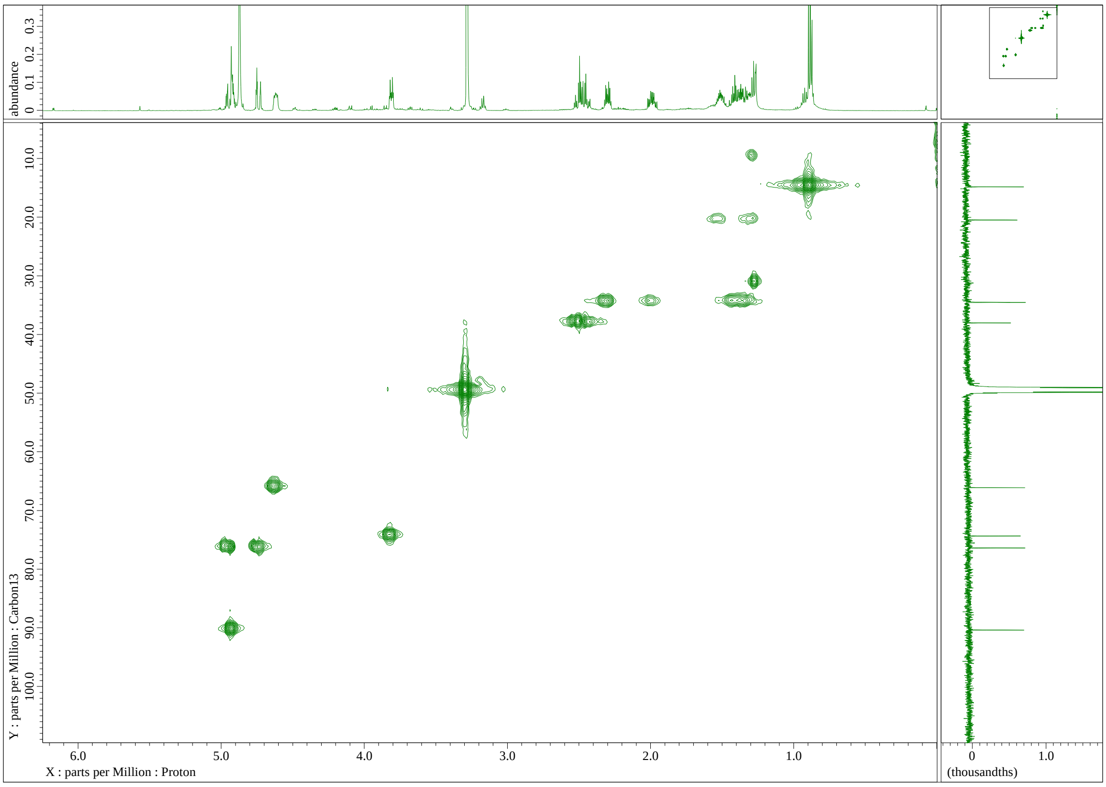

Figure S40. HSQC (CD_3OD) spectrum of phomopsiketone G (**4**)

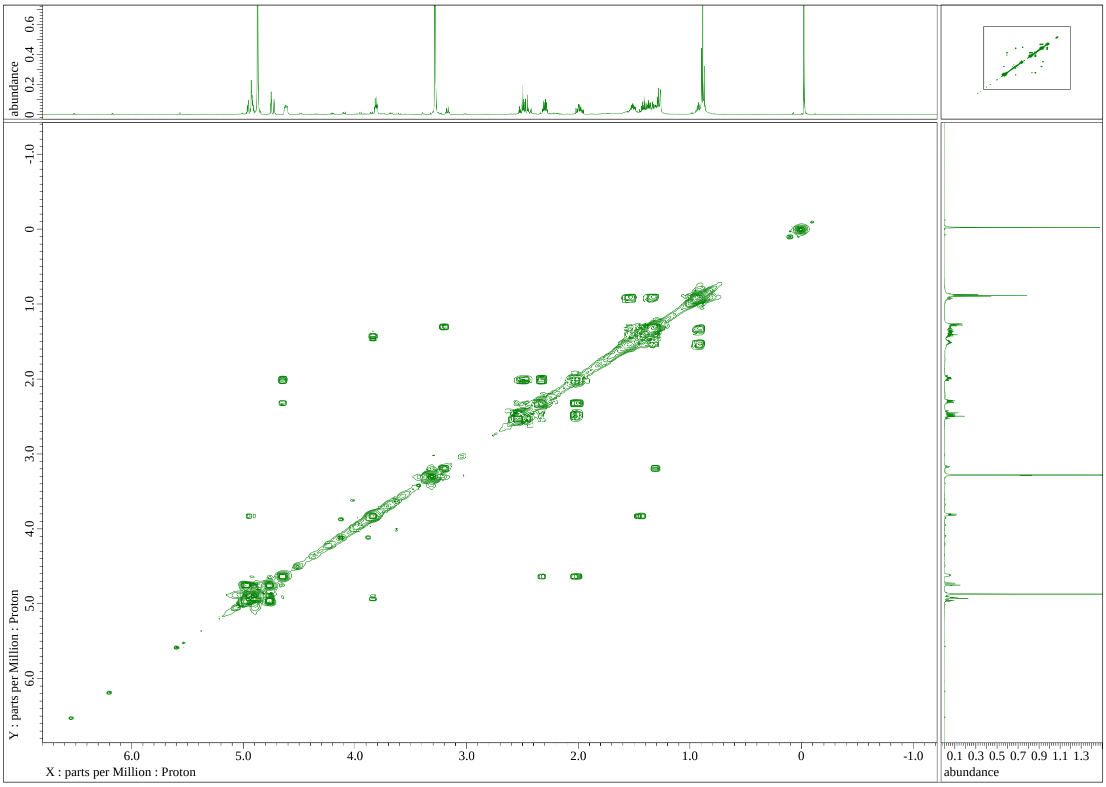

Figure S41. ^1H–^1H COSY (CD_3OD) spectrum of phomopsiketone G (**4**)

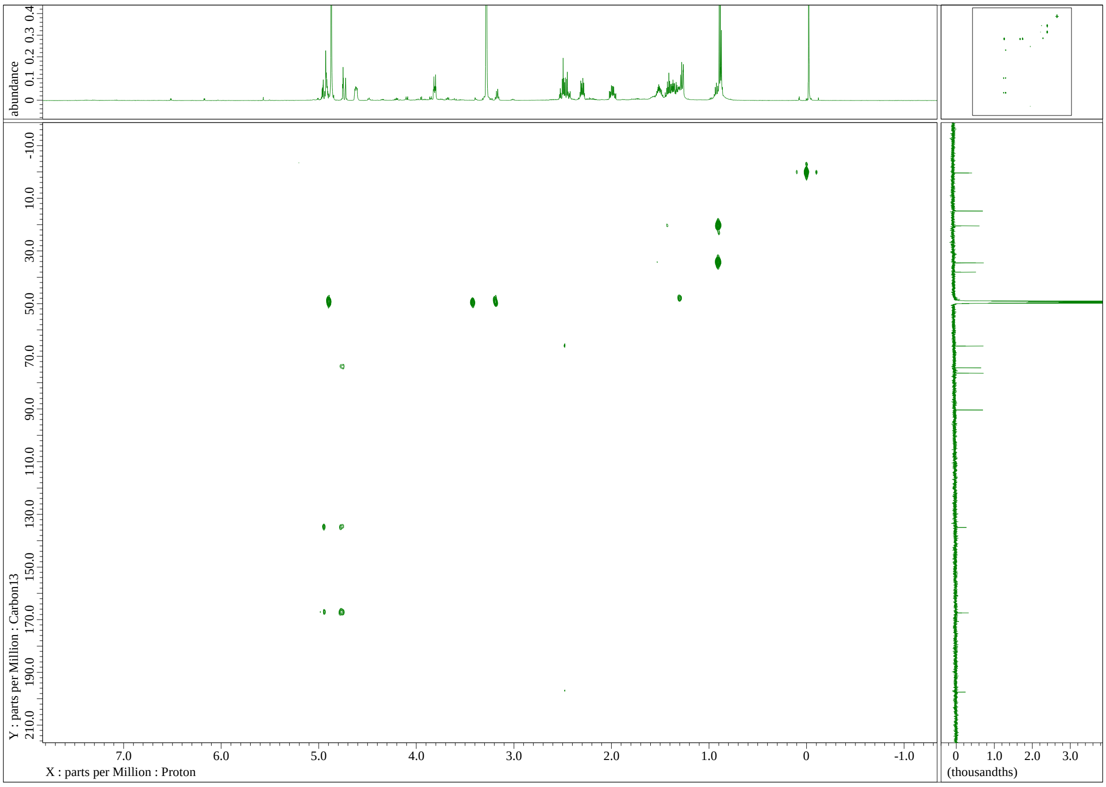

Figure S42. HMBC (CD_3OD) spectrum of phomopsiketone G (**4**)

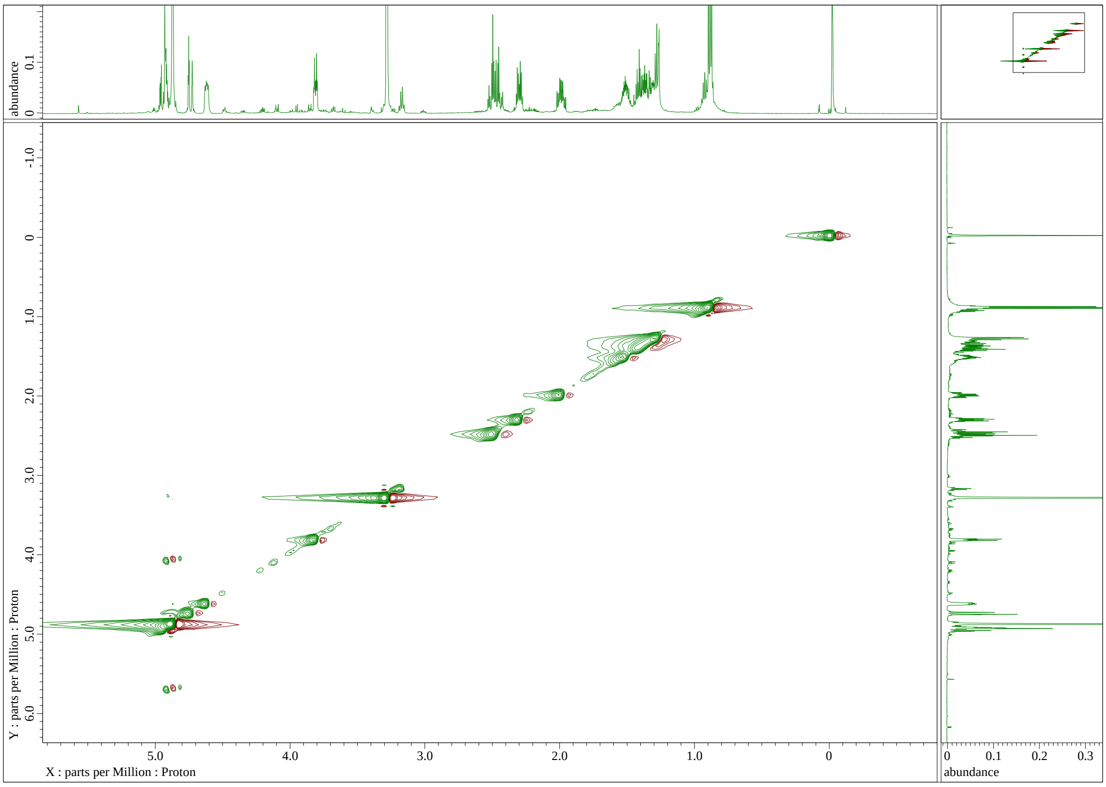

Figure S43. NOESY (CD_3OD) spectrum of phomopsiketone G (**4**)

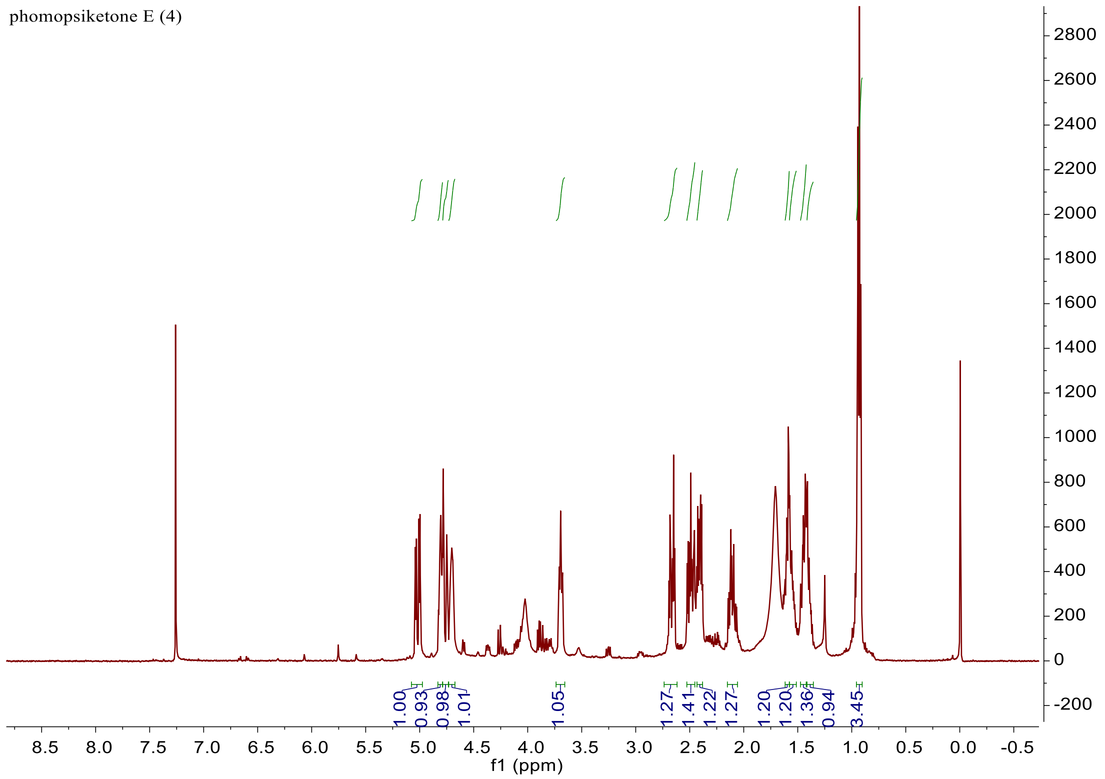

Figure S44. ^{1}H NMR (500 MHz, $CDCl_3$) spectrum of phomopsiketone G (**4**)

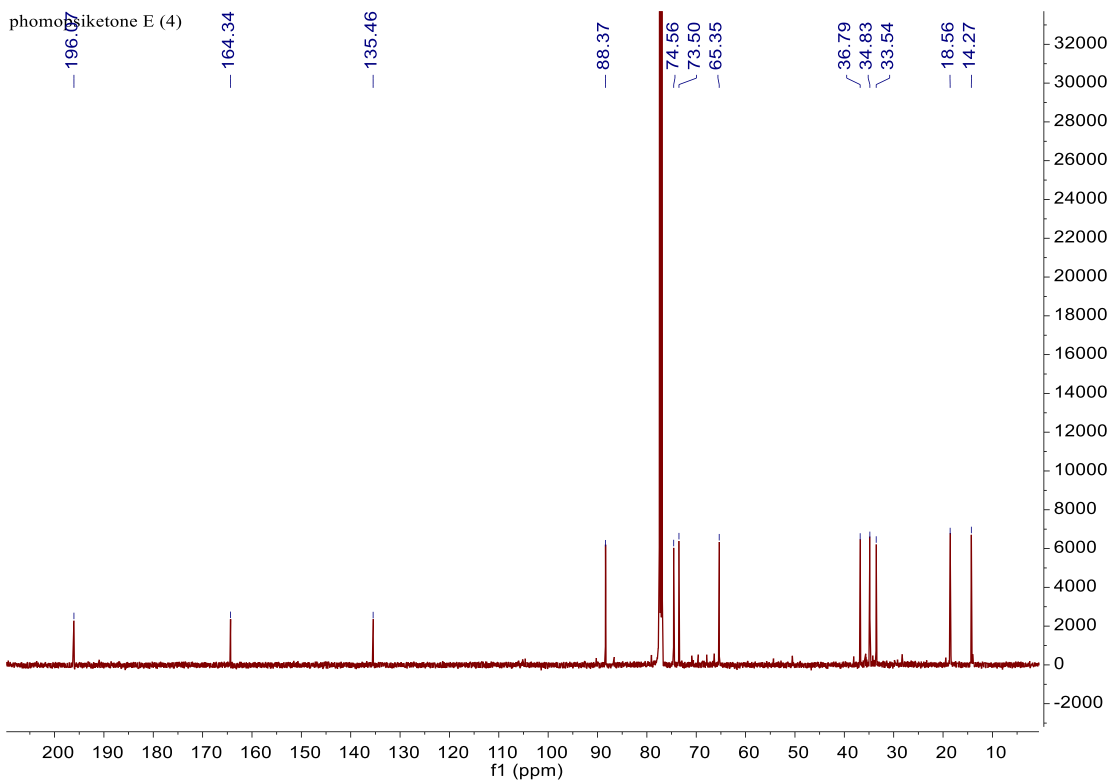

Figure S45. ^{13}C NMR (125 MHz, $CDCl_3$) spectrum of phomopsiketone G (**4**)

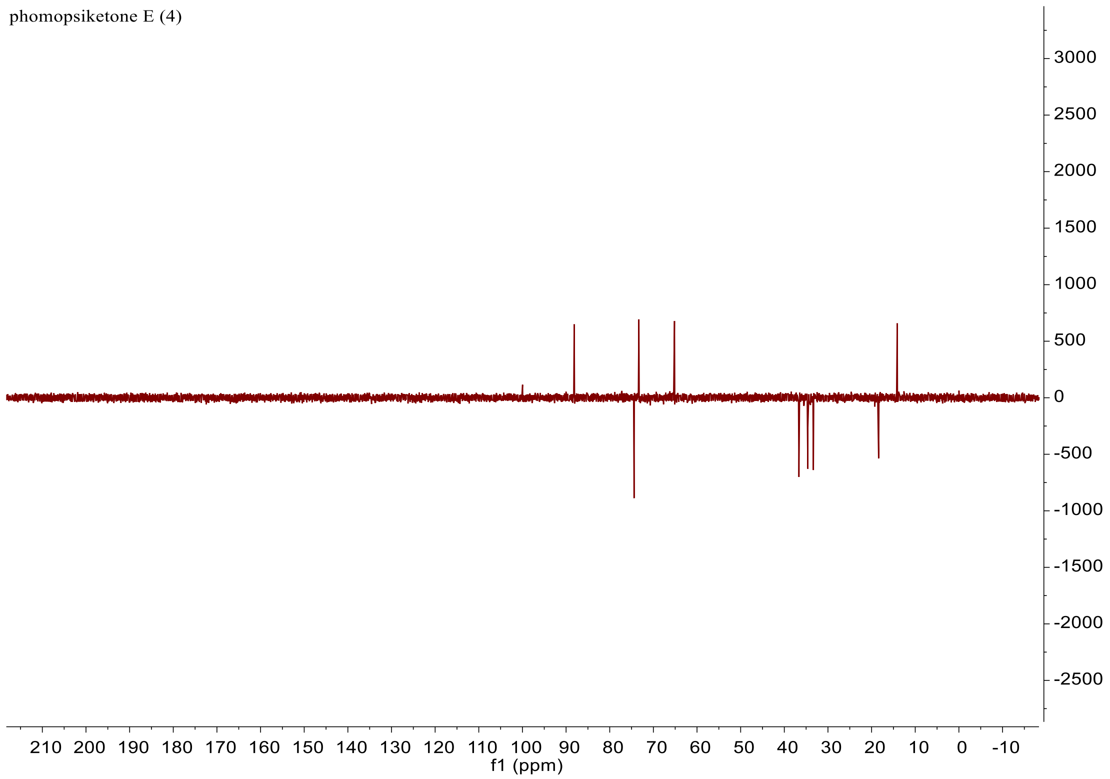

Figure S46. DEPT (CDCl3) spectrum of phomopsiketone G (**4**)

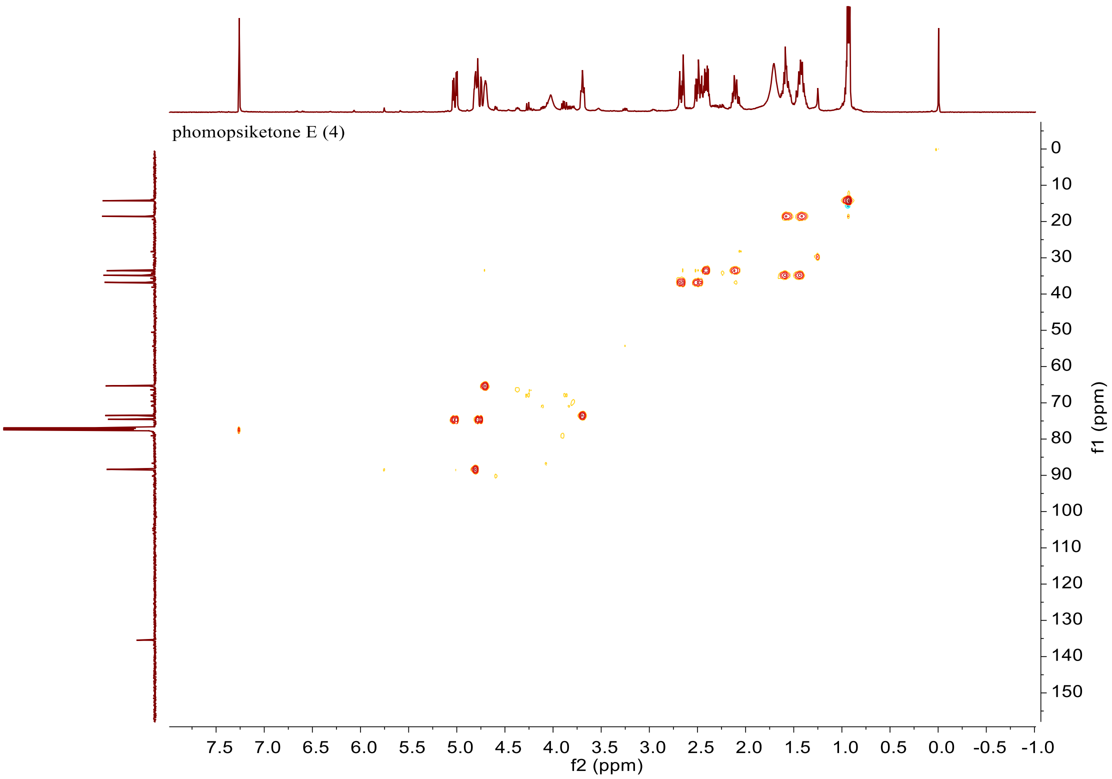

Figure S47. HSQC (CD_3OD) spectrum of phomopsiketone G (**4**)

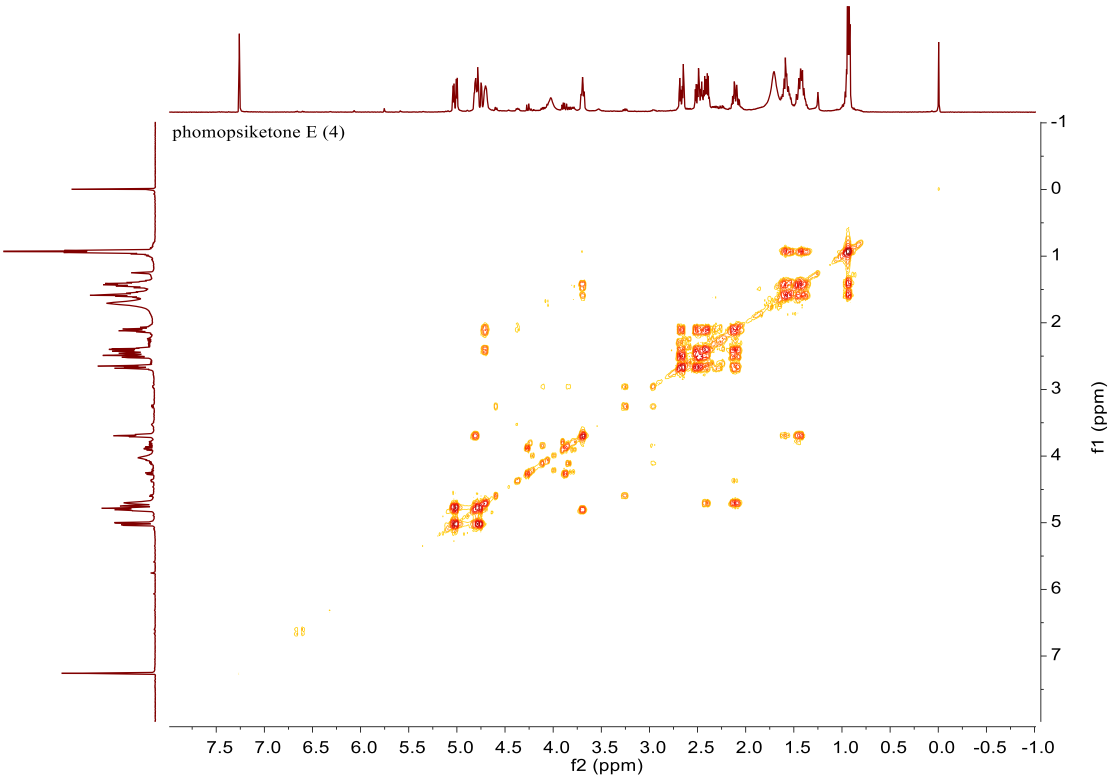

Figure S48. ^1H–^1H COSY ($CDCl_3$) spectrum of phomopsiketone G (**4**)

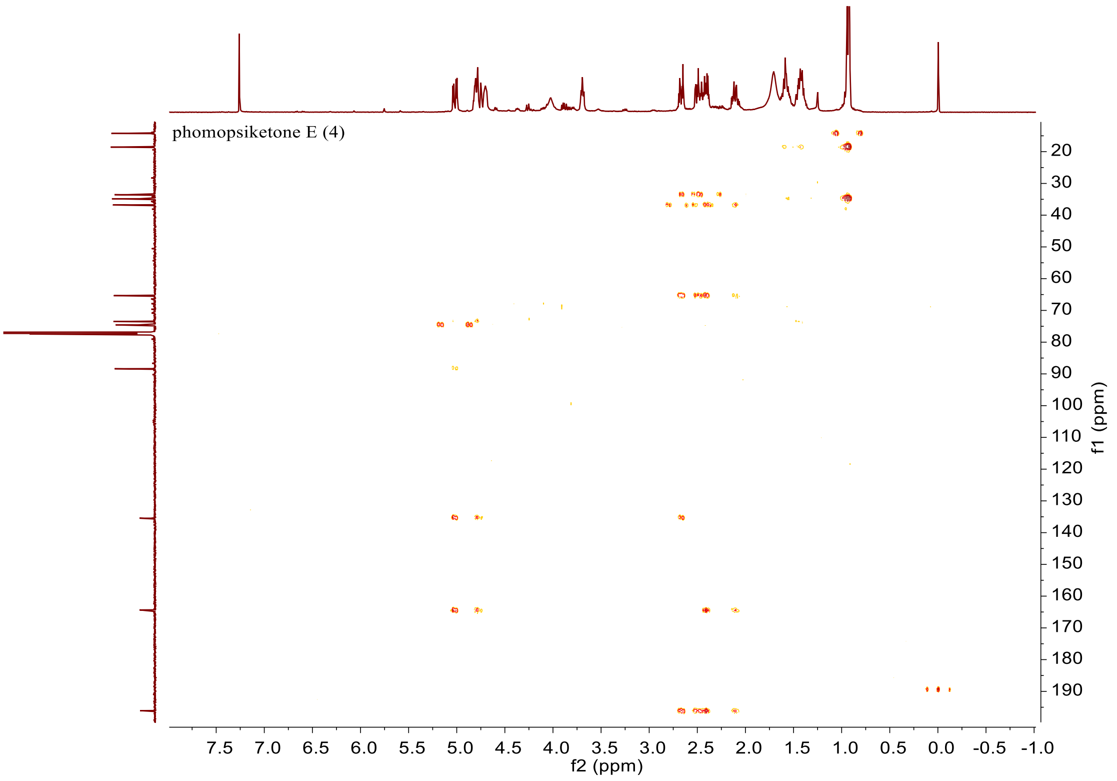

Figure S49. HMBC ($CDCl_3$) spectrum of phomopsiketone G (**4**)

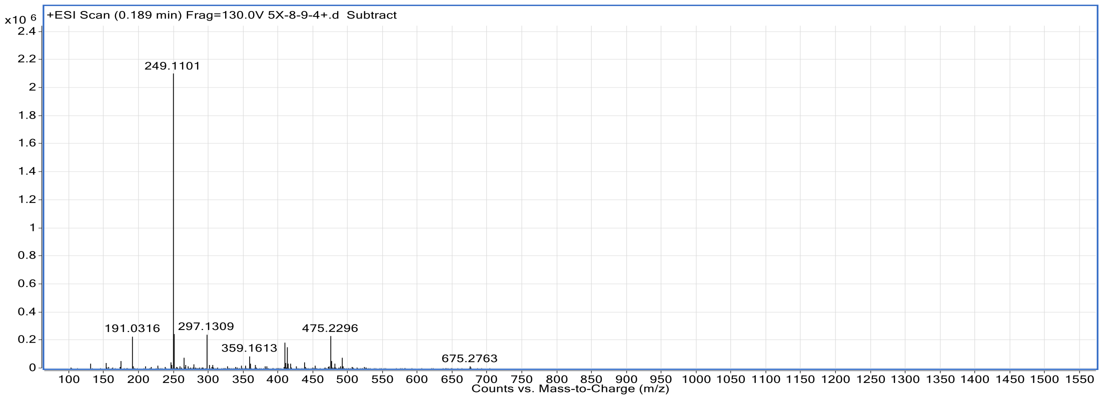

Figure S50. HRESIMS spectrum of phomopsiketone G (**4**)

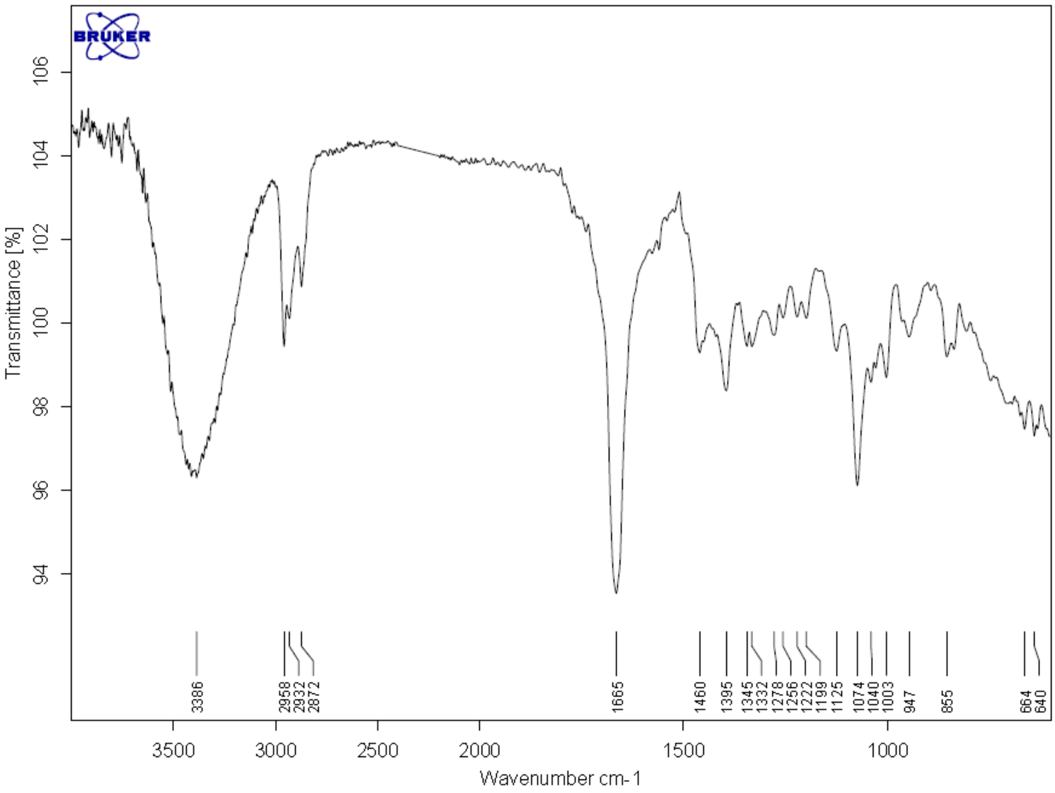

Figure S51. IR spectrum of phomopsiketone G (**4**)

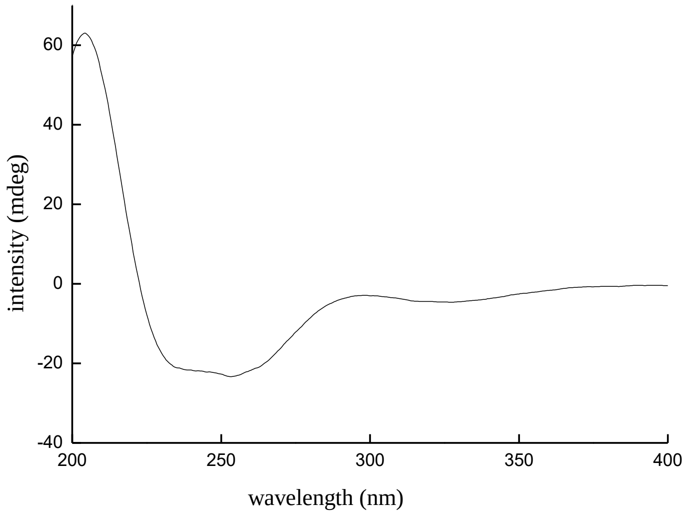

Figure S52. CD spectrum of phomopsiketone G (**4**)

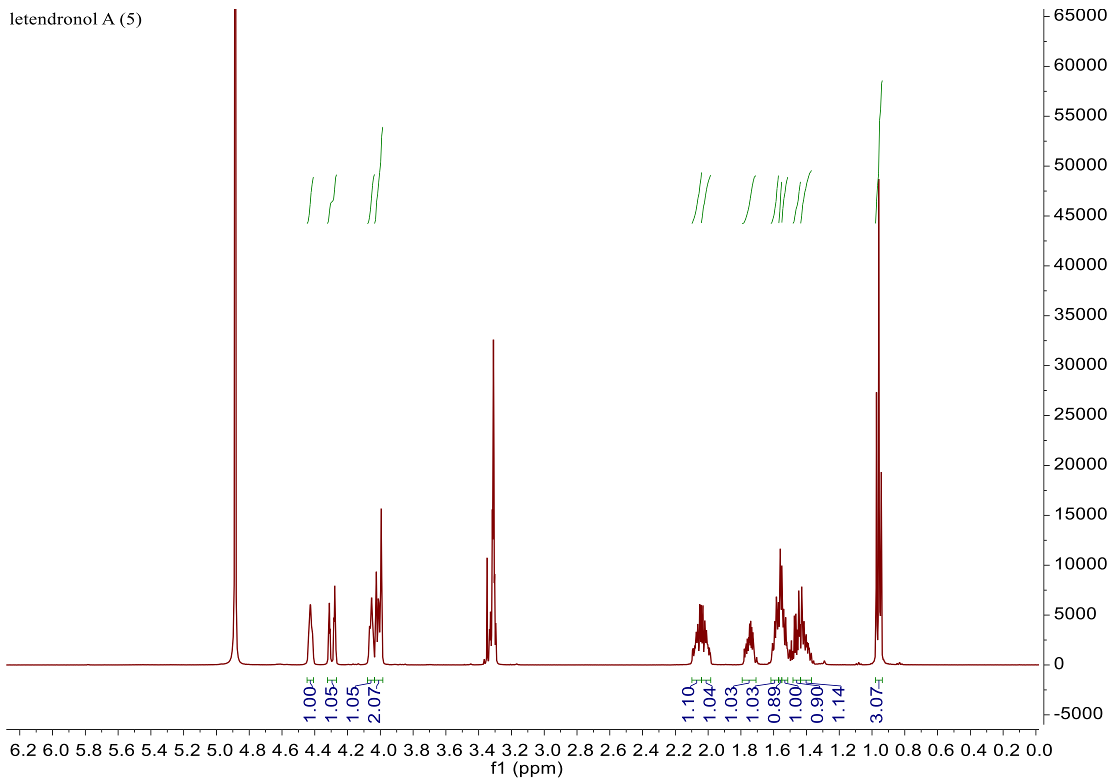

Figure S53. ^{1}H NMR (500 MHz, CD_3OD) spectrum of letendronol A (**5**)

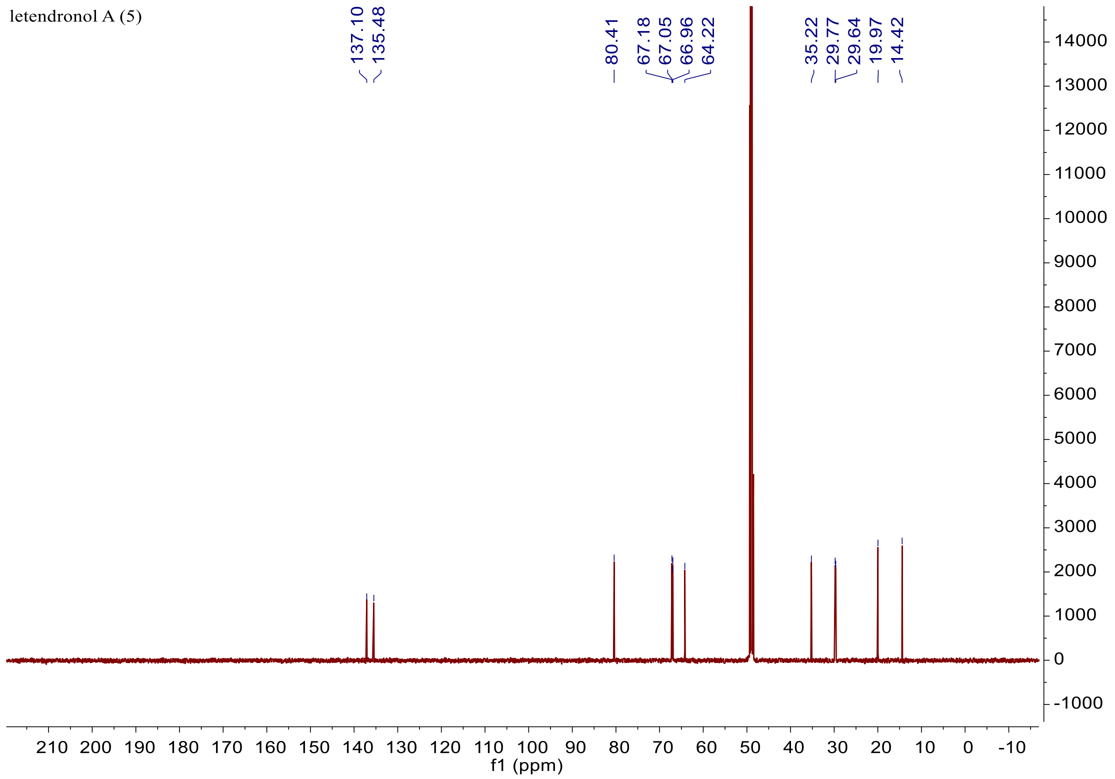

Figure S54. ^{13}C NMR (125 MHz, CD_3OD) spectrum of letendronol A (**5**)

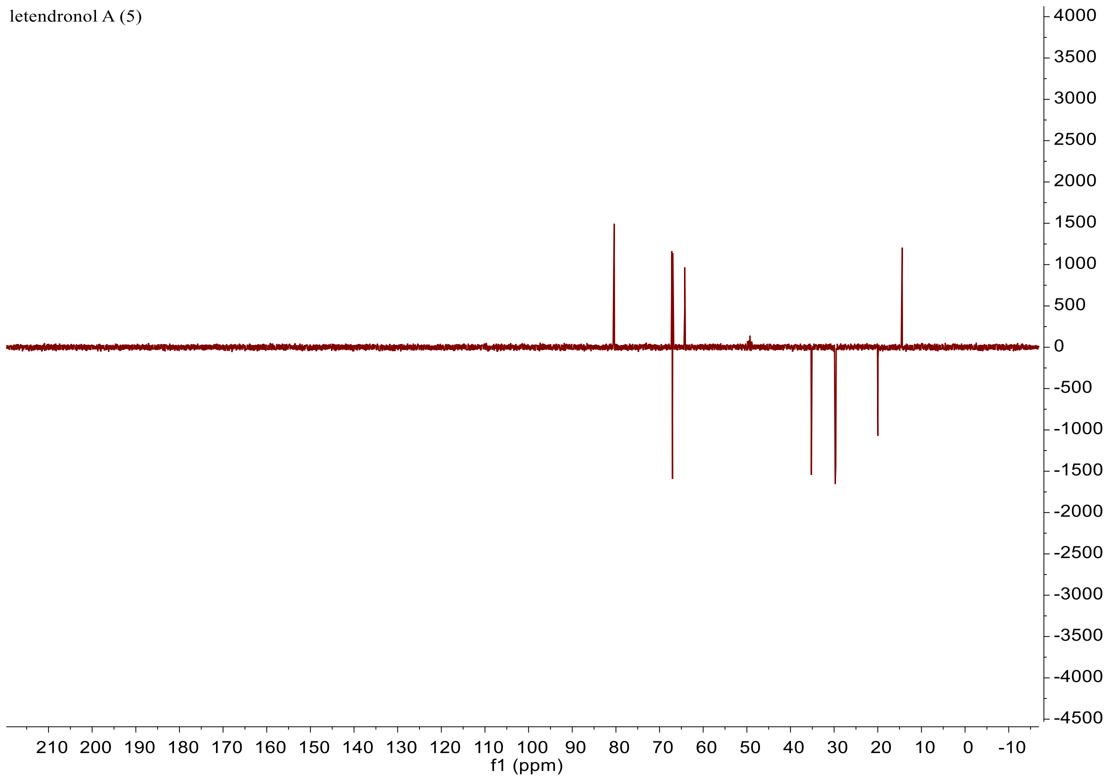

Figure S55. DEPT (CD_3OD) spectrum of letendronol A (**5**)

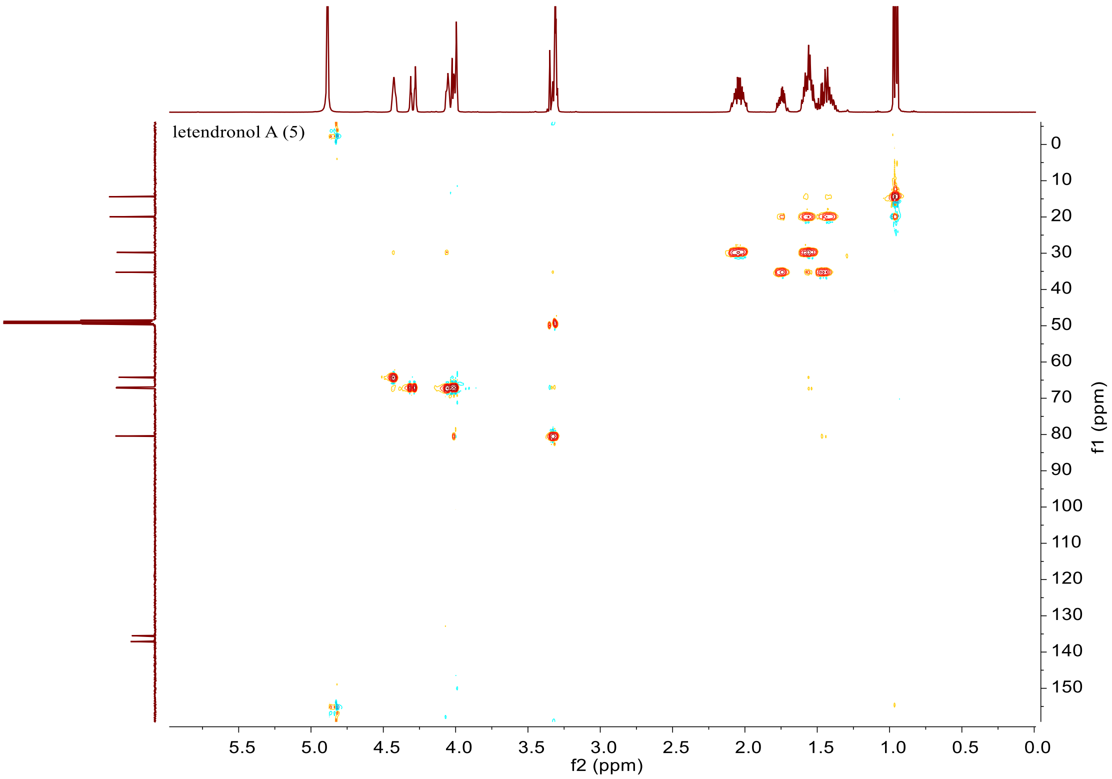

Figure S56. HSQC (CD_3OD) spectrum of letendronol A (**5**)

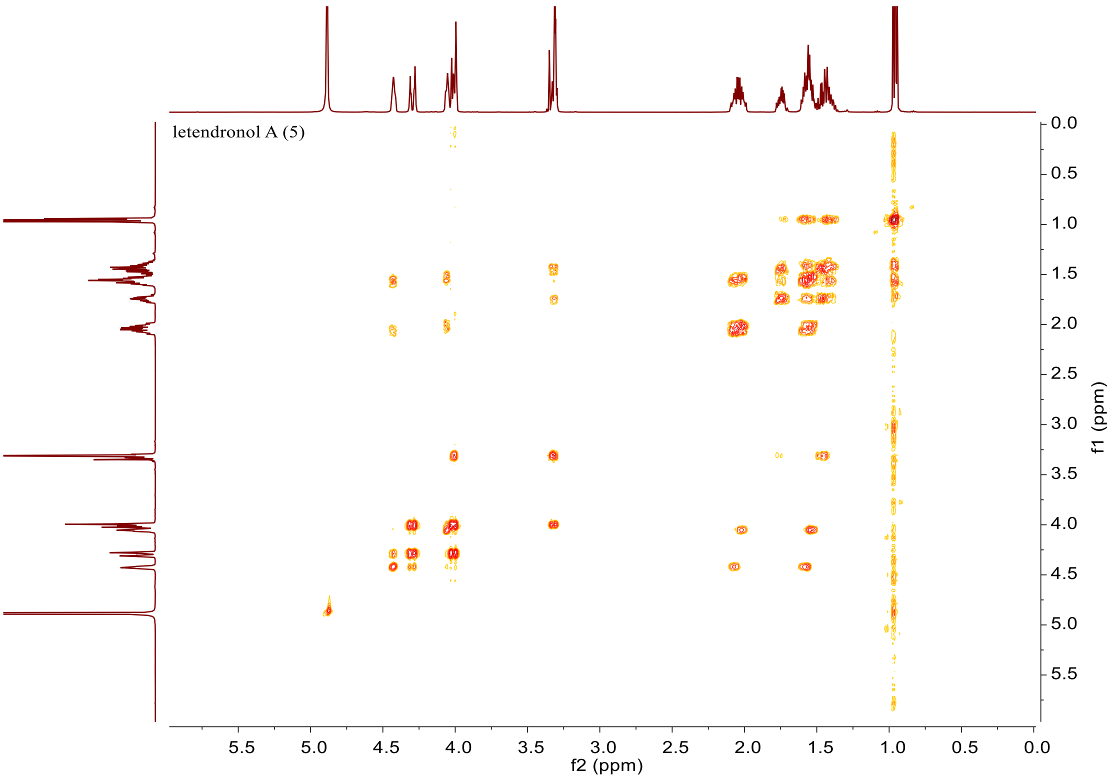

Figure S57. ^{1}H–^{1}H COSY (CD_3OD) spectrum of letendronol A (**5**)

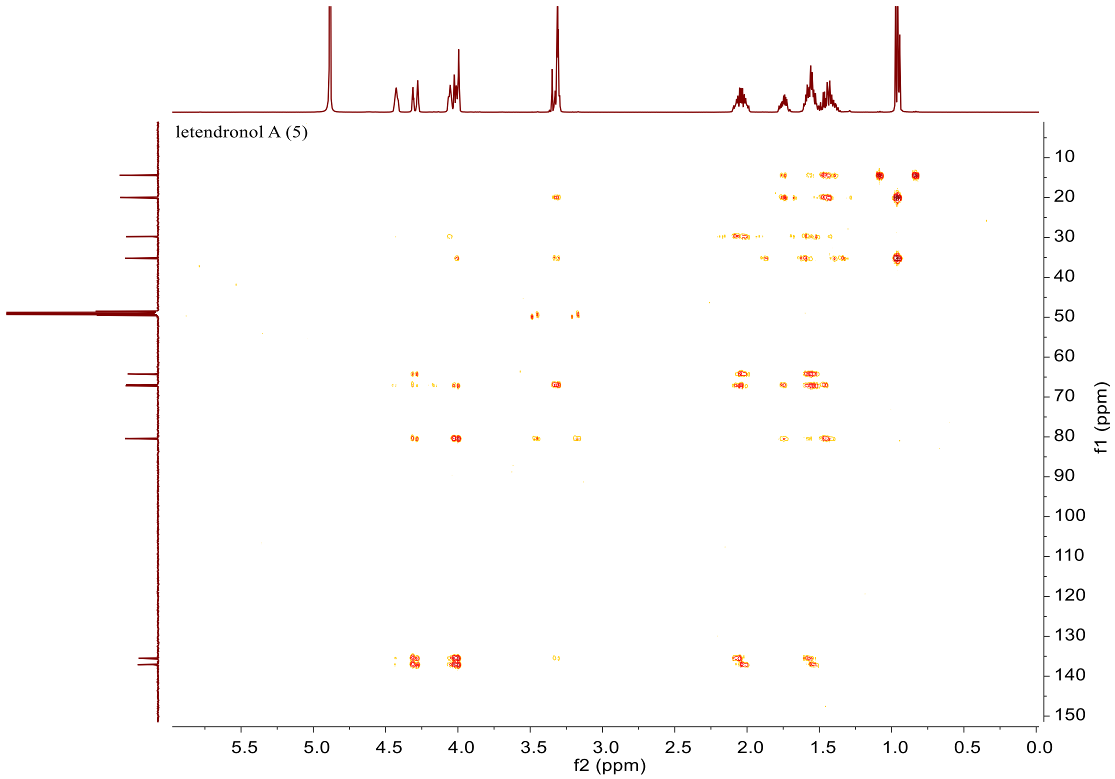

Figure S58. HMBC (CD_3OD) spectrum of letendronol A (**5**)

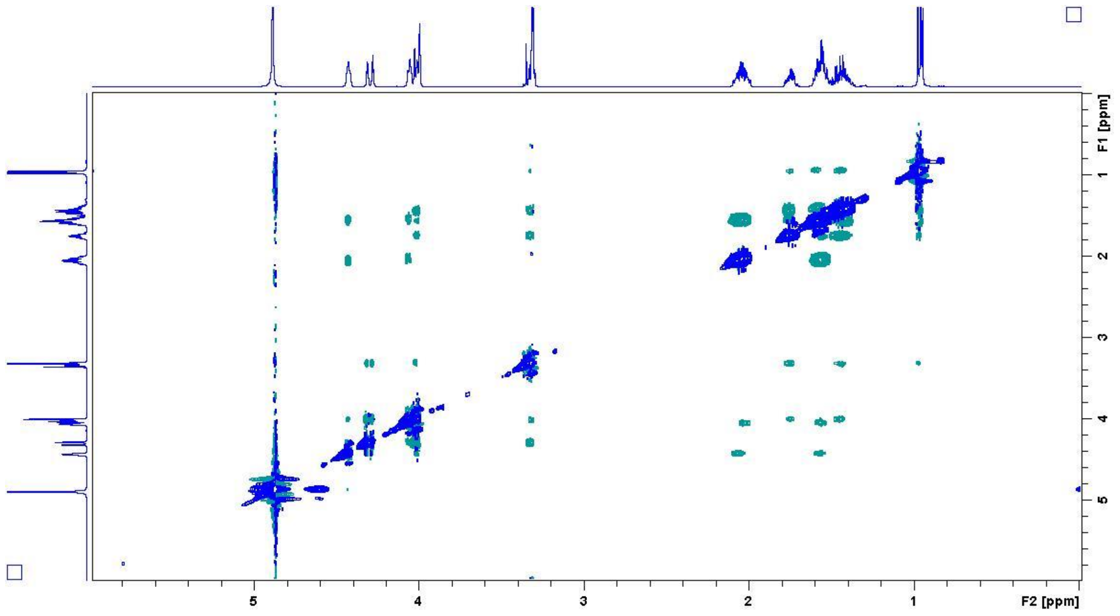

Figure S59. NOESY (CD_3OD) spectrum of letendronol A (**5**)

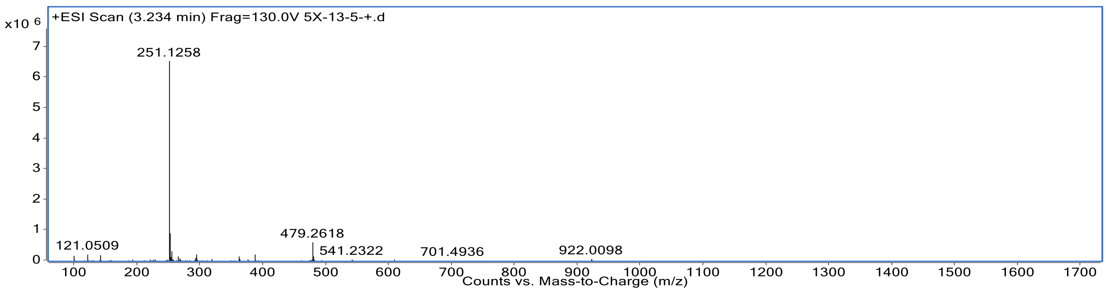

Figure S60. HRESIMS spectrum of letendronol A (**5**)

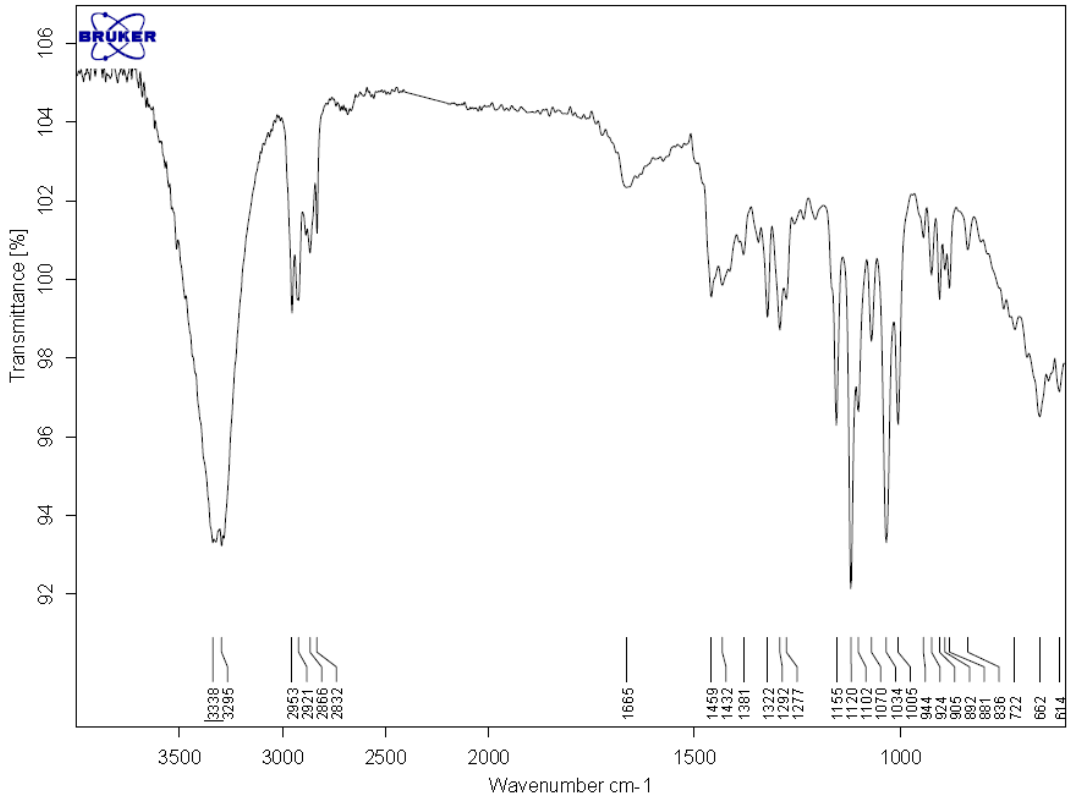

Figure S61. IR spectrum of letendronol A (**5**)

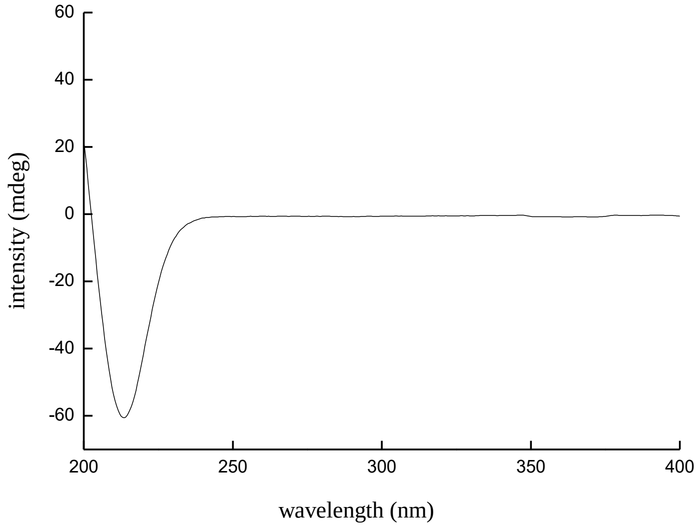

Figure S62. CD spectrum of letendronol A (**5**)

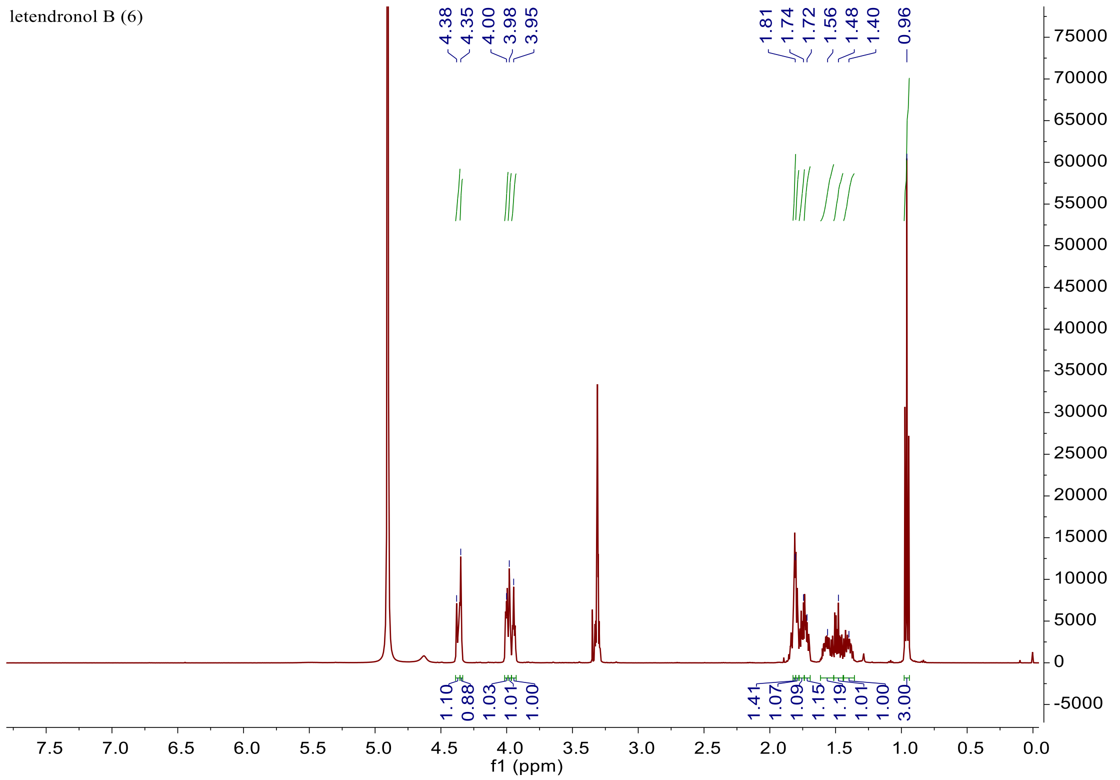

Figure S63. ^{1}H NMR (500 MHz, CD_3OD) spectrum of letendronol B (**6**)

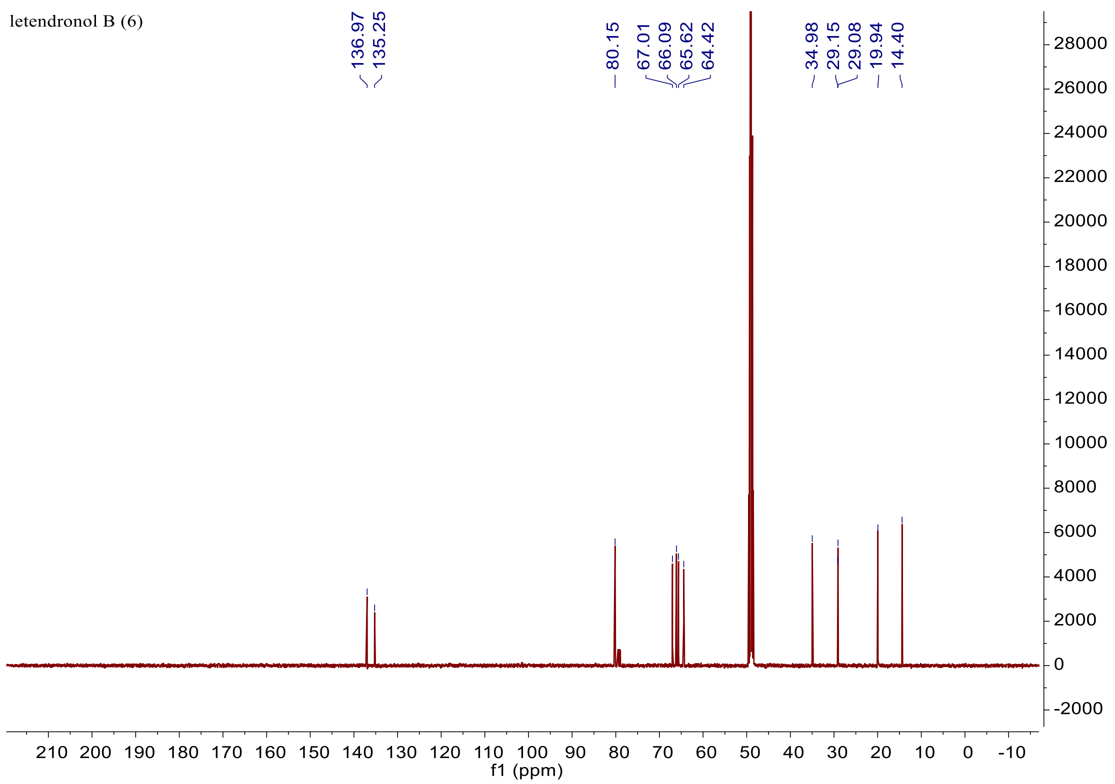

Figure S64. ^{13}C NMR (125 MHz, CD_3OD) spectrum of letendronol B (**6**)

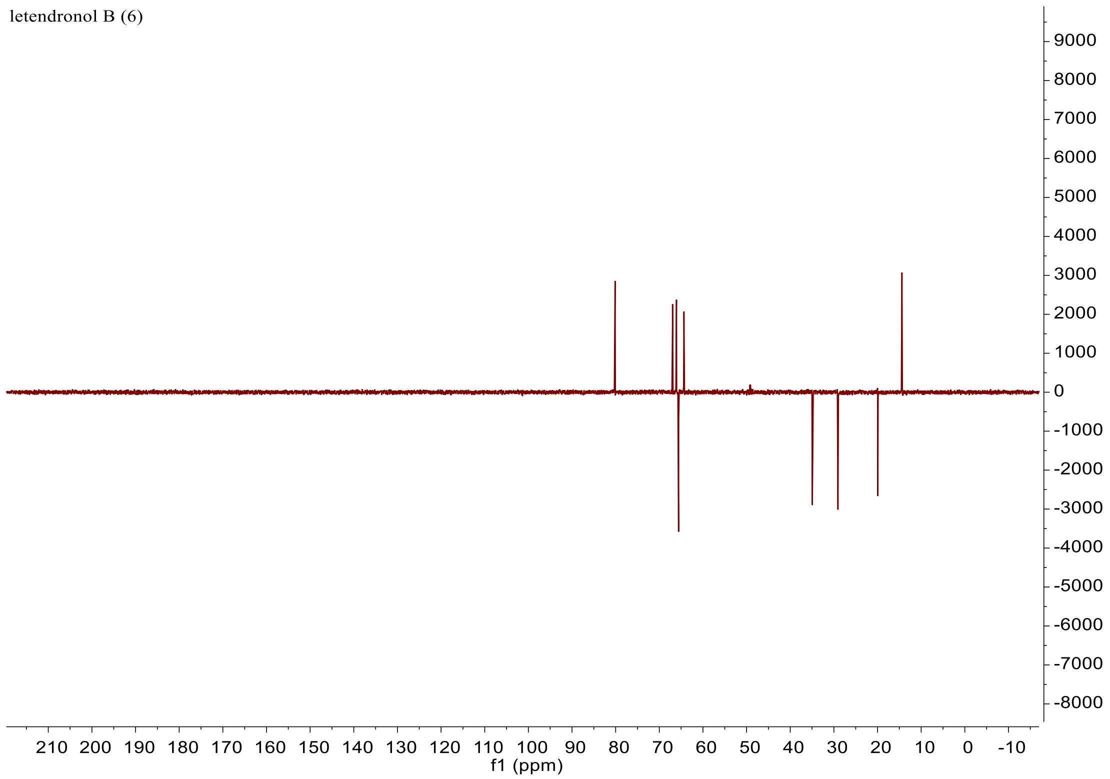

Figure S65. DEPT (CD_3OD) spectrum of letendronol B (**6**)

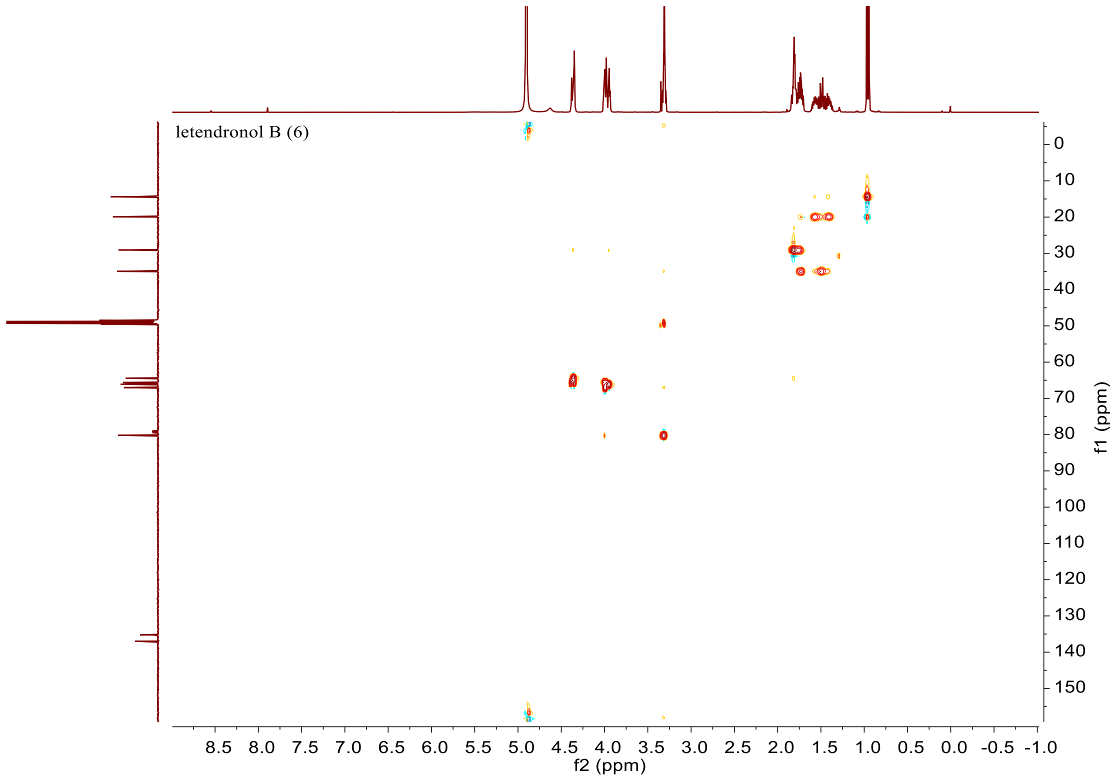

Figure S66. HSQC (CD_3OD) spectrum of letendronol B (**6**)

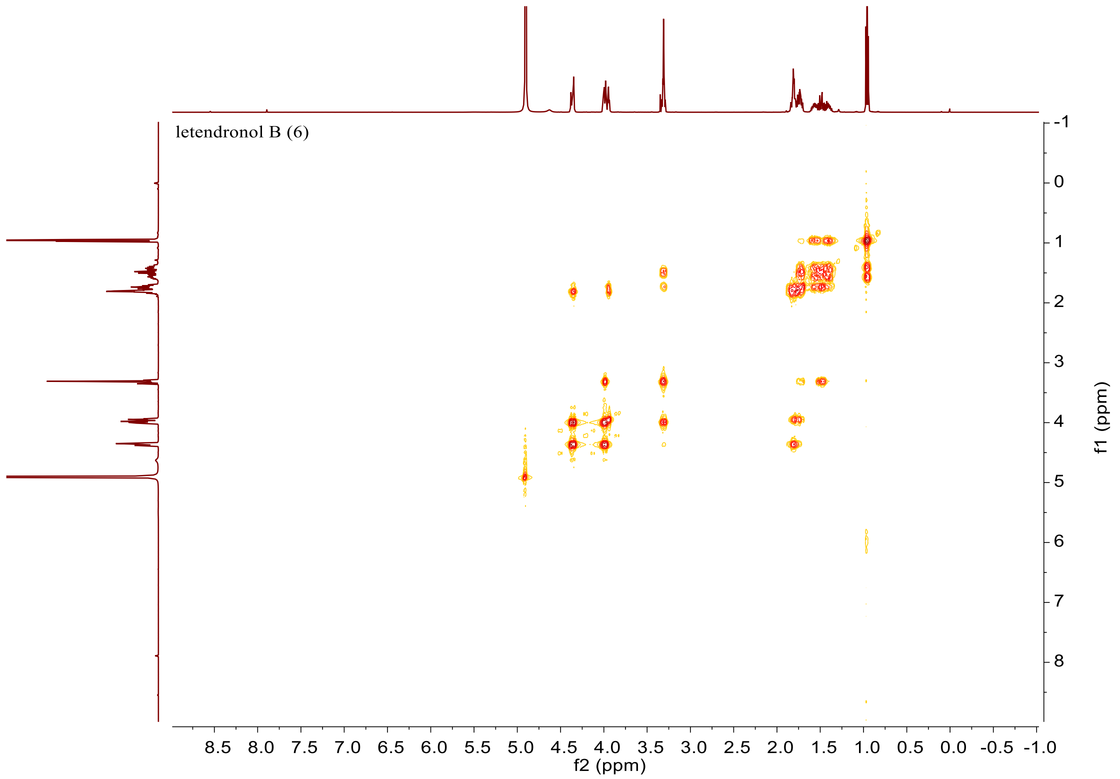

Figure S67. ^{1}H–^{1}H COSY (CD_3OD) spectrum of letendronol B (**6**)

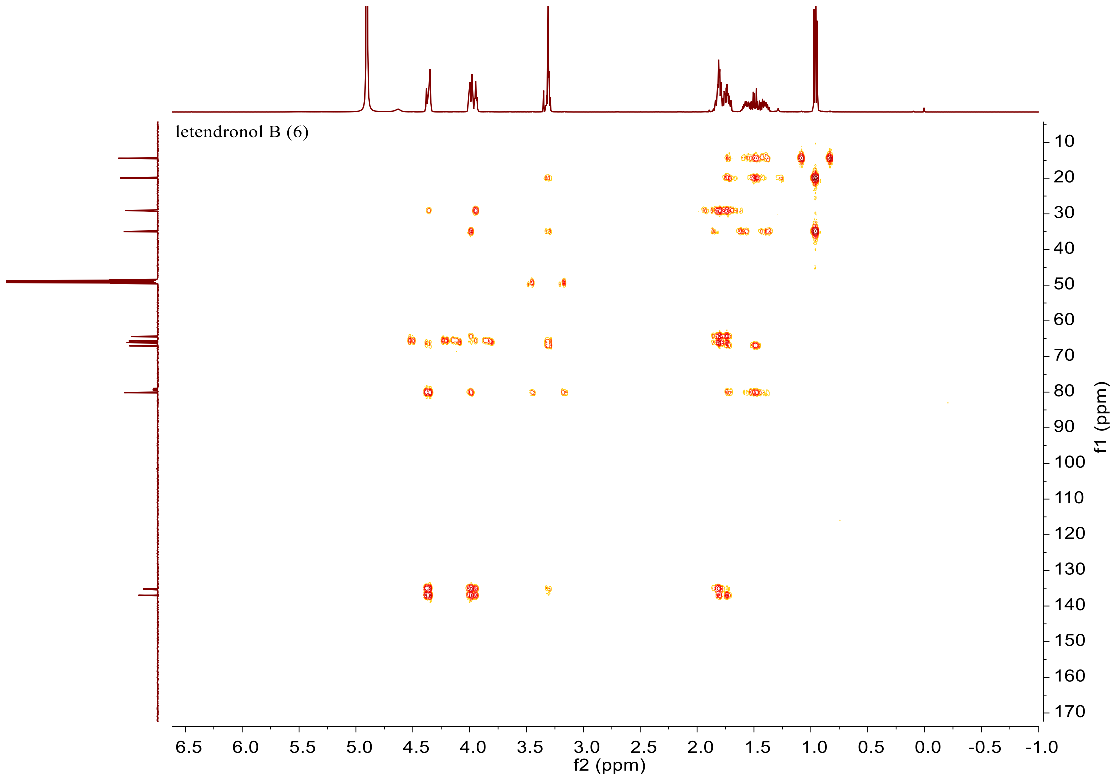

Figure S68. HMBC (CD_3OD) spectrum of letendronol B (**6**)

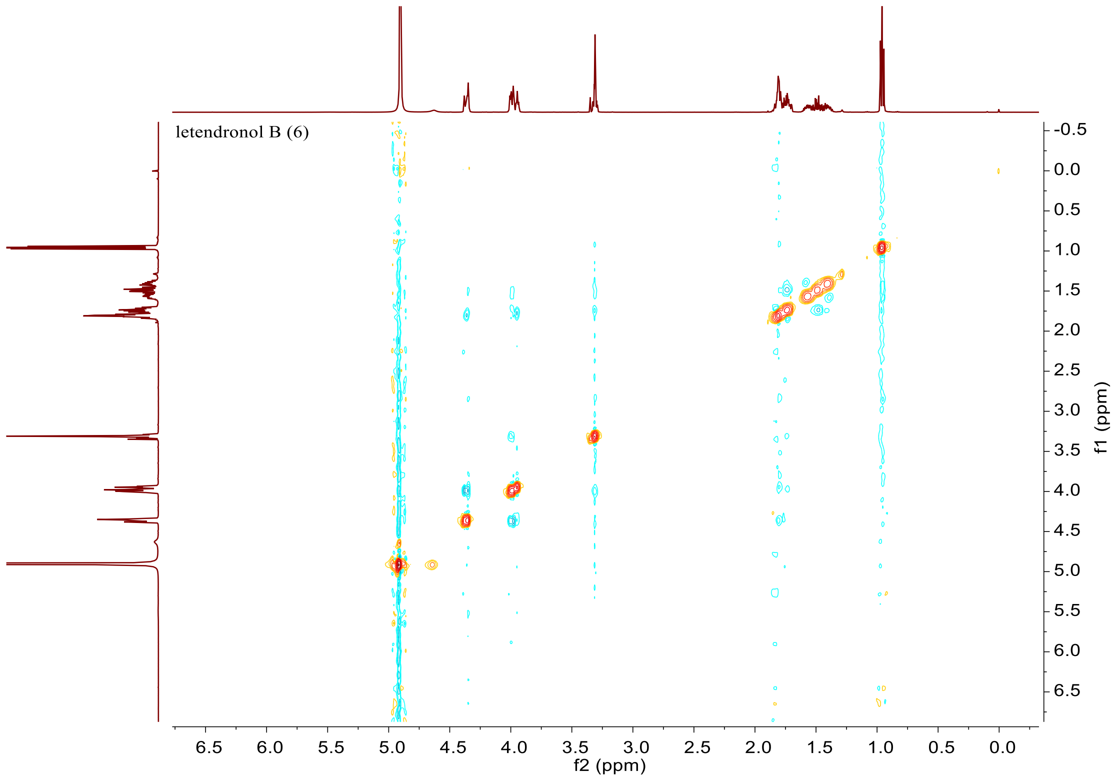

Figure S69. NOESY (CD_3OD) spectrum of letendronol B (**6**)

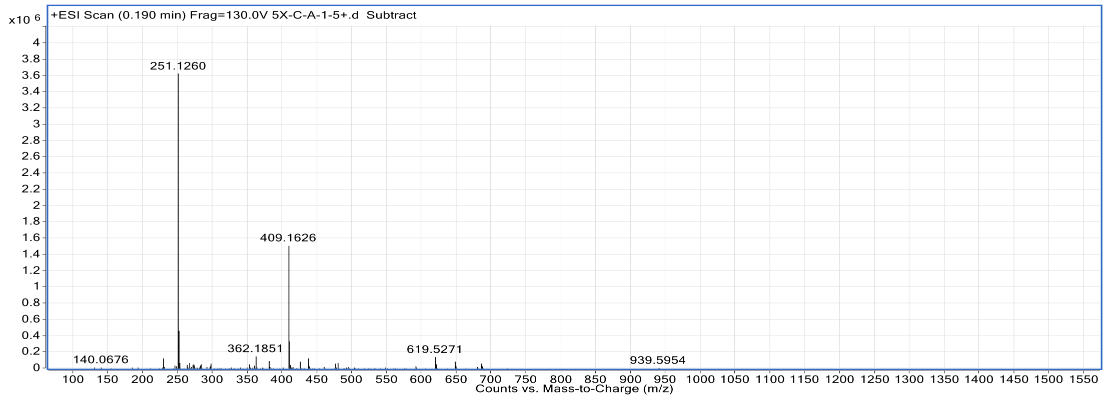

Figure S70. HRESIMS spectrum of letendronol B (**6**)

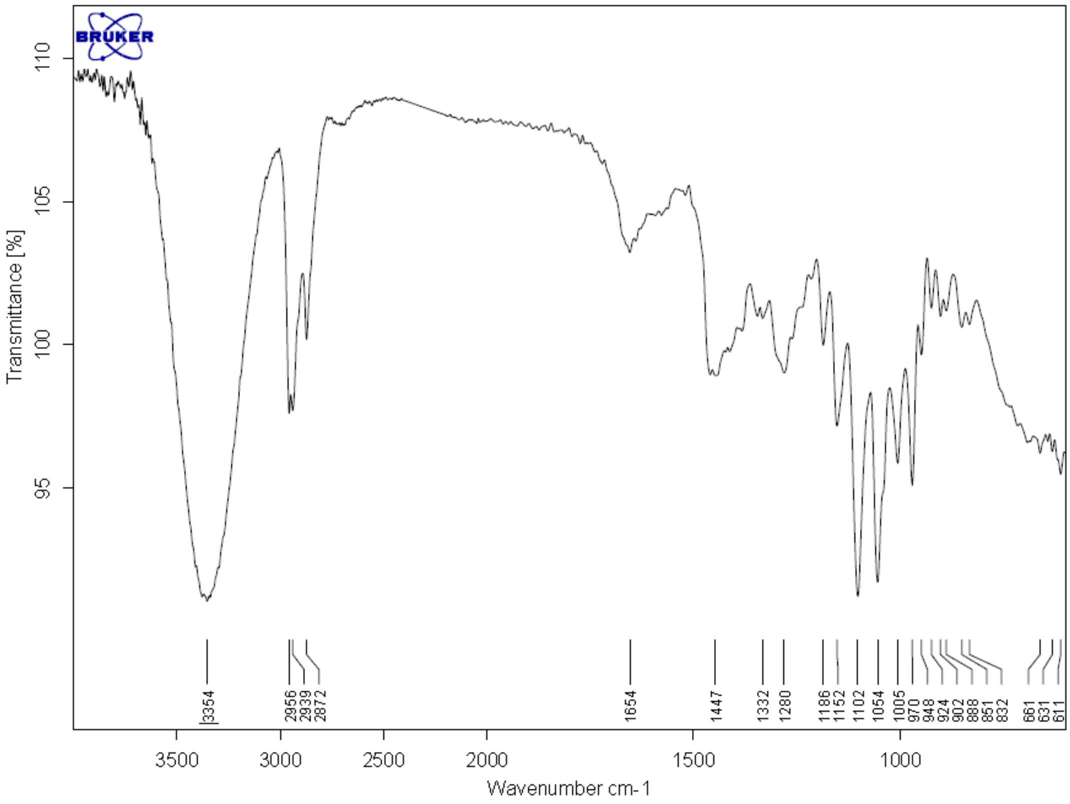

Figure S71. IR spectrum of letendronol B (**6**)

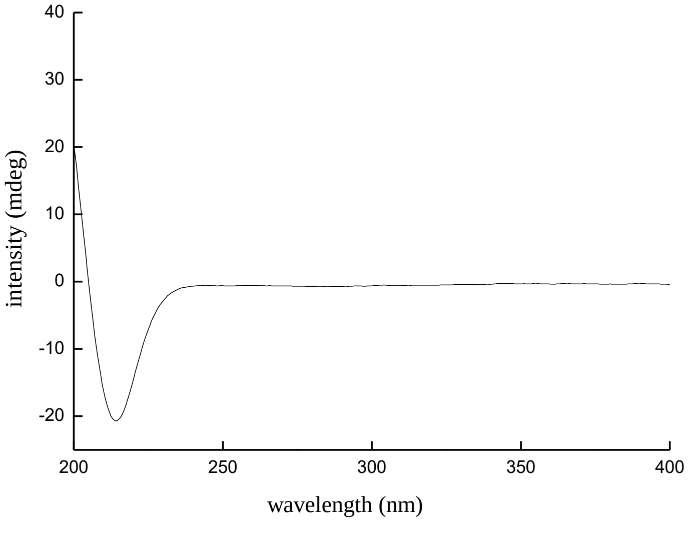

Figure S72. CD spectrum of letendronol B (**6**)

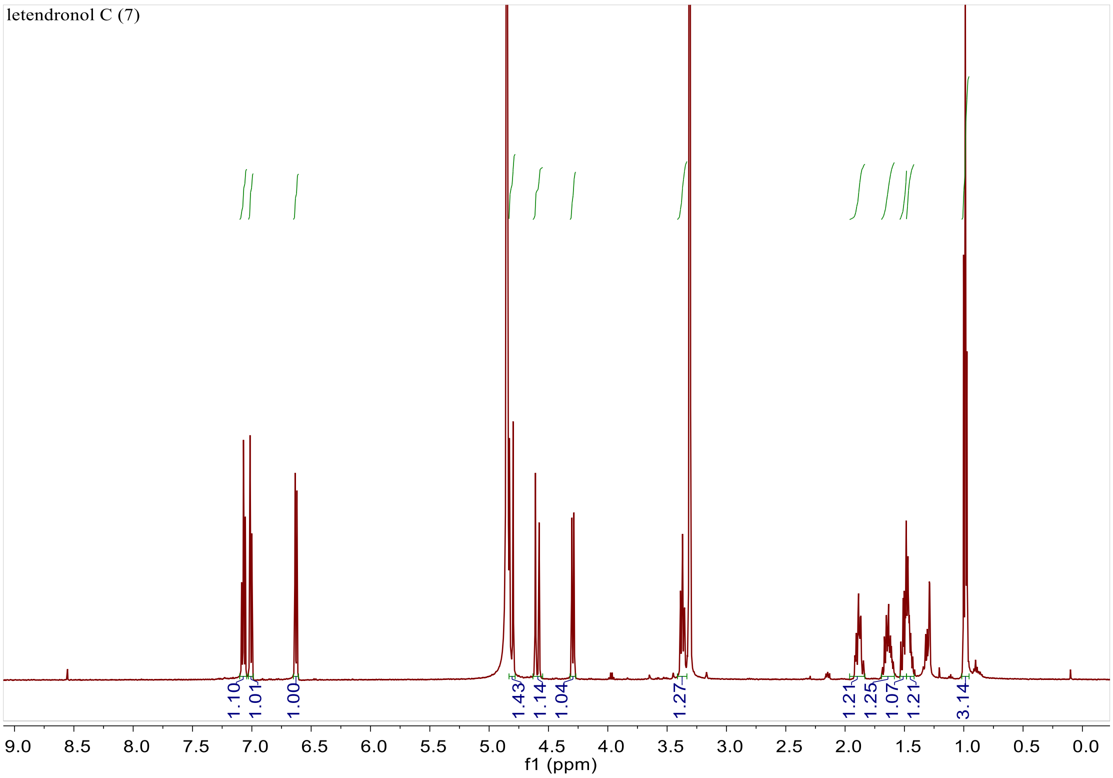

Figure S73. ^{1}H NMR (500 MHz, CD_3OD) spectrum of letendronol C (**7**)

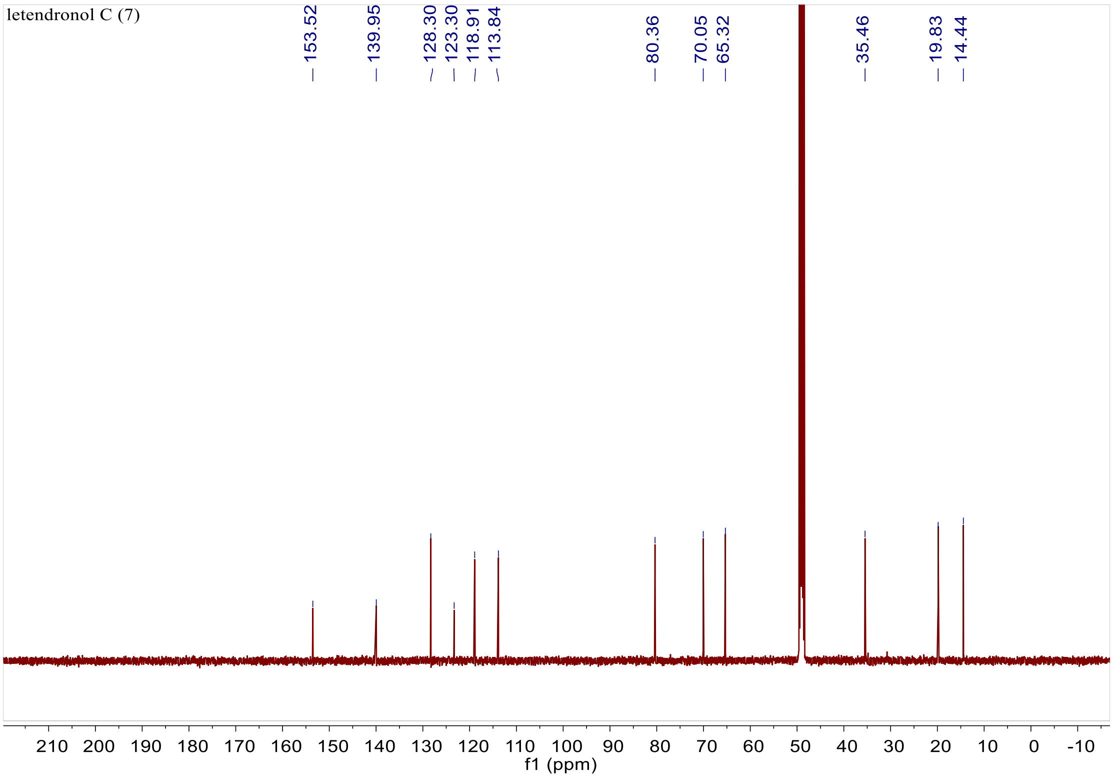

Figure S74. ^{13}C NMR (125 MHz, CD_3OD) spectrum of letendronol C (**7**)

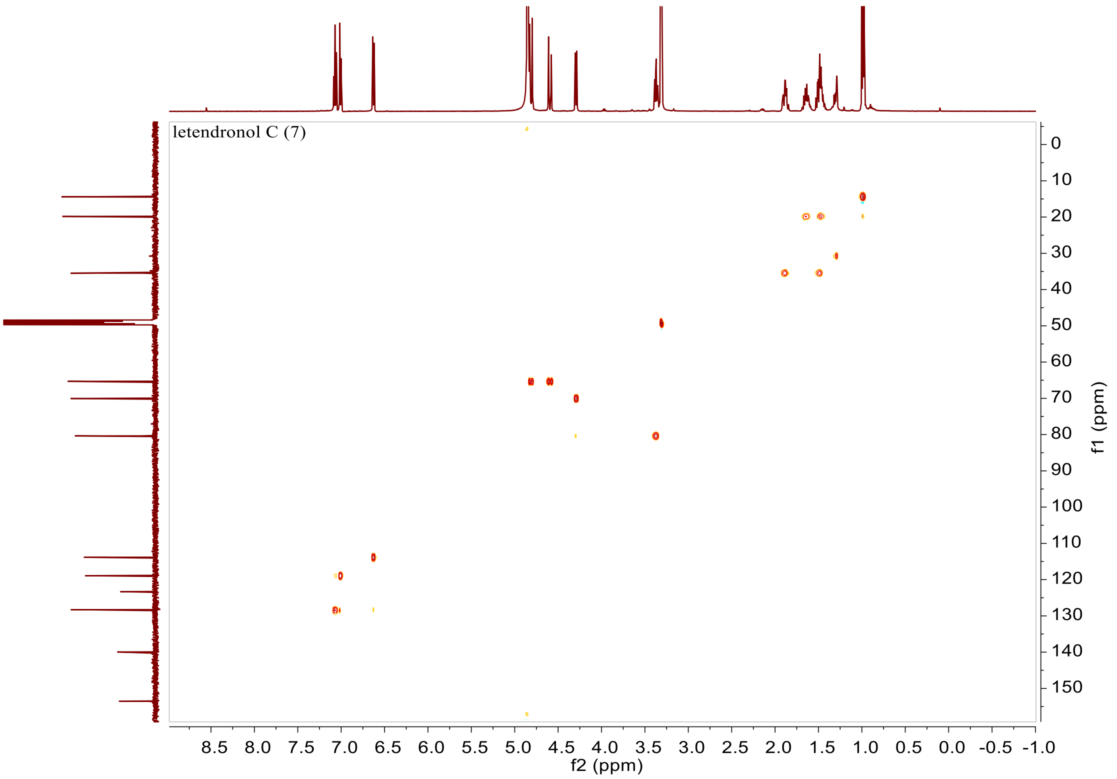

Figure S75. HSQC (CD_3OD) spectrum of letendronol C (**7**)

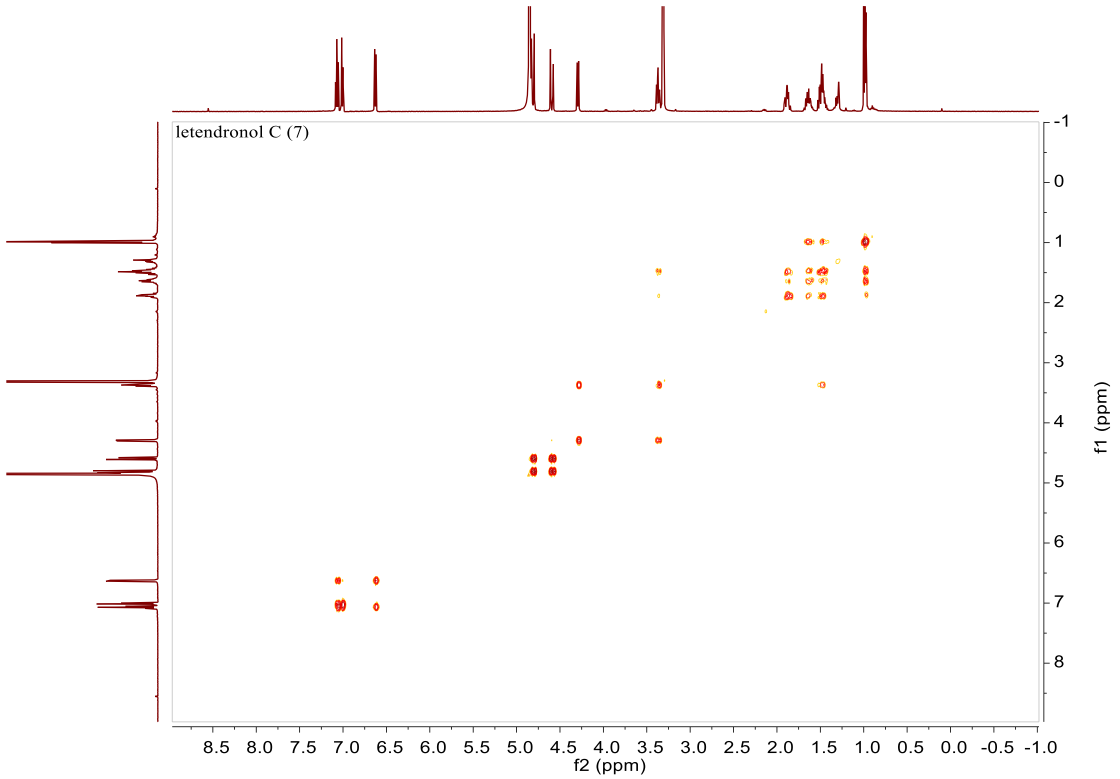

Figure S76. ^1H–^1H COSY (CD_3OD) spectrum of letendronol C (**7**)

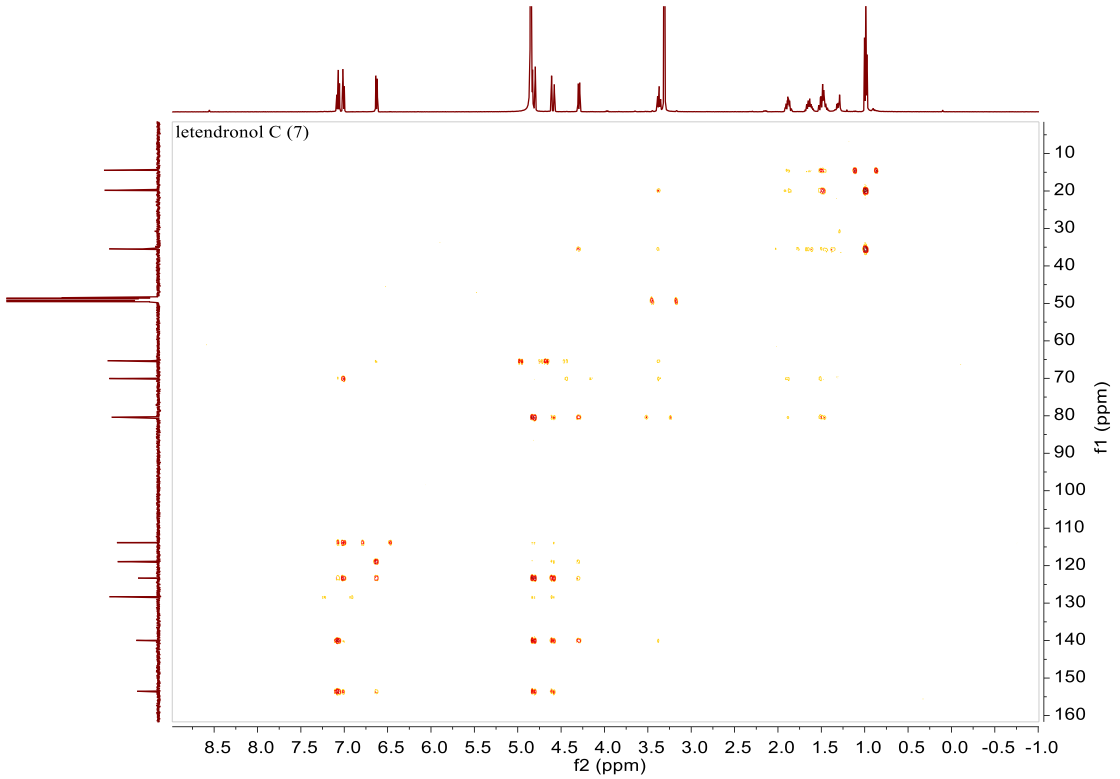

Figure S77. HMBC (CD_3OD) spectrum of letendronol C (**7**)

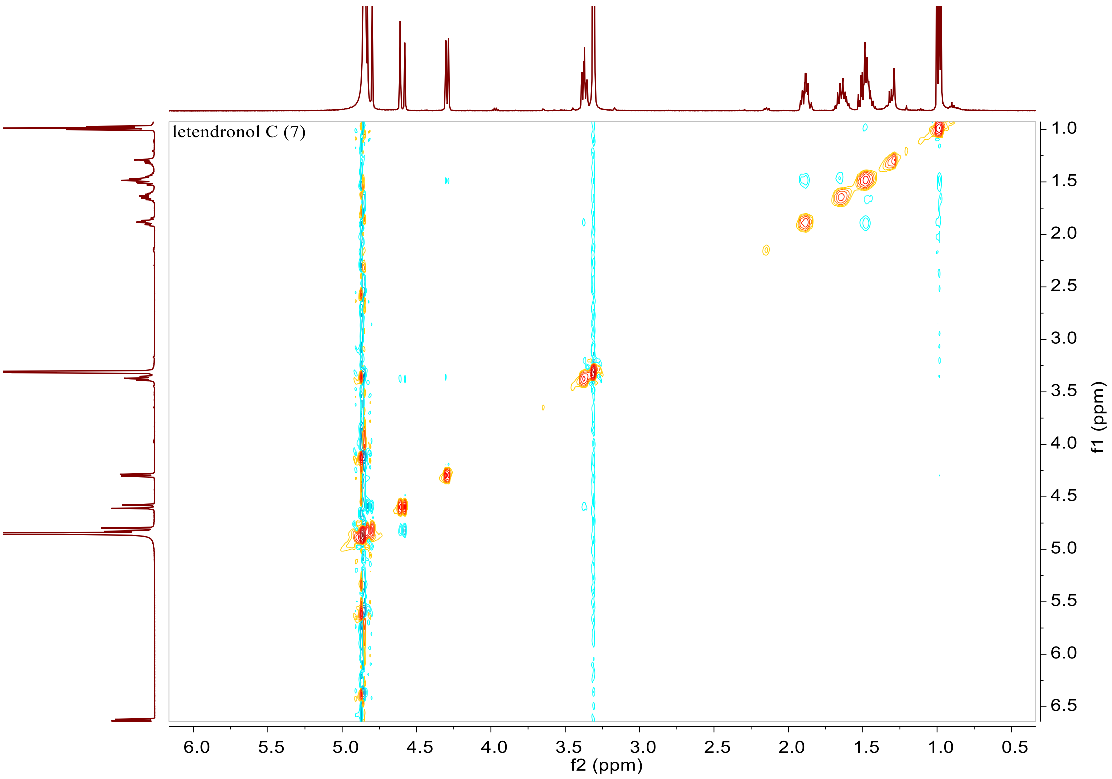

Figure S78. NOESY (CD_3OD) spectrum of letendronol C (**7**)

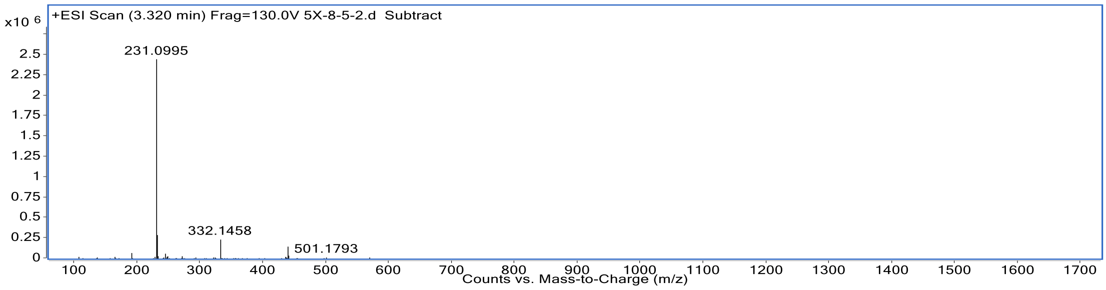

Figure S79. HRESIMS spectrum of letendronol C (**7**)

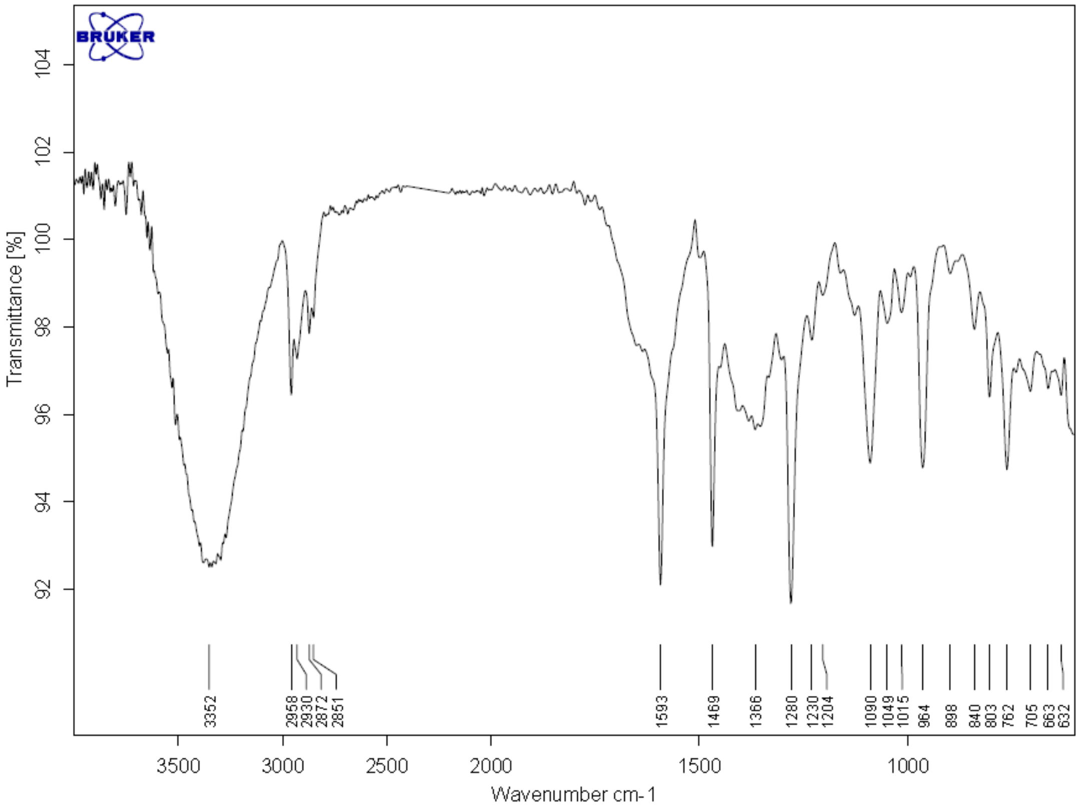

Figure S80. IR spectrum of letendronol C (**7**)

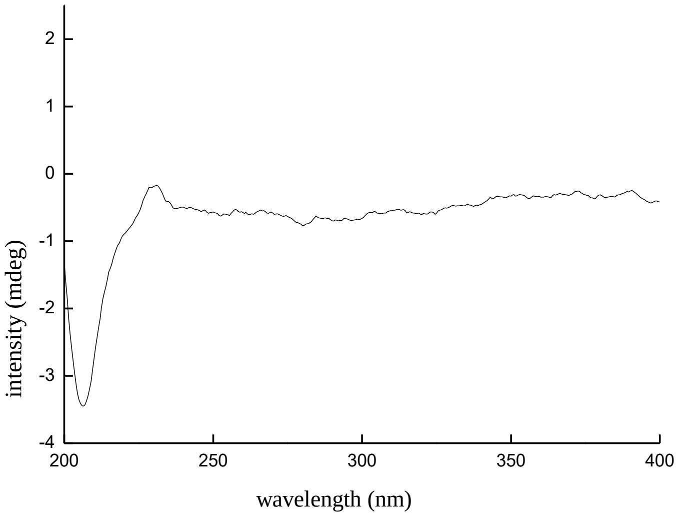

Figure S81. CD spectrum of letendronol C (**7**)

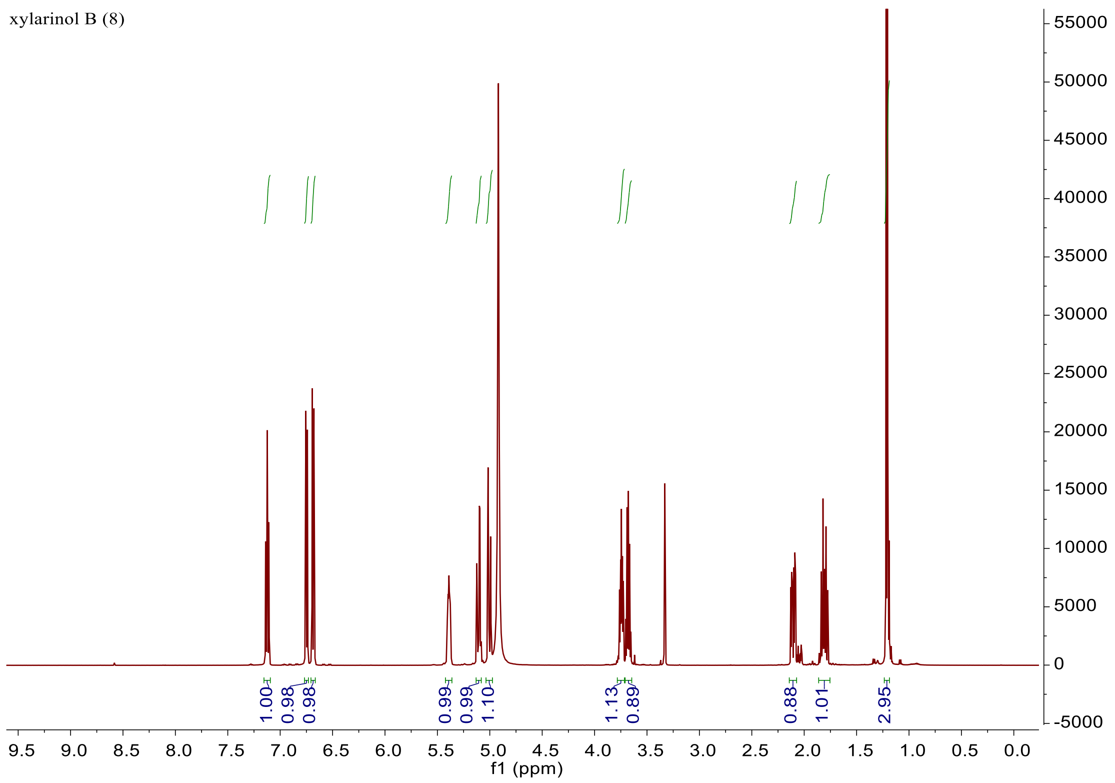

Figure S82. ^{1}H NMR (500 MHz, CD_3OD) spectrum of xylarinol B (**8**)

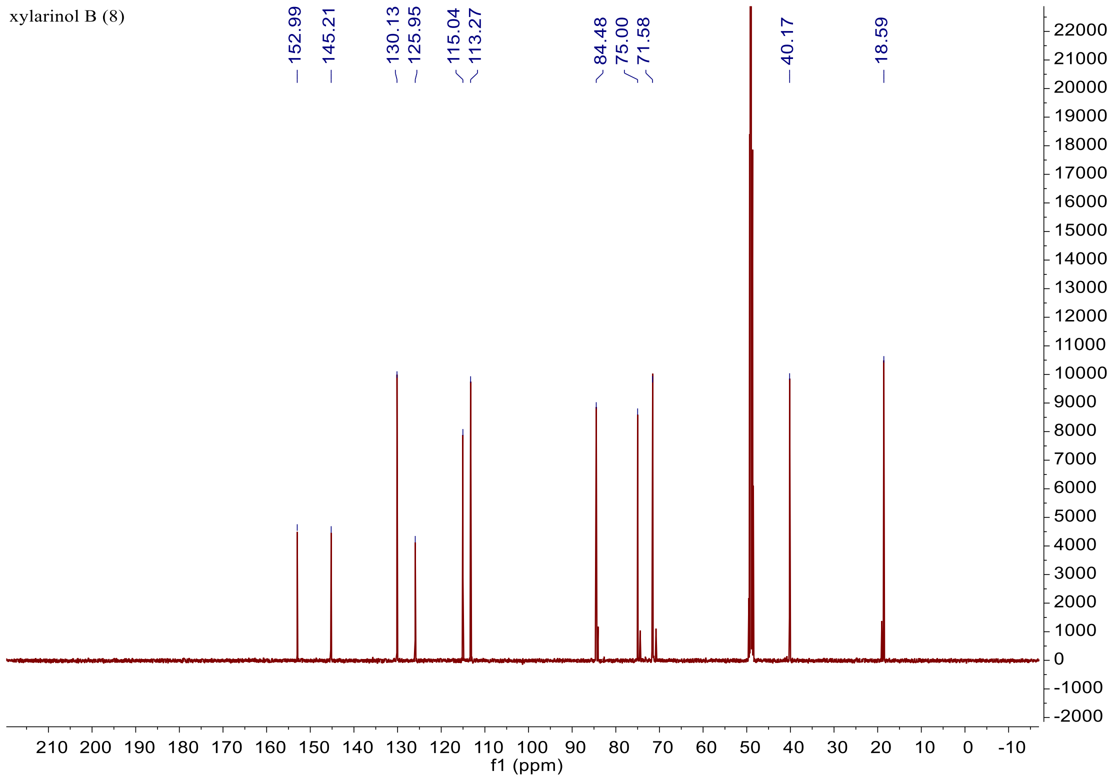

Figure S83. ^{13}C NMR (125 MHz, CD_3OD) spectrum of xylarinol B (**8**)

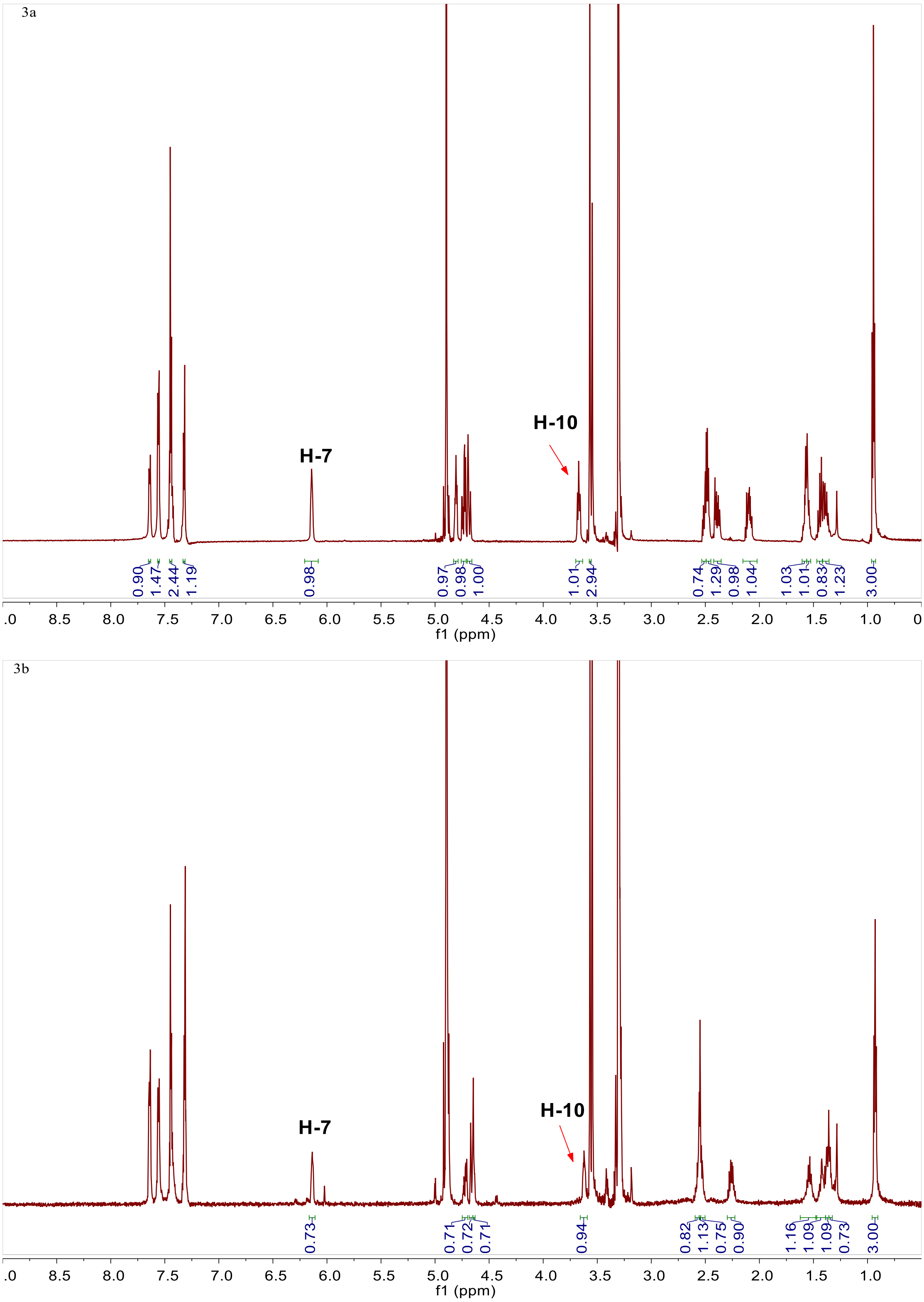

Figure S84. ^{1}H NMR (600 MHz, CD_3OD) spectrum of **3a** and **3b**

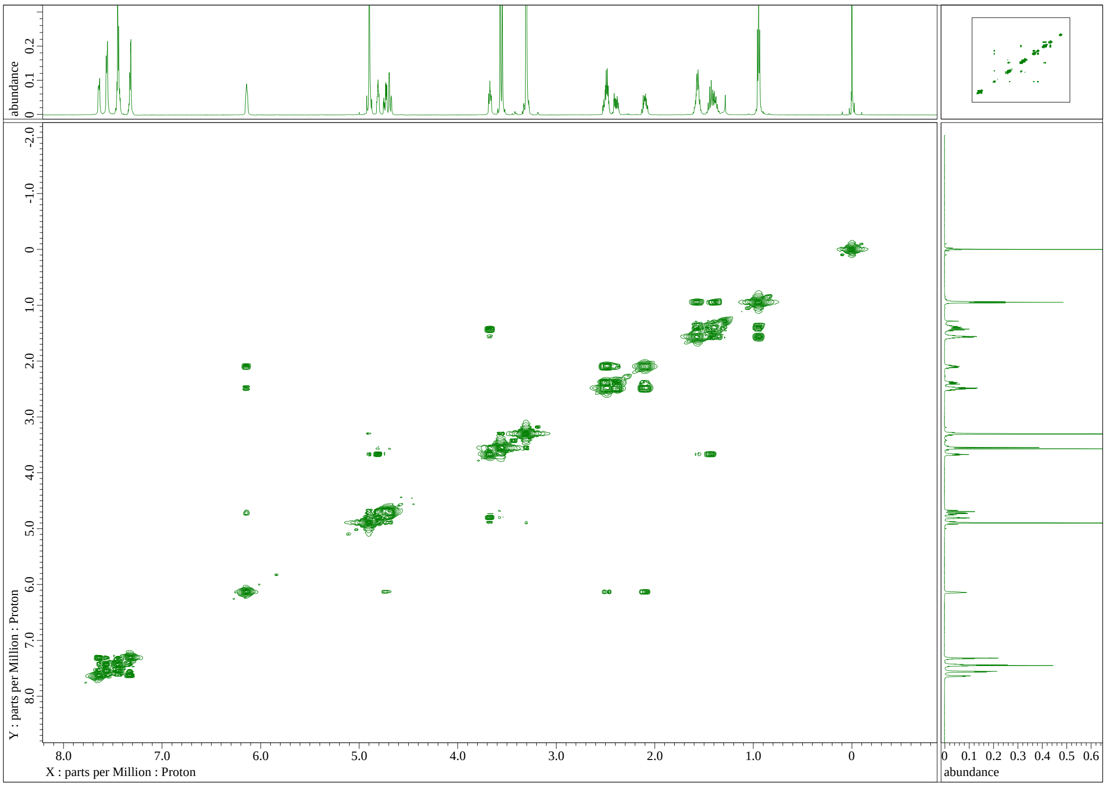

Figure S85. ^1H–^1H COSY (CD_3OD) spectrum of **3a**

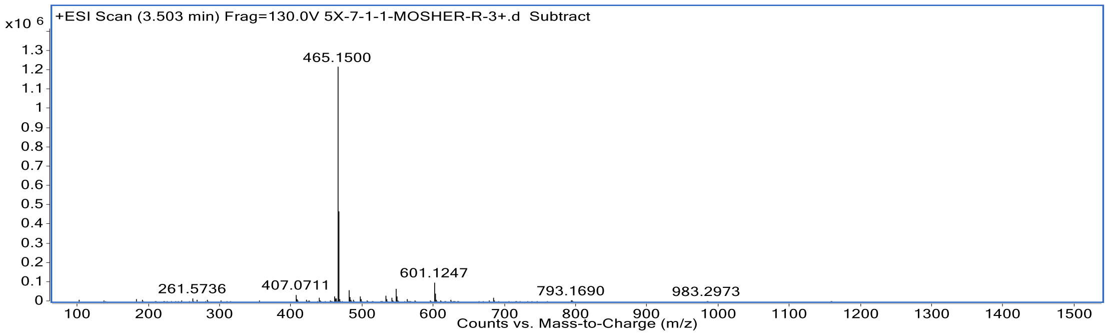

Figure S86. HRESIMS spectrum of **3a**

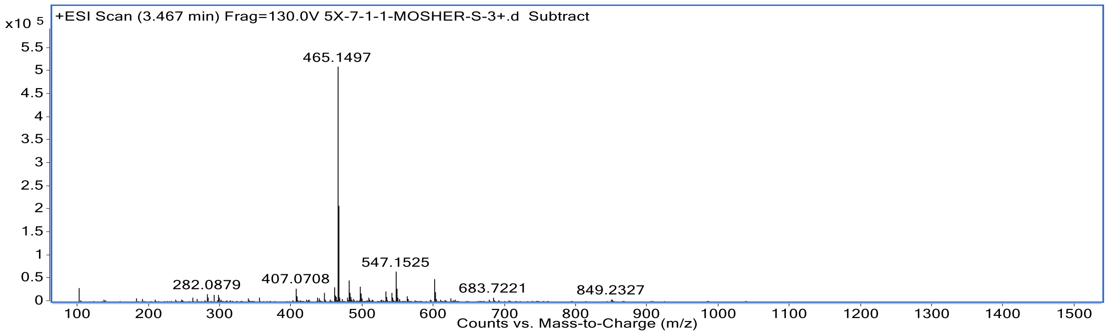

Figure S87. HRESIMS spectrum of **3b**

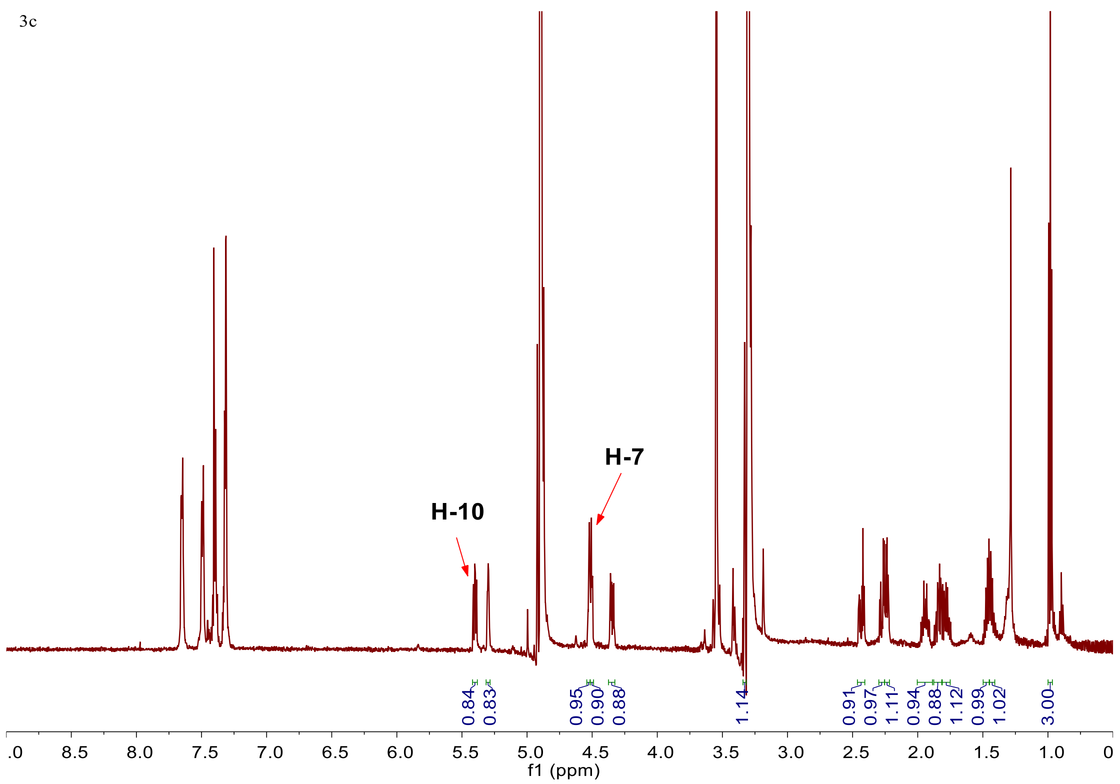

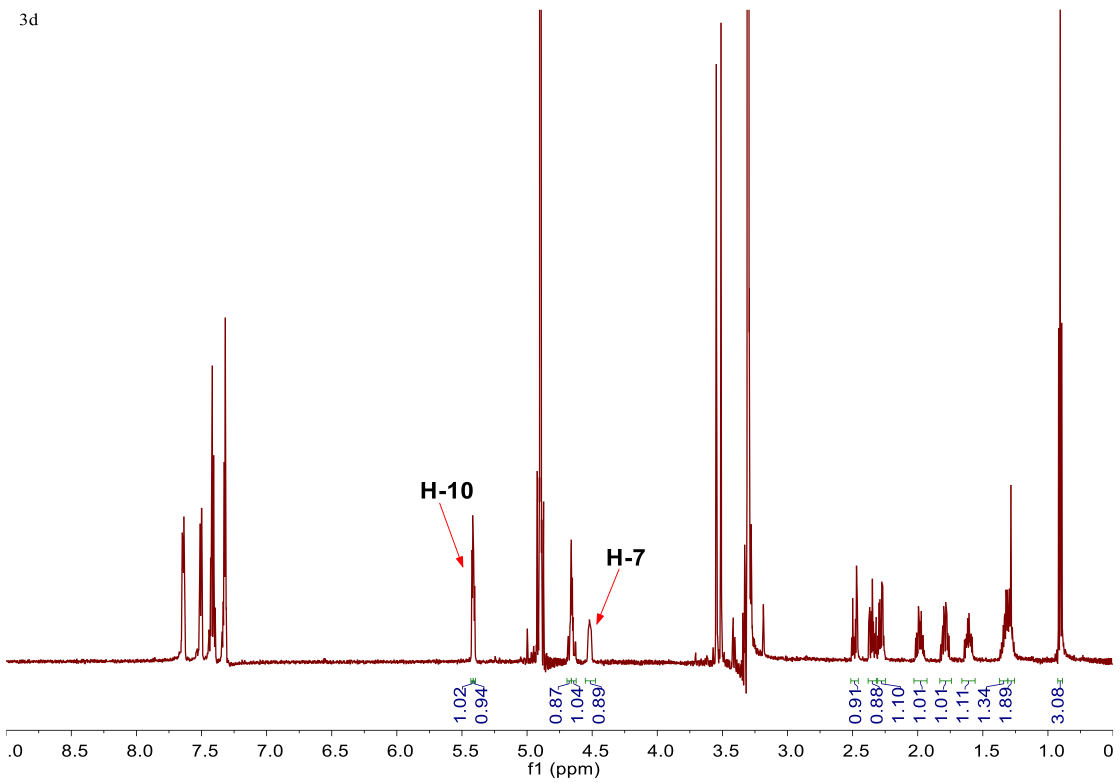

Figure S88. ^{1}H NMR (600 MHz, CD_3OD) spectrum of **3c** and **3d**

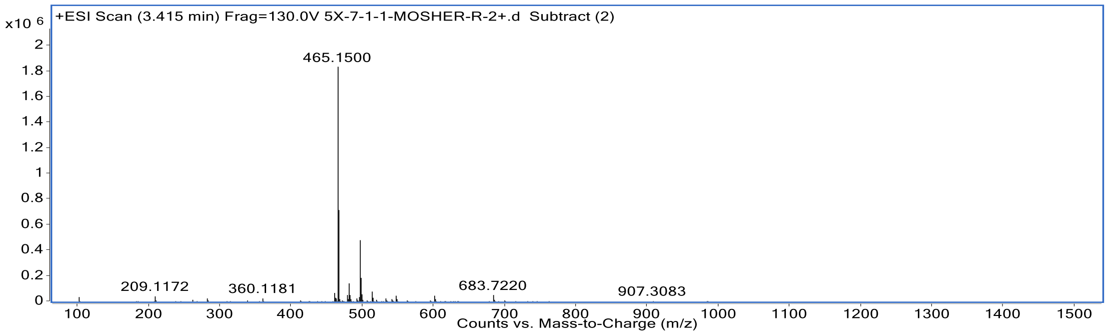

Figure S89. HRESIMS spectrum of **3c**

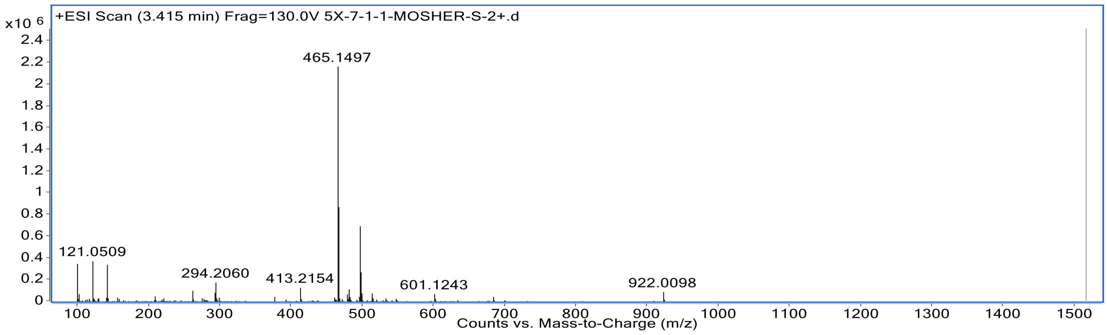

Figure S90. HRESIMS spectrum of **3d**

3e

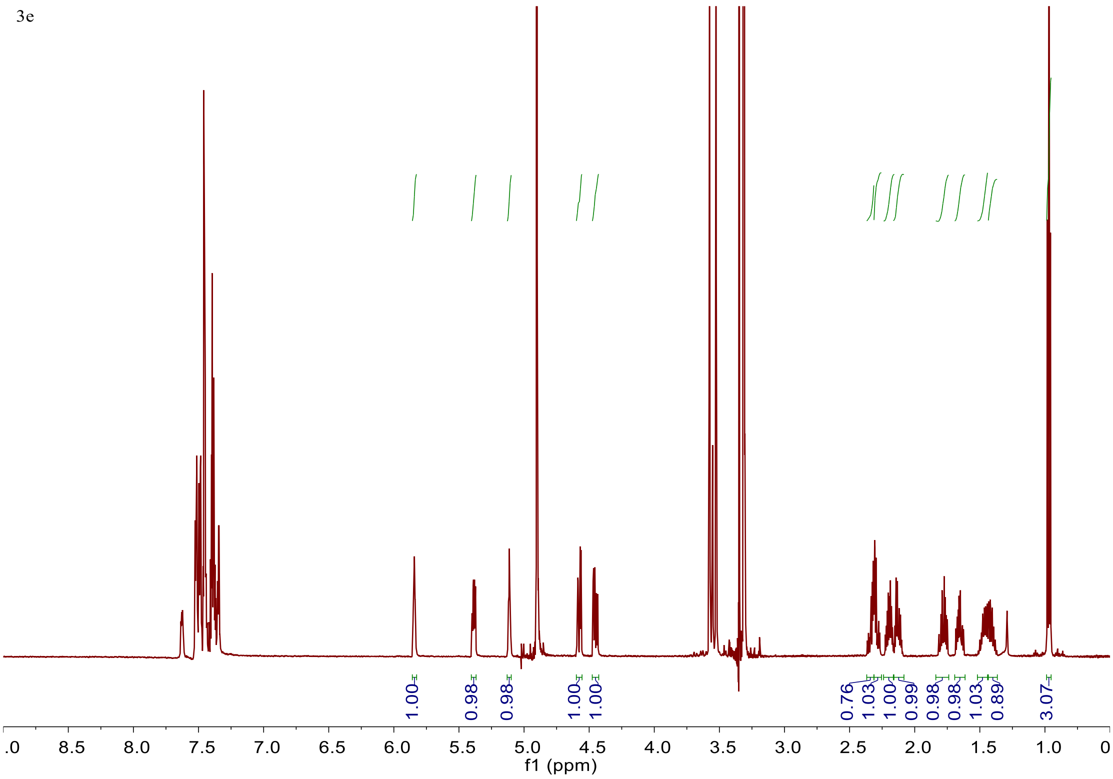

3f

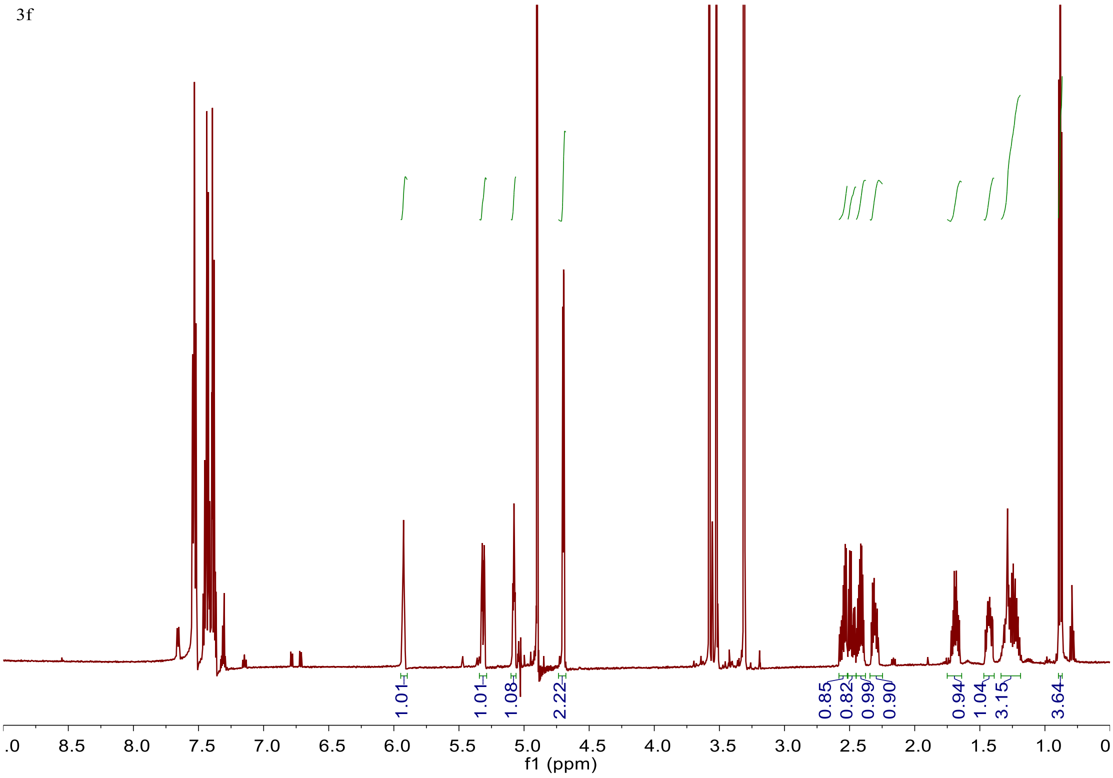

Figure S91. ^{1}H NMR (600 MHz, CD_3OD) spectrum of **3e** and **3f**

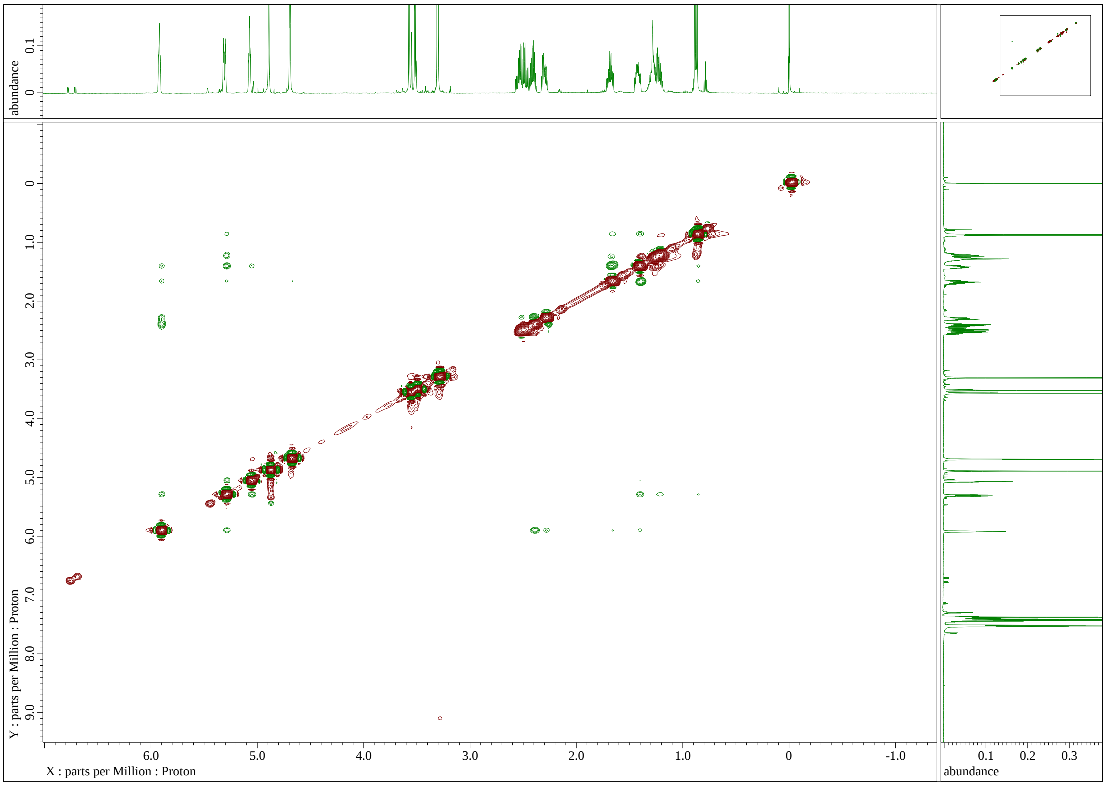

Figure S92. NOESY (CD_3OD) spectrum of **3f**

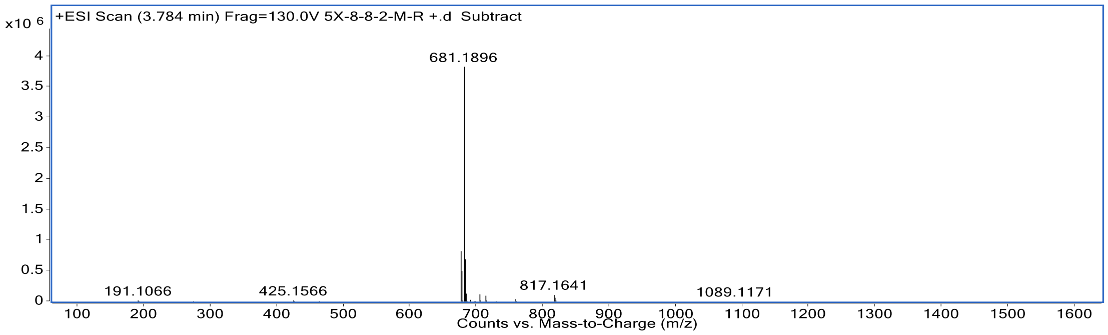

Figure S93. HRESIMS spectrum of **3e**

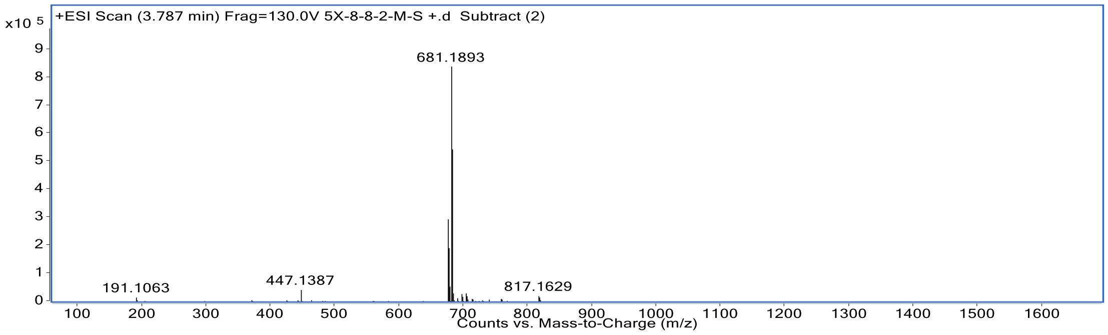

Figure S94. HRESIMS spectrum of **3f**

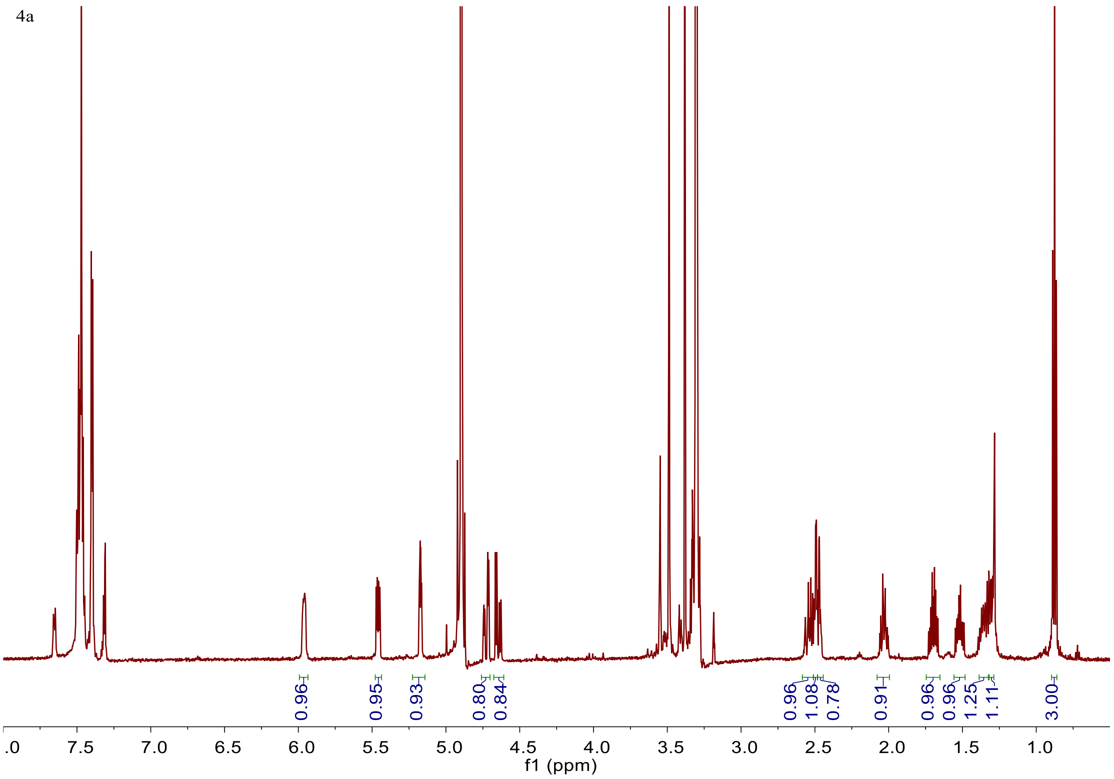

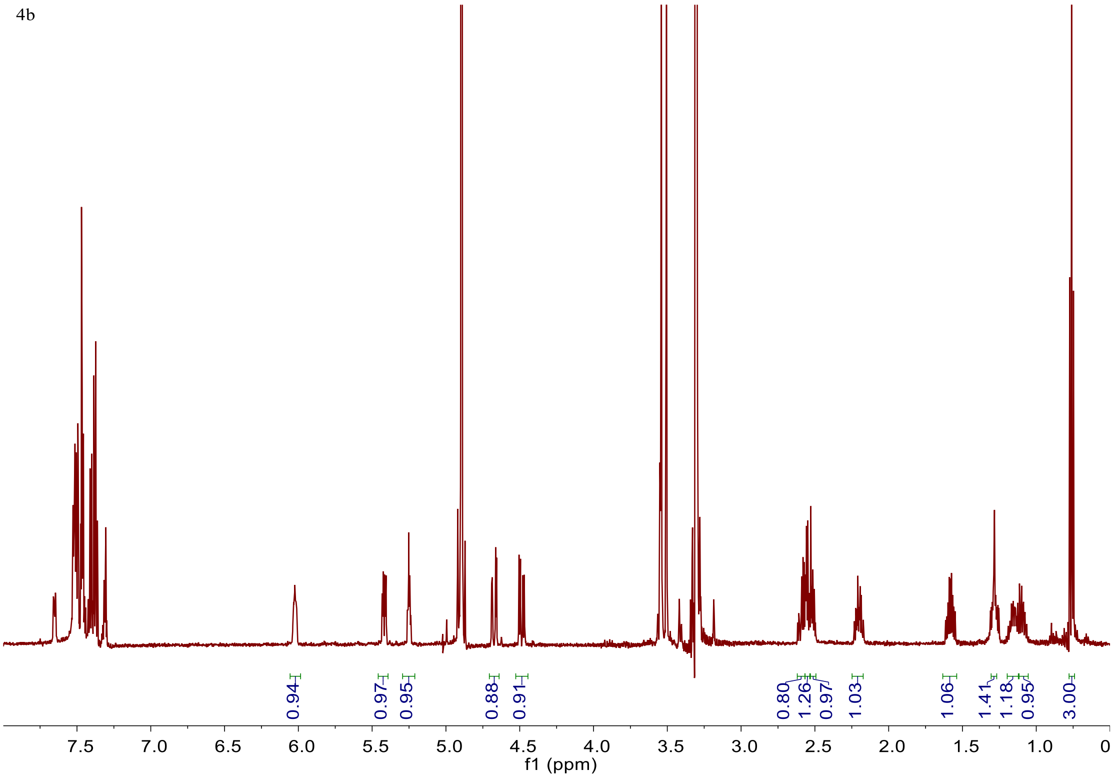

Figure S95. ^{1}H NMR (600 MHz, CD_3OD) spectrum of **4a** and **4b**

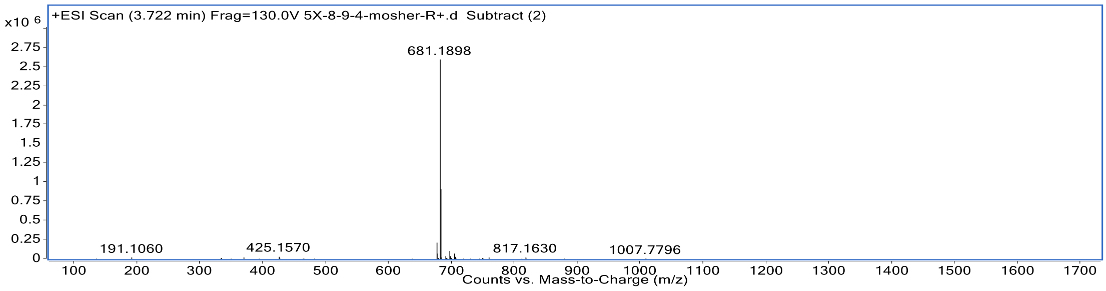

Figure S96. HRESIMS spectrum of **4a**

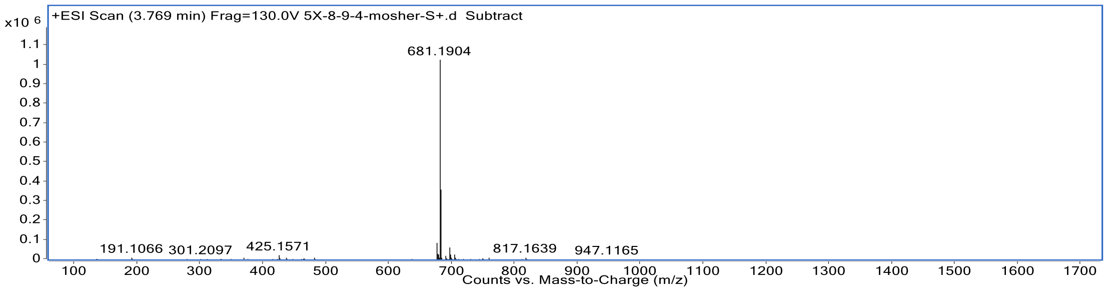

Figure S97. HRESIMS spectrum of **4b**

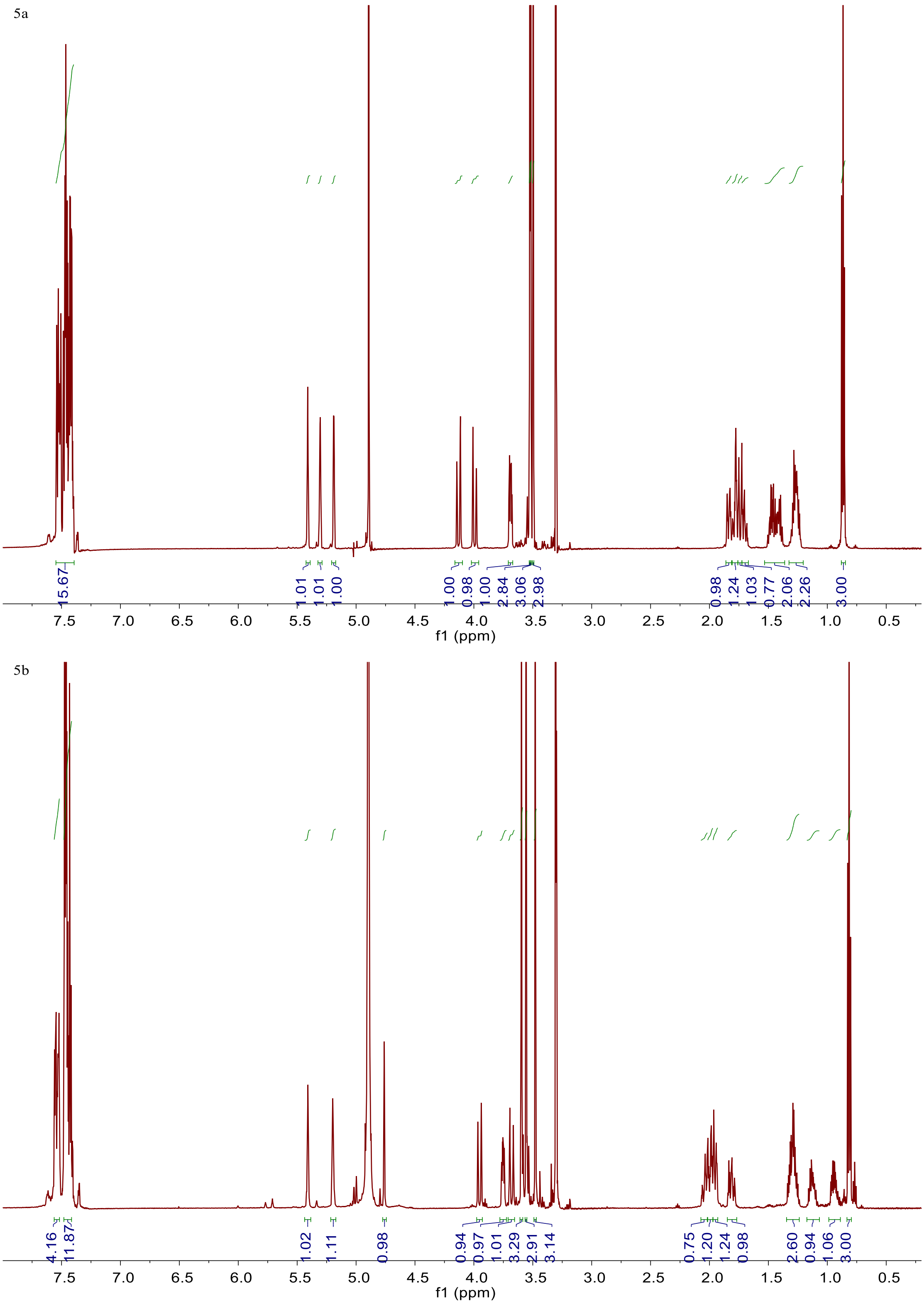

Figure S98. ^{1}H NMR (600 MHz, CD_3OD) spectrum of **5a** and **5b**

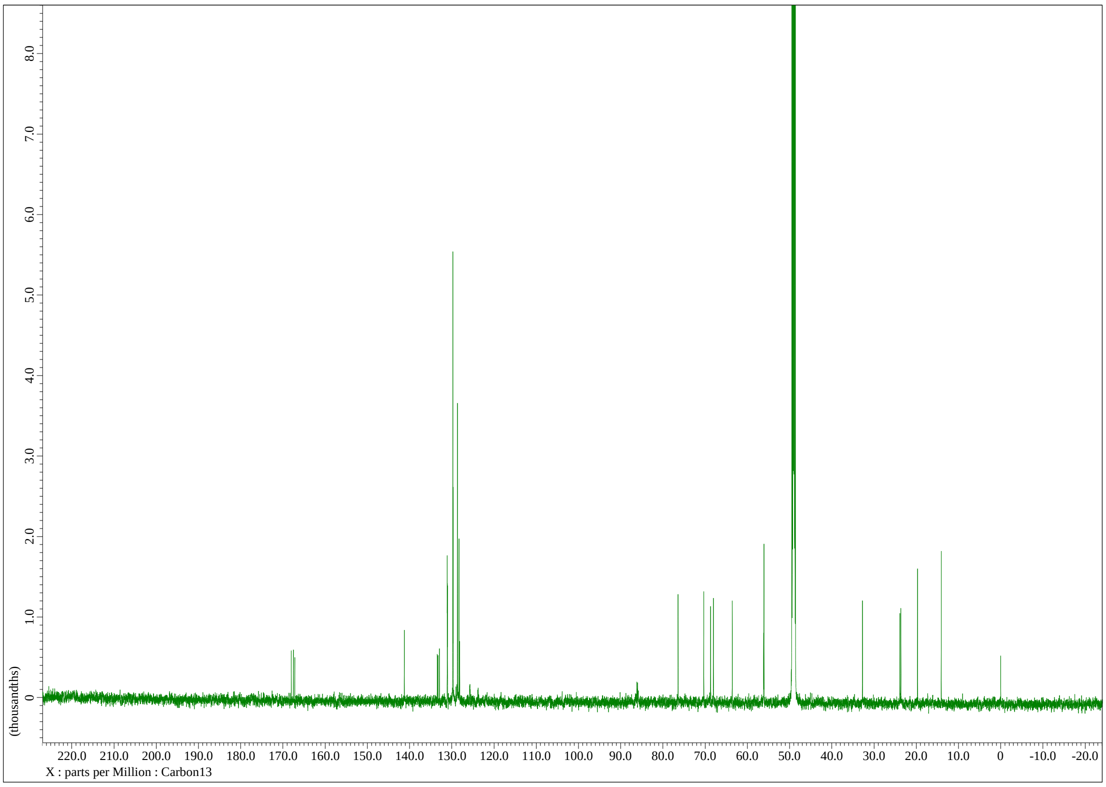

Figure S99. ^{13}C NMR (150 MHz, CD_3OD) spectrum of **5a**

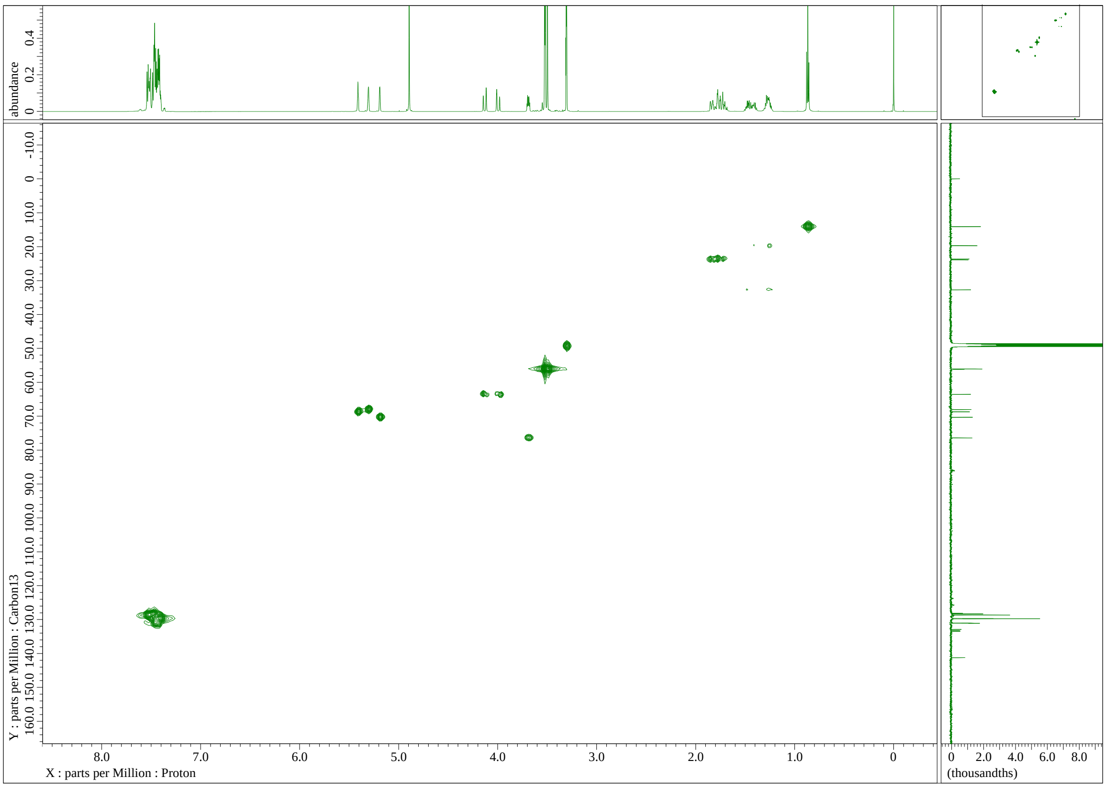

Figure S100. HSQC (CD_3OD) spectrum of **5a**

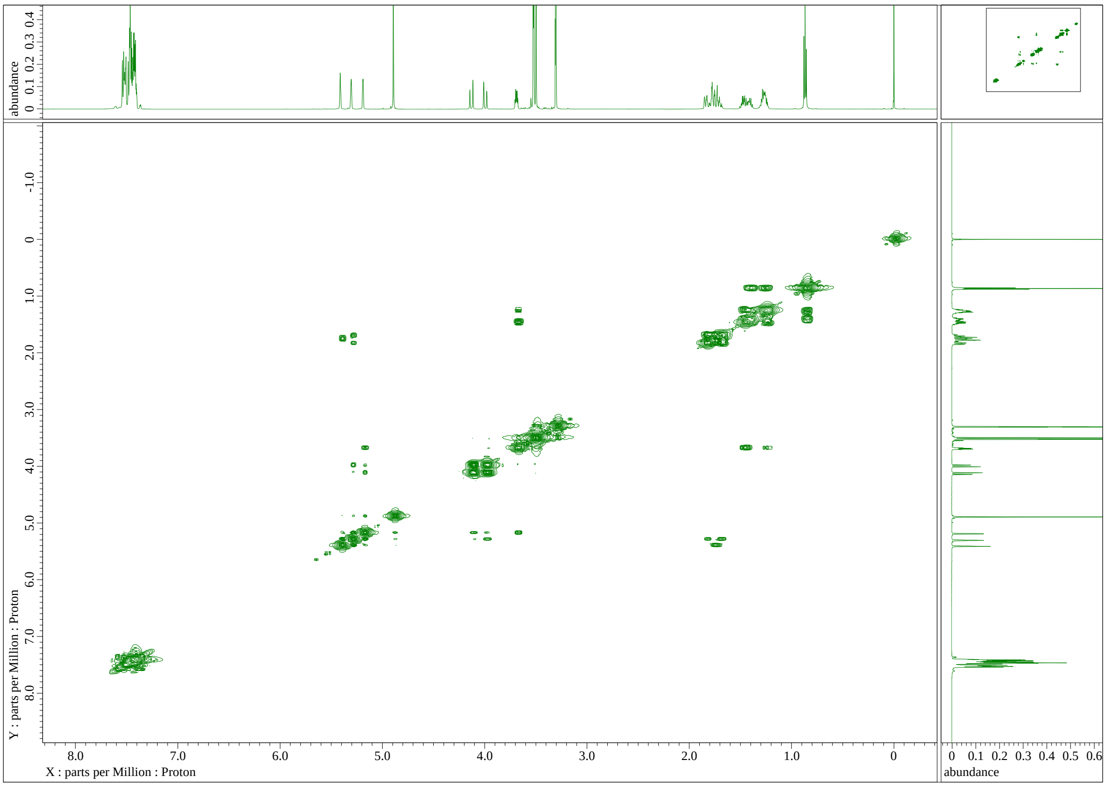

Figure S101. COSY (CD_3OD) spectrum of **5a**

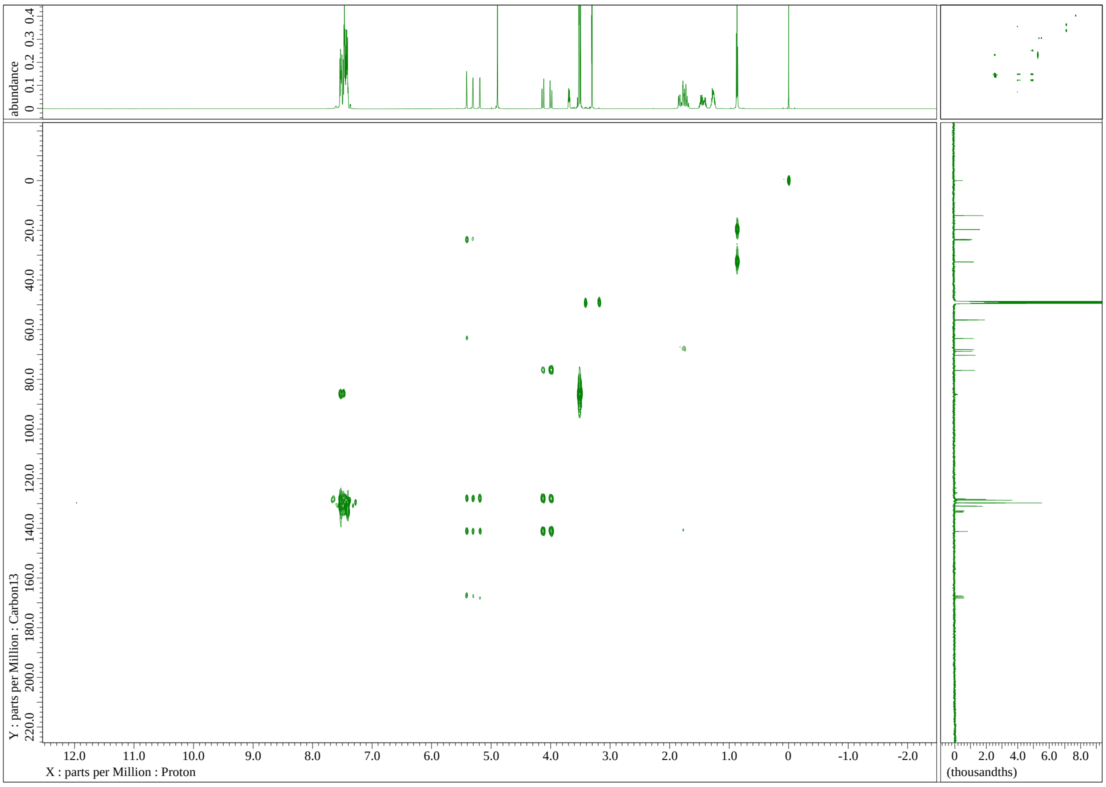

Figure S102. HMBC (CD_3OD) spectrum of **5a**

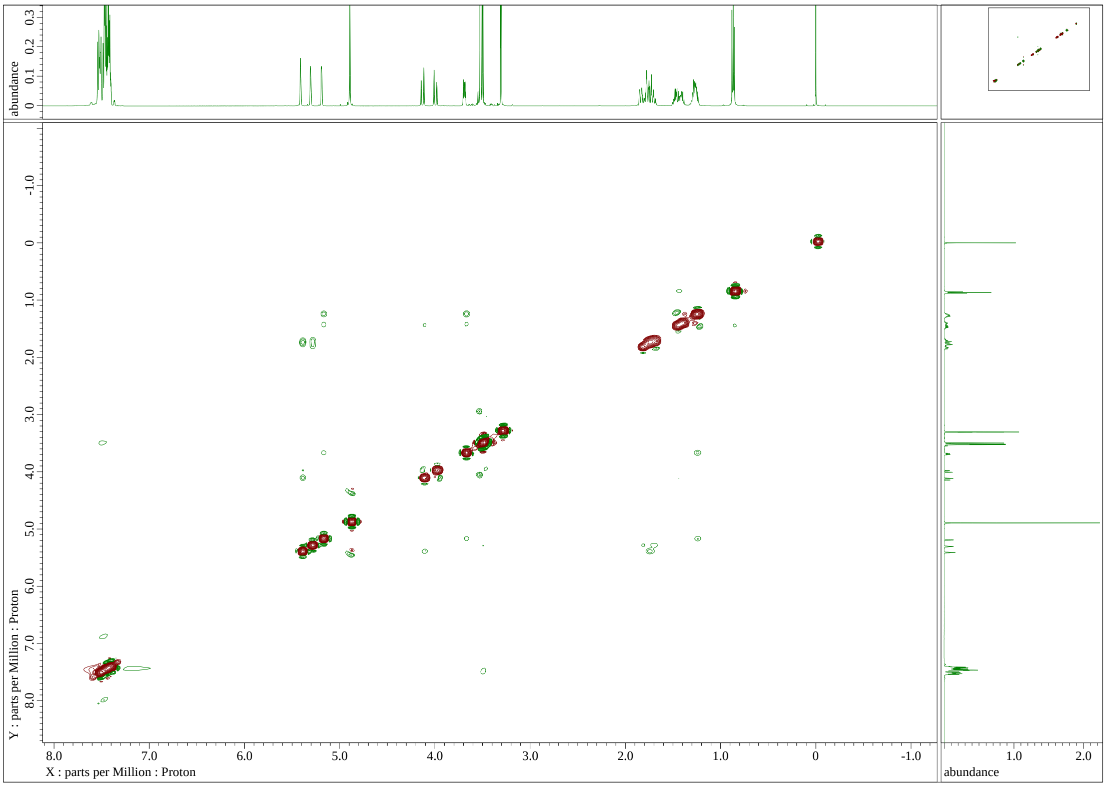

Figure S103. NOESY (CD_3OD) spectrum of **5a**

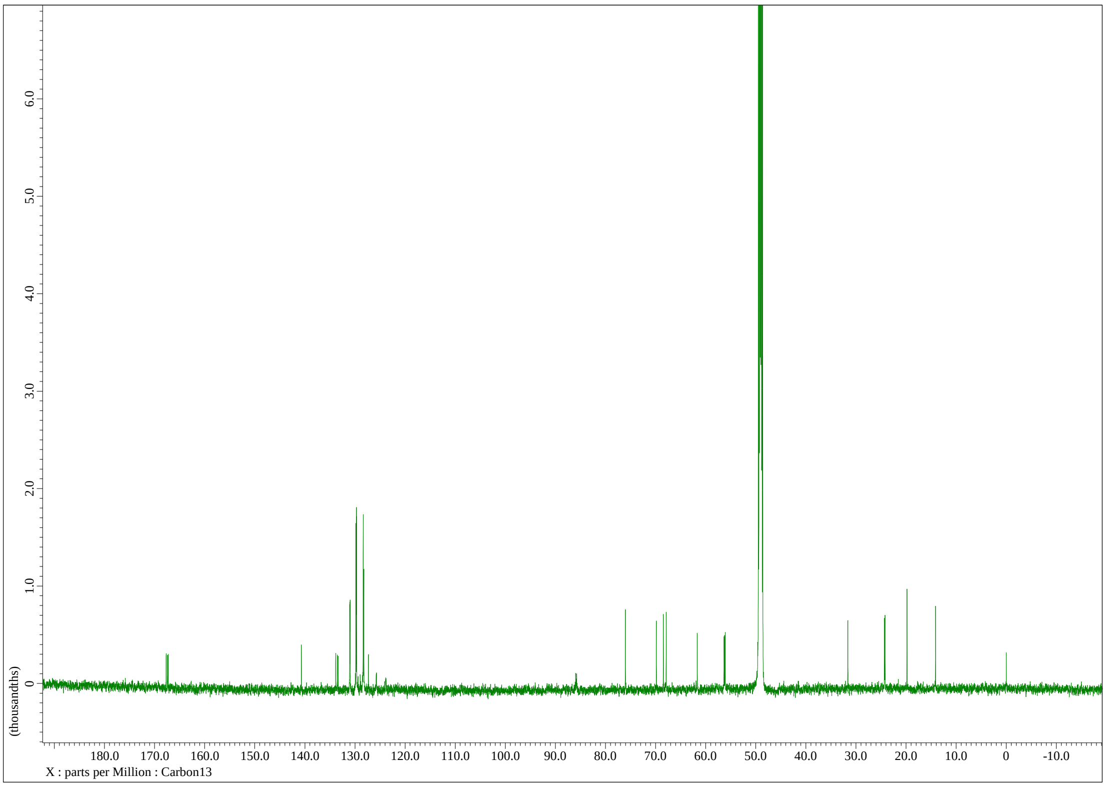

Figure S104. ^{13}C NMR (150 MHz, CD_3OD) spectrum of **5b**

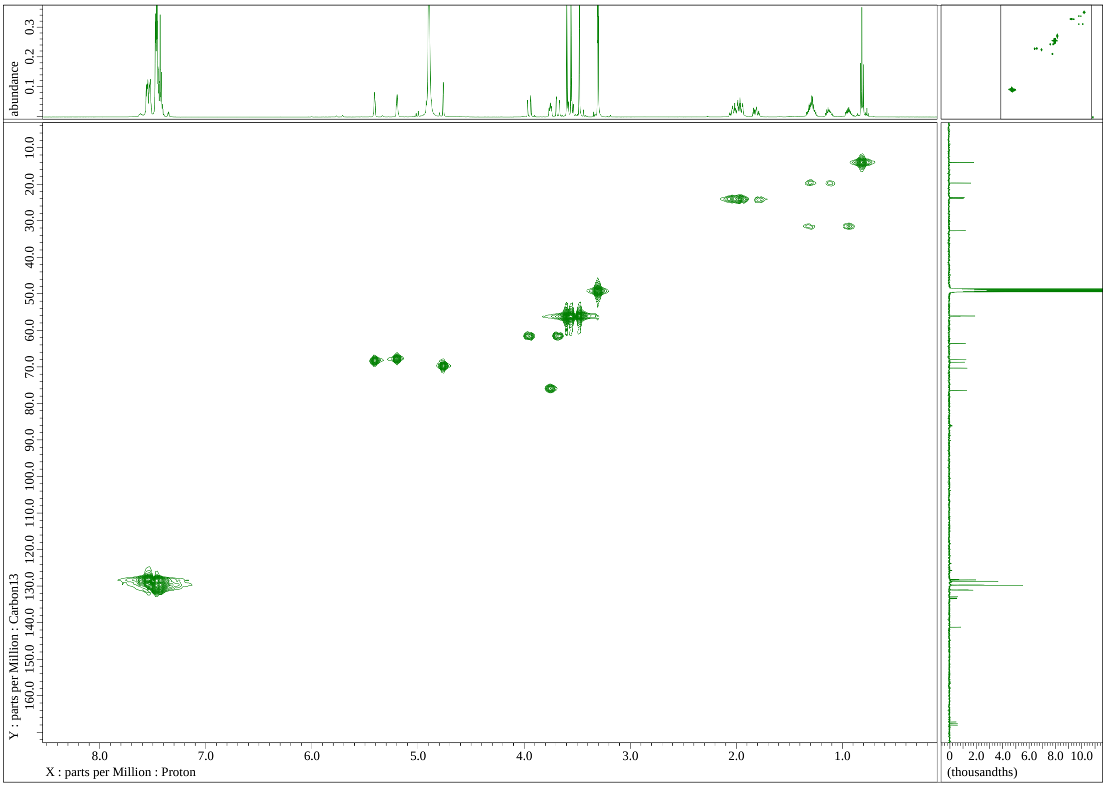

Figure S105. HSQC (CD_3OD) spectrum of **5b**

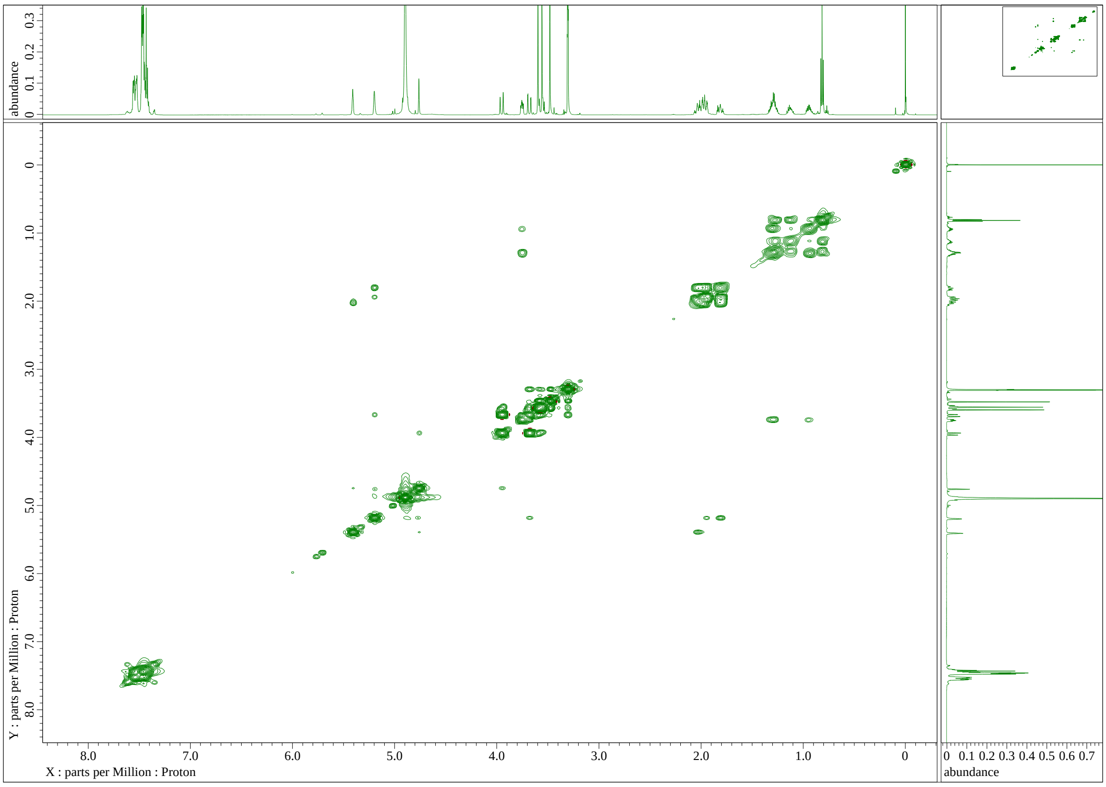

Figure S106. COSY (CD_3OD) spectrum of **5b**

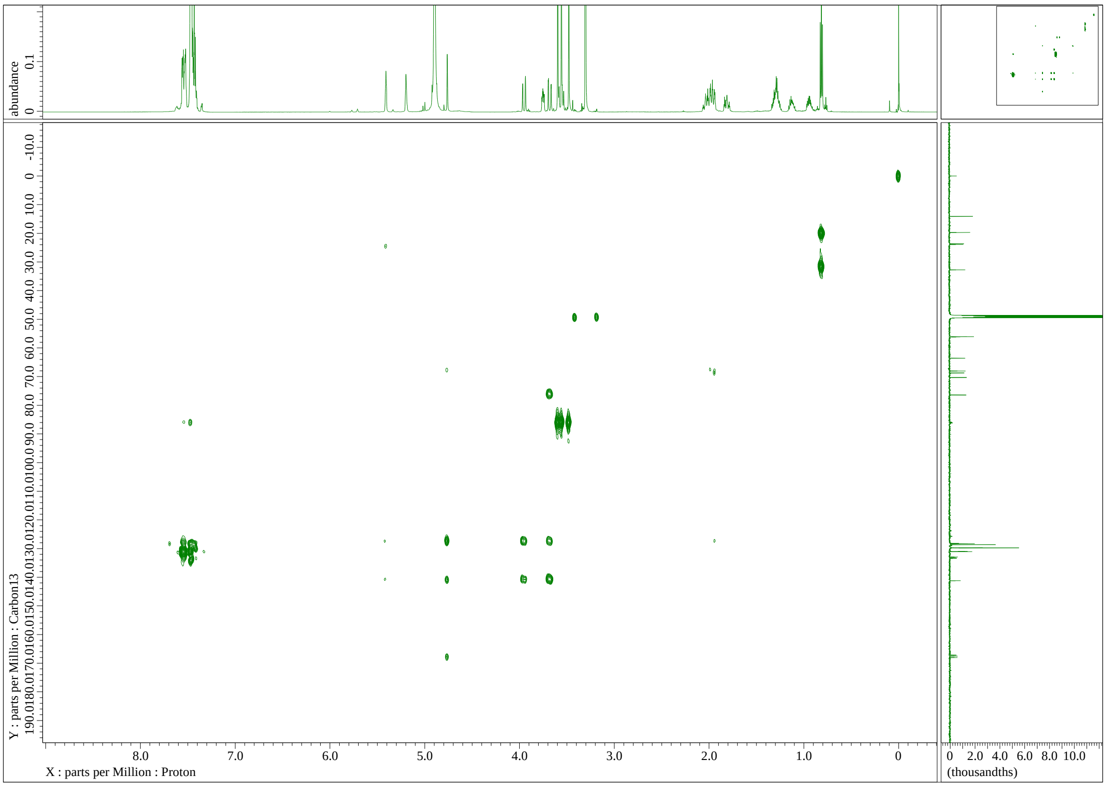

Figure S107. HMBC (CD_3OD) spectrum of **5b**

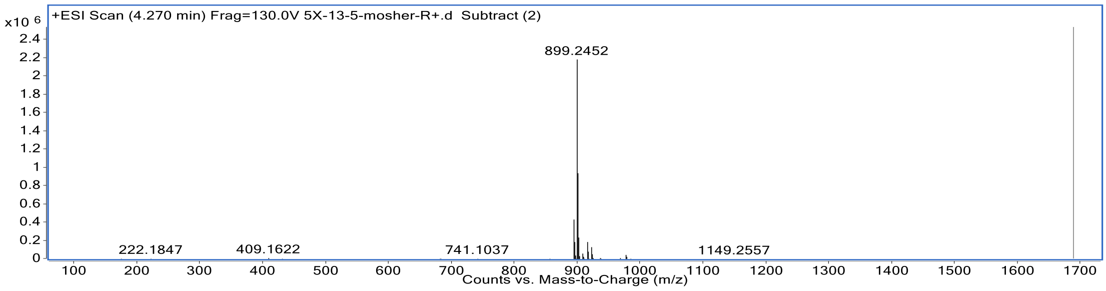

Figure S108. HRESIMS spectrum of **5a**

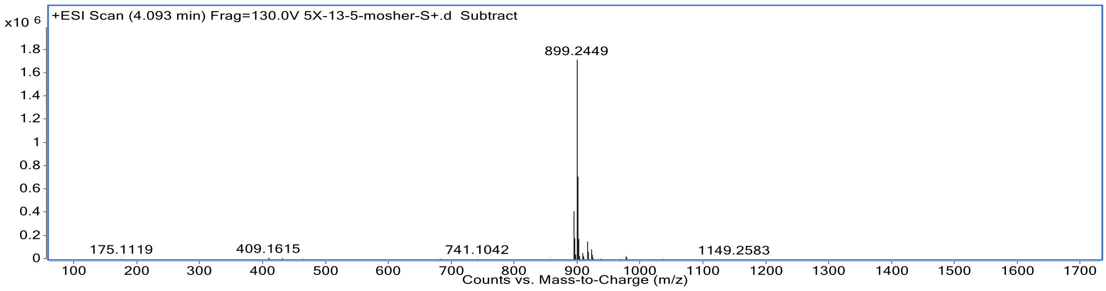

Figure S109. HRESIMS spectrum of **5b**

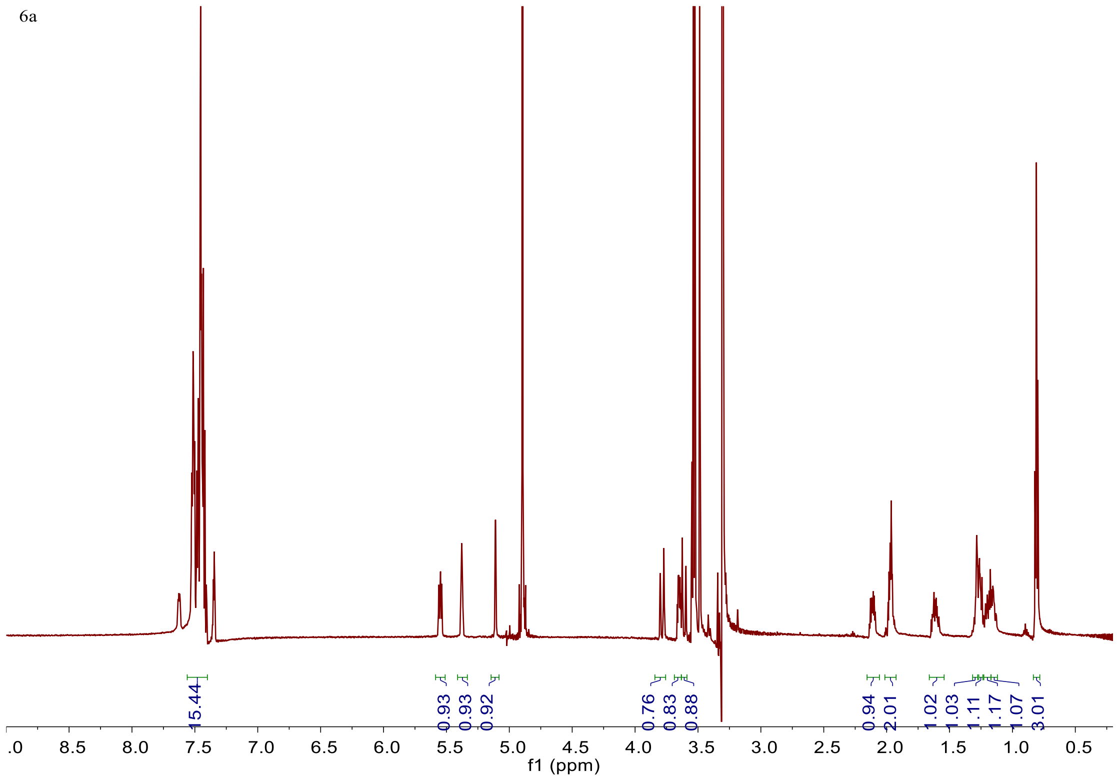

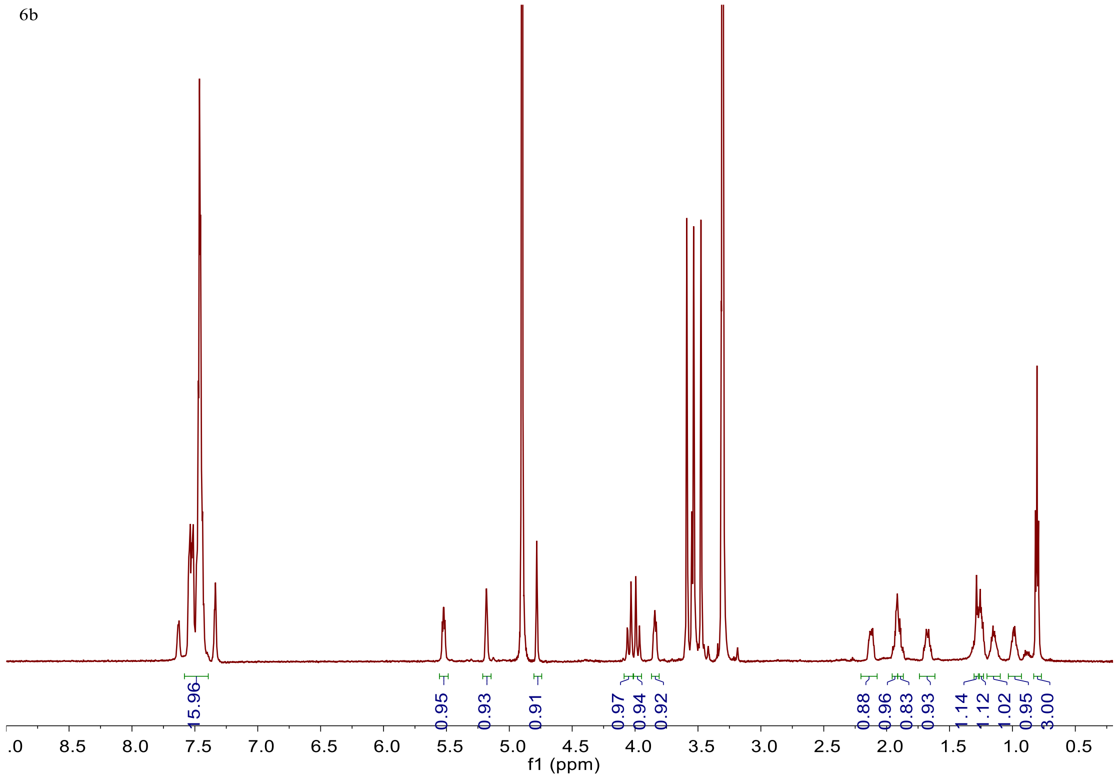

Figure S110. ^{1}H NMR (600 MHz, CD_3OD) spectrum of **6a** and **6b**

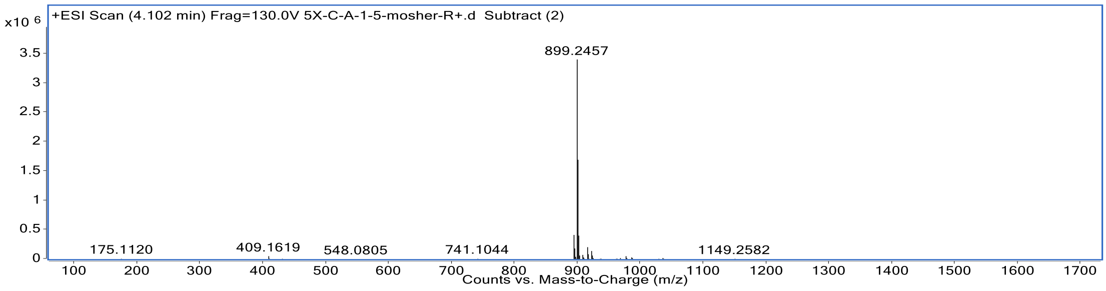

Figure S111. HRESIMS spectrum of **6a**

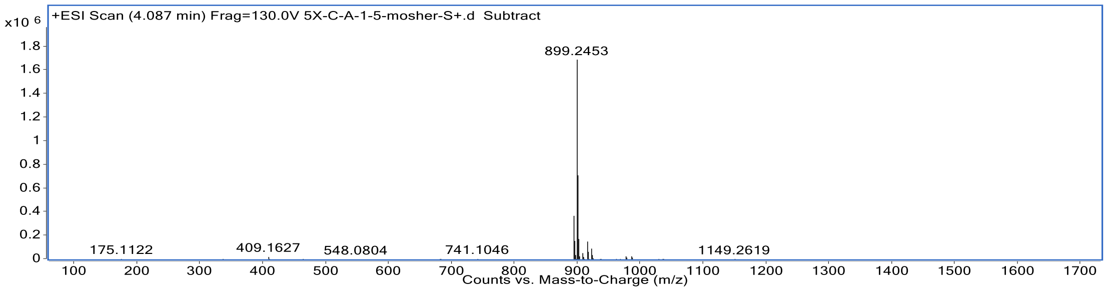

Figure S112. HRESIMS spectrum of **6b**

The 26s rDNA sequence information of *Letendraea* sp.

CCTAGTAACGGCGAGTGAAGCGGCAACAGCTCAAATTTGAAATCTGGCTCCTTTGGG
AGTCCGAGTTGTAATTTGCAGAGGGTGCTTTGGCATAGGCGGCGGTCTAAGTTCCTTG
GAACAGGACATCGCAGAGGGTGAGAATCCCGTACGTGGGCGCCTGCCTTTGCCGTGT
AAAGCCCCTTCGACGAGTCGAGTTGTTTGGGAATGCAGCTCTAAATGGGAGGTAAATT
TCTTCTAAAGCTAAATACCGGCCAGAGACCGATAGCGCACAAGTAGAGTGATCGAAA
GATGAAAAGTACTTTGGAAAGAGAGTCAAAAAGCACGTGAAATTGTTGAAAGGGAA
GCGCTTGCAGCCAGACTTGCCCGCAGTTGCTCACCTAGGCTTCGGCCTGGGGCACTCT
TCTGCGGGCAGGCCAGCATCAGTTTGGGCGGTTGGATAAAGGCCTCTGTCACGTATCT
CTCTTCGGGGAGACCTTATAGGGGAGGCGTAATGCAACCAGCCCGGACTGAGGTCCG
CGCATCTGCTAGGATGCTGGCGTAATGGCTGTAAGCGGC
